# Noncovalent Interactions in the Molecular Geometries of 4-Methylthiazole···H_2_O and 5-Methylthiazole···H_2_O Revealed by Microwave Spectroscopy

**DOI:** 10.1021/acs.jpca.3c05360

**Published:** 2023-09-26

**Authors:** Charlotte N. Cummings, Isabelle Kleiner, Nicholas R. Walker

**Affiliations:** † Chemistry- School of Natural and Environmental Sciences, Newcastle University, Bedson Building, Newcastle-upon-Tyne, NE1 7RU, U.K.; ‡ Université de Paris and Université Paris Est Creteil, CNRS, LISA, F-75013 Paris, France

## Abstract

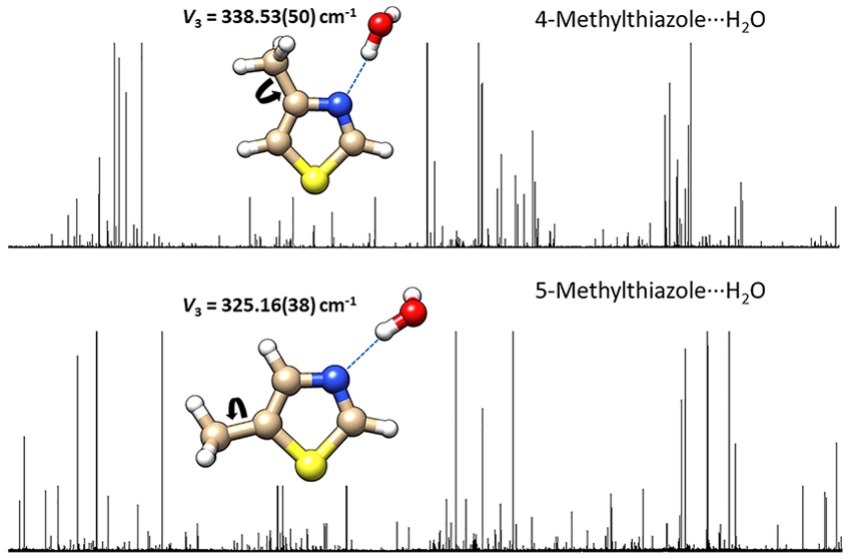

The pure rotational spectra of 4-methylthiazole···H_2_O and 5-methylthiazole···H_2_O were recorded by chirped-pulse Fourier transform microwave (CP-FTMW) spectroscopy. Each complex was generated within the rotationally cold environment of a gas sample undergoing supersonic expansion in the presence of an argon buffer gas. The spectra of five isotopologues of each complex have been measured and analyzed to determine the rotational constants, *A*_0_, *B*_0_, and *C*_0_; centrifugal distortion constants, *D*_*J*_, *D*_*JK*_, and *d*_1_; nuclear quadrupole coupling constants, *χ*_*aa*_(N3) and [*χ*_*bb*_(N3) – *χ*_*cc*_(N3)]; and parameters describing the internal rotation of the CH_3_ group, *V*_3_ and **∠**(*i*,*b*). The experimentally deduced parameters were obtained using the XIAM and the BELGI-C_s_-hyperfine code. For each complex, parameters in the molecular geometry are fitted to experimentally determined moments of inertia. DFT calculations have been performed at the ωB97X-D/aug-cc-pVQZ level in support of the experiments. Each complex contains two hydrogen bonds; a comparatively strong, primary interaction between the N of thiazole and an O–H of H_2_O, and a weaker, secondary interaction between O and either the hydrogen atom attached to C2 (in 5-methylthiazole···H_2_O) or the CH_3_ group attached to C4 (in 4-methylthiazole···H_2_O). The barrier to internal rotation of the CH_3_ group, *V*_3_, is slightly lower for 4-methylthiazole···H_2_O (XIAM result is 340.05(56) cm^–1^) than that for the 4-methylthiazole monomer (357.6 cm^–1^). This is likely to be a result of internal charge redistribution within the 4-methylthiazole subunit following its coordination by H_2_O. At the precision of the experiments, *V*_3_ of 5-methylthiazole···H_2_O (XIAM result is 325.16(38) cm^–1^) is not significantly different from *V*_3_ of the 5-methylthiazole monomer (332.0 cm^–1^).

## 1 Introduction

Thiazole is a five-membered ring that contains sulfur and nitrogen heteroatoms. Its derivatives are found in a range of naturally occurring and synthetic compounds. Vitamin B1, also known as thiamine, is a thiazole-containing compound and essential micronutrient necessary for normal functioning of the nervous system while not being directly synthesized by human metabolism.^1^ Thiazole rings are present in epothilones, a large class of potential anticancer drugs.^2^ Numerous studies of thiazole and its derivatives have been conducted using microwave spectroscopy, a powerful tool for investigating the molecular structure and internal dynamics.

**Figure 1:**
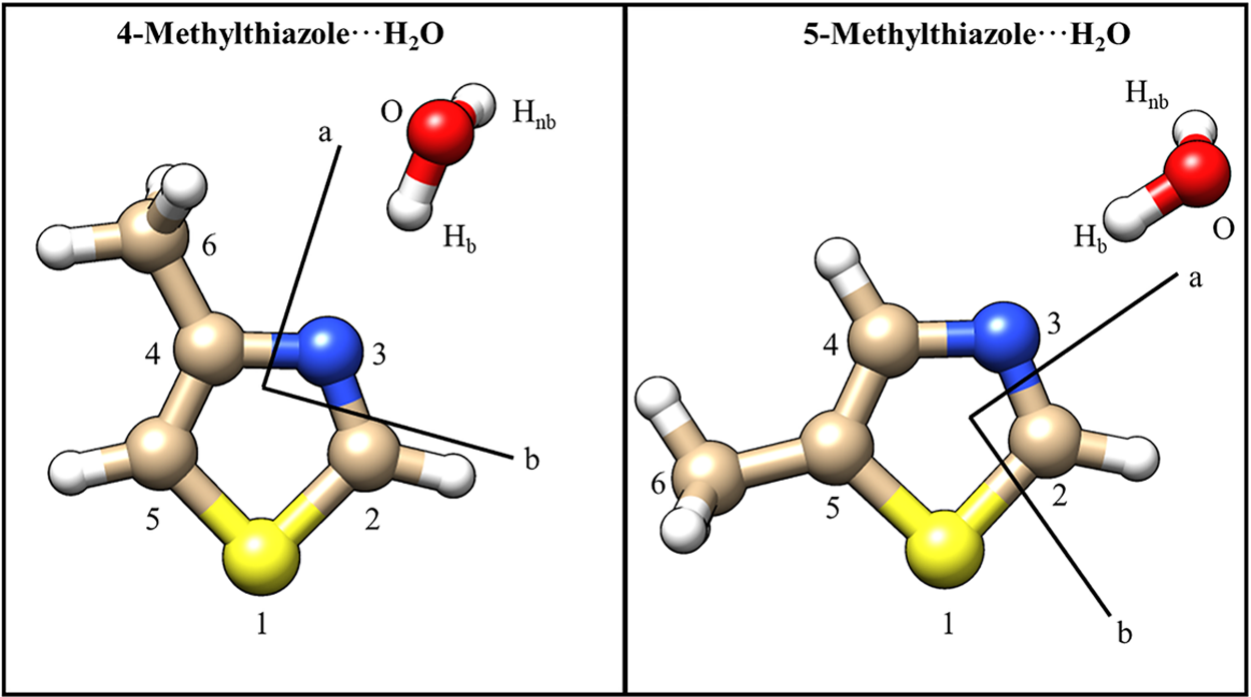
Equilibrium (*r*_e_) geometries of 4-MT···H_2_O (left) and 5-MT···H_2_O (right) calculated at the ωB97X-D/aug-cc-pVQZ level of theory.

Thiazole was first studied by this method in 1962 by Bak et al.^3^ Subsequent works analyzed the spectra of many isotopologues and allowed the determination of its molecular geometry.^4,5^ Studies have since explored how the energy barrier to internal rotation of the CH_3_ group varies across the 2-methylthiazole,^6,7^ 4-methylthiazole,^8^ and 5-methylthiazole^9^ series of isomers. The magnitude of this barrier is denoted as *V*_3_ and has been reported to be 34.1 cm^–1^ for 2-methylthiazole, 357.6 cm^–1^ for 4-methylthiazole, and 332.0 cm^–1^ for 5-methylthiazole. Similar studies have been performed for isomers of methylimidazole,^10^ methylpyrrole,^11−13^ methyloxazole,^14,15^ methylisoxazole,^16,17^ methylfuran,^18−20^ methylisothiazole,^21^ and methylthiophene.^22−24^ Generally, for five-membered heteroaromatic rings containing two heteroatoms, the lowest barrier is observed where CH_3_ is substituted onto the 2-position (the convention for atom numbering is shown in Figure 1). Higher barriers are observed where CH_3_ is substituted onto the 4- or 5- positions because of differences in electronic structure. The identity of the heteroatoms is important, particularly where methylation is at the 2-position. For example, the (*V*_3_) barrier is 122.8 cm^–1^ for 2-methylimidazole whereas for 2-methyloxazole it is 251.8 cm^–1^. The monohydrate complex of thiazole was reported in 2020 by W. Li et al.^25^ The primary hydrogen-bonding interaction within this complex is between the nitrogen atom of the thiazole moiety which acts as the hydrogen bond acceptor and an O–H of the water subunit which acts as hydrogen bond donor. The authors of this previous study did not invoke the presence of a weak hydrogen bond between the O atom of H_2_O and a hydrogen atom located on C2 of thiazole, but such an interaction was proposed for the similar complex, imidazole···H_2_O.^26^

The present work will explore the noncovalent interactions that are present when H_2_O coordinates to each of two isomers of methylthiazole. It will compare and contrast the noncovalent interactions within 4-methylthiazole···H_2_O and 5-methylthiazole···H_2_O (hereafter denoted as 4-MT···H_2_O and 5-MT···H_2_O respectively) and explore the implications of these for molecular geometry and the *V*_3_ barriers to internal rotation of the CH_3_ groups. In addition to a primary hydrogen bond between the N of thiazole and an O–H of water, it will be shown that a weak hydrogen bond forms between the O atom of H_2_O and the CH_3_ group in 4-MT···H_2_O. The value of *V*_3_ for the 4-MT···H_2_O complex is slightly *lower* than that for the 4-methylthiazole monomer. This is proposed to result from minor changes in the electronic structure within 4-methylthiazole on coordination by a H_2_O molecule. It will be shown that the values of *V*_3_ for the 5-methylthiazole monomer and for 5-MT···H_2_O are not significantly different. The results of the present work will be compared with those reported previously for *N*-methylimidazole···H_2_O and 2-methylimidazole···H_2_O.^27^ This previous work reported that there is no significant difference between *V*_3_ for *N*-methylimidazole and *N*-methylimidazole···H_2_O. In contrast, *V*_3_ was found to be significantly *higher* for 2-methylimidazole···H_2_O than for the 2-methylimidazole monomer as a result of a weak electrostatic interaction (a hydrogen bond) between the O atom of H_2_O and the CH_3_ group of 2-methylimidazole.

## 2 Experimental and Theoretical Methods

The microwave spectra of 4-MT···H_2_O and 5-MT···H_2_O were recorded in separate experiments. Each experiment was performed while probing a gaseous sample containing a low concentration of the methylthiazole sample (4-methylthiazole or 5-methylthiazole as appropriate to the complex under study) and water in an argon carrier gas. 4-Methylthiazole (Sigma-Aldrich, 99%) and 5-Methylthiazole (TCI Chemicals, 98%) are liquids under ambient conditions and were used in experiments without any further purification. The experimental procedure used to study each 4-MT···H_2_O and 5-MT···H_2_O was as follows. A methylthiazole sample (0.3 mL) was syringed onto glass wool within a bespoke reservoir^28^ which was heated to 50–60 °C (depending on the isomer being studied) so as to introduce methylthiazole into the flow of argon (BOC, 99.998%) immediately prior to supersonic expansion of the gas mixture into a vacuum chamber from a pulsed nozzle (Parker, Series 9). Species within the supersonic expansion acquire a rotational temperature of (typically) ∼ 3 K. A second reservoir, located approximately 20 cm before the supersonic expansion, contained glass wool and was used to introduce water into the carrier gas flow. Experiments were performed using D_2_O (Sigma-Aldrich, 99.9% D atom) and H_2_^18^O (Sigma-Aldrich, 97% ^18^O atom) when performing experiments to study D- and ^18^O-containing isotopologues. All experiments were performed using a backing pressure of 1 bar of argon, which was found to be sufficient to allow recording of the spectra of both the methylthiazole monomer and the water-containing complexes.

The rotational spectra of 4-MT···H_2_O and 5-MT···H_2_O were recorded over the 7.0–18.5 GHz frequency range using the Chirped-pulse Fourier Transform Microwave (CP-FTMW) spectrometer at Newcastle University which has been described in detail elsewhere.^29^ A chirped pulse of microwave radiation that sweeps the frequency range, 12–0.5 GHz, over a duration of 1 μs, is generated from a 20 GS s^–1^ Arbitrary Waveform Generator (AWG) (Tektronix AWG 7102). The microwave radiation is mixed against a 19 GHz reference signal provided by a Phased Locked Dielectric Resonant Oscillator (PDRO), the lower frequency sideband (7.0–18.5 GHz) is selected by a low pass filter and the higher frequency sideband (19.5–31 GHz) is subsequently removed. The microwave radiation is amplified using a 300 W Traveling Wave Tube Amplifier (TWTA) before being broadcast into the vacuum chamber via a horn antenna. The pulse of microwave radiation intersects the expanding gas sample and rotationally polarizes molecules and complexes on resonance with rotational transitions. The free induction decay (FIDs) of the molecular emission is then recorded at a second horn antenna over a duration of 20 μs. Eight FID’s per nozzle pulse are digitally recorded by a 100 GS s^–1^ oscilloscope (Tektronix DPO72304XS). All FID’s are coadded together in the time domain before a Fourier transform of the data is performed using a Kaisser-Bessel window function. A line width of 100 kHz is achieved for an isolated line at full width at half-maximum with an estimated accuracy of 10 kHz in the line center frequencies in the frequency domain spectrum. Phase coherence in the time domain and accuracy in transition frequencies were provided by an Analog Signal Generator (Agilent MXG N5183A) to which the AWG, the PDRO and the oscilloscope were phase-locked.

## 3 Density Functional Theory Calculations

Quantum chemical calculations were performed using the Gaussian09 package.^30^ First, the geometries of 4-MT···H_2_O and 5-MT···H_2_O were calculated as follows. The optimized geometry of the methylthiazole monomer was positioned alongside H_2_O to anticipate a hydrogen bond between an O–H of H_2_O and the nitrogen atom of the heteroaromatic. Calculations were then performed at three different levels of theory. Initially, geometry optimizations were performed using the harmonic hybrid functional^31−33^ of Becke, Lee, Yang, and Parr (B3LYP), in conjunction with Grimmes dispersion correlation effects^34^ and damping function,^35^ D3BJ, alongside Dunning’s augmented triple-ζ aug-cc-pVTZ basis set.^36,37^ Optimization calculations were subsequently performed using the long-range corrected hybrid functional,^38^ ωB97X-D, with very tight convergence criteria and Dunning’s quadrupole-ζ aug-cc-pVQZ basis set. These basis sets and functionals were previously used in studies of monohydrate complexes of imidazole and methylimidazole. Calculations were also performed using second order Møller–Plesset perturbation theory^39^ with Dunning’s augmented double-ζ aug-cc-pVDZ basis set. Potential energy scans were performed by scanning the **∠**(H–C6–C4–N3) and **∠**(H–C6–C5–C4) dihedral angles for 4-MT···H_2_O and 5-MT···H_2_O respectively. The (*V*_3_) barrier to internal rotation of the CH_3_ group was calculated for each complex. The optimized geometries of the monohydrate complexes calculated at the ωB97X-D/aug-cc-pVQZ level are shown in Figure 1 with the associated atomic coordinates calculated at several levels of theory provided in Tables S1–S3 of the Supporting Information. For each complex, calculations were performed to explore the stabilities of higher energy conformers similar to those for which geometries were calculated in a recent study of thiazole···H_2_O.^25^ Each of these calculations (performed at the B3LYP(D3BJ)/aug-cc-pVTZ level) did not converge. The rotational constants (*A*_*e*_, *B*_*e*_, *C*_*e*_), nuclear quadrupole coupling constants (*χ*_*aa*_(N3), [*χ*_*bb*_(N3) – *χ*_*cc*_(N3)]), electric dipole moment components (|*μ*_*a*_|, |*μ*_*b*_|, |*μ*_*c*_|) and *V*_3_ barrier are summarized in Table S4, which also reports the percentage deviation between the experimentally determined and calculated parameters.

## 4 Results

### Spectral Assignment and Analysis

**Figure 2:**
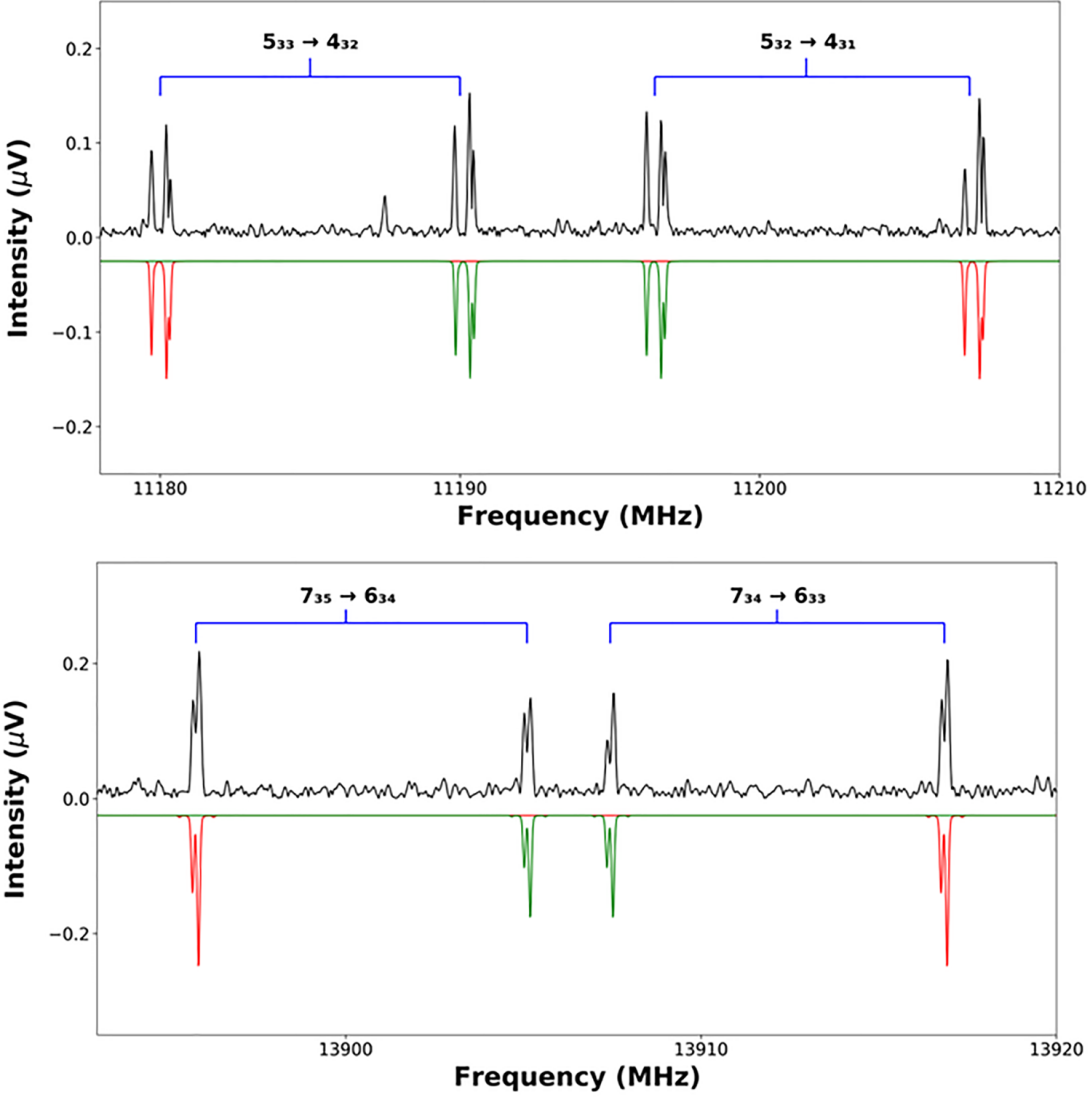
Small section of the rotational spectrum of 4-MT···H_2_O (upper panel) and 5-MT···H_2_O (lower panel). Two rotational transitions of each complex are displayed. The experimental spectrum (black) is shown above a PGOPHER simulation (red and green). The simulated *E*-species spectrum (green) was generated by manually offsetting (using the PGOPHER “offset” function) the frequencies of transitions in the *A*-species spectrum by appropriate frequency intervals as determined by the XIAM fit.

Transitions of the methylthiazole monomer (4-methylthiazole or 5-methylthiazole as appropriate for the experiment being performed) were the most intense features in the spectra recorded. A transition of the water dimer, (H_2_^16^O)_2_, at 12321 MHz was also consistently observed with high intensity during the initial experiments. For each of 4-MT···H_2_O and 5-MT···H_2_O, the optimized geometry (Figure 1) was calculated to be a near-prolate asymmetric top. Assignments of transitions to spectra of 4-MT···H_2_^16^O and 5-MT···H_2_^16^O were initially made on the basis of good agreement between experimentally determined spectroscopic constants and those determined by DFT calculations. Initial fits exclusively considered *A*-species (σ = 0) transitions and employed Watson’s S-reduced Hamiltonian^40^ as implemented by Western’s PGOPHER^41^ program
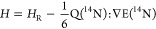
where *H*_R_ is the energy operator for a semirigid asymmetric rotor in its vibrational ground state. This term includes the effective rotational constants *A*_0_′, *B*_0_′, and *C*_0_′ and centrifugal distortion constants *D*_*J*_, and *D*_*JK*_. The additional centrifugal distortion parameter, *d*_1_, was included to fit the data for 4-MT···H_2_O with a satisfactory accuracy. Transitions of 4-MT···H_2_O and 5-MT···H_2_O exhibit a hyperfine structure owing to the presence of the nitrogen nucleus (*I* = 1 for ^14^N) within the thiazole ring. Hence, the second term within the Hamiltonian describes the interaction between the nuclear electric quadrupole moment, *Q*(^14^N), and the electric field gradient, ∇*E*(^14^N), at the nitrogen nucleus. Matrix elements are constructed in the (*I*_N_ + *J* = *F*) basis and diagonalized in blocks of the quantum number, *F*. Values of nuclear quadrupole coupling constants are displayed in Tables S5 and S6 give good agreement between hyperfine structure observed in the experimental spectra and simulations prepared using PGOPHER (as shown in Figure 2). Tunneling splittings were not resolved nor assigned during the present work.

Only *a*-type transitions were assigned for each of 4-MT···H_2_O and 5-MT···H_2_O which is consistent with expectations based on the calculated orientation of the dipole moment for each complex shown in Figure 1. When calculated at the ωB97X-D/aug-cc-pVQZ level for 4-MT···H_2_O and 5-MT···H_2_O respectively, the values of *μ*_*a*_ are 2.86 and 4.13 D, those for *μ*_*b*_ are 0.75 and 0.49 D and those for *μ*_*c*_ are 1.36 and 1.30 D. The rapid zero-point vibrational motion of the complex (explained below under Molecular Geometry) will mean that the average of *μ*_*c*_ in the zero-point state is much lower than implied by the *r*_e_ result quoted above. For each complex, therefore, the projection of the dipole moment onto the *a*-inertial axis leads to *μ*_*a*_ having a significantly greater magnitude than either *μ*_*b*_ or *μ*_*c*_. Transition intensities are proportional to the square of the dipole moment, so it is not surprising that *b*- and *c*-type transitions were not identified for either complex despite careful searching. It was confirmed that the spectra of each of 4-MT···H_2_O and 5-MT···H_2_O had been assigned to the correct molecular carrier by use of isotopically enriched samples to study isotopologues containing H_2_^18^O, HDO, and D_2_O. The experiments of the present work were exclusively performed using argon carrier gas which is known to encourage relaxation to conformers of low energy within the expanding gas jet. Attempts were made to assign the spectra of higher-energy conformers, but these were unsuccessful. It can be noted that an earlier study^25^ of thiazole···H_2_O identified only one isomer of that complex even while measurements were performed using a helium carrier gas more likely to allow for the generation and study of higher-energy isomers.

Diagonalization of the nuclear quadrupole coupling tensor followed by transformation into the framework of principal nuclear axes at the nitrogen atom (using the QDIAG program) allowed *χ*_*xx*_(N3), *χ*_*yy*_(N3), and *χ*_*zz*_(N3) to be determined for each complex. For 4-MT···H_2_O, *χ*_*yy*_(N3), and *χ*_*zz*_(N3) are each slightly lower than those found for each of thiazole and 4-methylthiazole (Table S7). The same is true when *χ*_*yy*_(N3) and *χ*_*zz*_(N3) are compared for 5-MT···H_2_O with the same parameters for thiazole and 5-methylthiazole. The implication is that there is a slight change in the electric field gradient (relative to that of the unhydrated methylthiazole monomer) at the N atom for each complex on attachment of the H_2_O molecule. However, the uncertainties are large relative to the parameter values, so it is not possible to confirm this effect with certainty nor any consequences for the *V*_3_ barriers within the complexes. The value of *χ*_*aa*_(N3) was determined by fitting for every isotopologue of each complex. The result for [*χ*_*bb*_(N3) – *χ*_*cc*_(N3)] was determined only for the parent isotopologue of each complex and was fixed at the result determined for the parent when fits of other isotopologues were performed.

Internal rotation of the CH_3_ group leads to splittings between *A*- and *E*-species transitions in rotational spectra which can be analyzed to determine (*V*_3_) barriers to internal rotation. The published results for the monomers, 4-methylthiazole^8^ and 5-methylthiazole,^9^ allowed for good predictions of *V*_3_ for each of 4-MT···H_2_O and 5-MT···H_2_O such that *E* species transitions were readily identified and assigned in the spectrum of each complex. The assignment was assisted by the observation that each *E*-species, , transition has the same pattern of hyperfine structure as the *A-* species transition of the same  assignment. Examples of hyperfine structure in *A*- and *E*-species transitions are shown in Figure 2. “Global” fits that simultaneously fitted *A*- (σ = 0) and *E*- (σ = ± 1) species transitions were then performed by two alternative methods. The XIAM code uses “combined axis methods” or CAM.^42^ Fits performed in XIAM^43^ employed a Hamiltonian that is first constructed in the principal axis system, then the Hamiltonian matrix is transformed into individual rho axis systems for each internal rotor in order to eliminate Coriolis coupling terms which occur between the internal rotation and the overall rotational angular momenta. At the end, the eigenvalue matrix is transformed back to the principal axis system. XIAM treats each *v*_*t*_ torsional state separately in a “local” approach, without accounting for interactions between different torsional states. The BELGI-C_s_-hyperfine^44,45^ program employs the alternative reference framework of the rho axis system (RAM), and uses a “global” approach, treating all the interaction between different torsional states up to *v*_*t*_ = 8. It also allows the inclusion of higher-order terms between internal and global rotation.

Parameters were fitted using each of XIAM and BELGI-C_s_-hyperfine to explore the dependencies of results on the specific model Hamiltonians. The experiments probed the complexes in their *v*_*t*_ = 0 torsional ground states preventing the independent determination of *F*_0_ which is the inverse of the moment of inertia, *I*_*α*__,_ of the methyl top, and *V*_3_, the first leading term in the Fourier series describing the internal rotation potential. Jäger and Mäder reported that the moments of inertia of the methyl top, *I*_α_, in 4-methylthiazole and 5-methylthiazole are respectively 3.1743(10) and 3.1860(69) u Å^2^ which imply internal rotational constants, *F*_0_, of 159.21(5) GHz and 158.63(34) GHz, respectively.^8,9^ However, we are aware of publications in preparation (as reported informally to other microwave spectroscopists within the *Microwave Spectroscopy Information Letter*, Vol. LXVI) by Koziol and Nguyen, which will provide an updated analysis of the rotational spectra of each of 4-methylthiazole and 5-methylthiazole. Nguyen has privately communicated that their analysis will assume *F*_0_ = 160 GHz for each monomer, using their ab initio value calculated at the MP2(ae)/6-311++G(d,p) level, and the same assumption was employed herein for the XIAM fits. Each BELGI-C_s_-hyperfine fit uses a fixed value for the reduced internal rotation constant *F* defined as  with  where *λ*_*g*_ are the direction cosines of the internal rotation axis *i* of the top in the principal axis system, i.e., *λ*_*g*_ = cos(<(*i,g*)) and *I*_*g*_ are the components of the moments of inertia of the whole molecule in the *g* = *a*, *b*, *c* principal axis system which leads to assumed values for *F* of 161.84 and 164.61 GHz for 4-MT···H_2_O and 5-MT···H_2_O complexes, respectively.

All fits assumed that the axis of the CH_3_ rotor lies in the *ab* plane of each complex. The fits performed using XIAM determined rotational constants (*A*_0_, *B*_0_, and *C*_0_), centrifugal distortion constants (*D*_*J*_, *D*_*JK*_) and nuclear quadrupole coupling constants (χ_*aa*_(N3), [*χ*_*bb*_(N3) – *χ*_*cc*_(N3)]). The additional centrifugal distortion constant, *d*_1_, was included for 4-MT···H_2_O but not for 5-MT···H_2_O. The XIAM fits allowed for determination of the *V*_3_ and ∠(*i*,*b*) internal rotation parameters with root-mean-square (rms) deviations of 22 and 21 kHz for the 4-MT···H_2_^16^O and for 5-MT···H_2_^16^O complexes respectively, i.e., about two times the measurement accuracy expected for unblended lines. The barrier height, *V*_3_, was defined earlier herein and ∠(*i*,*b*) denotes the angle between the axis of the CH_3_ internal rotor and the *b* principal axis. The fits performed using BELGI-C_s_-hyperfine determined *V*_3_ and also ρ and *D*_*ab*_ which are parameters in the vibration–rotation-torsion Hamiltonian used by the analysis.^44^ The ρ parameter is the coupling constant between internal and global rotation and *D*_*ab*_ is related to the angle between the RAM and the PAM axis. In comparison with the XIAM fit, we note that for the BELGI-C_s_-hyperfine we tried to float an additional distortion constant, *D*_*K*_, but the rms deviations only decrease to 19.5 kHz and 17 kHz for the 4-MT···H_2_^16^O and for 5-MT···H_2_^16^O complexes respectively, instead of 20.5 kHz and 17.7 kHz without floating *D*_*K*_. Since the rms deviations of the BELGI-Cs fits are of the same magnitude as the XIAM fits and the fits give rise to similar results in the parameters, we present only the BELGI-C_s_ fits for the main isotopologues.

All XIAM and BELGI results are displayed in Table 1, S8–S10. Lists of fitted *A*- and *E*-species transitions for all isotopologues are presented in Table S11–S20 of the Supporting Information. When determined by XIAM and BELGI-C_s_-hyperfine respectively, the *V*_3_ determined for 4-MT···H_2_^16^O are 340.05(56) cm^–1^ and 329(5) cm^–1^; those for 5-MT···H_2_^16^O are 325.16(38) cm^–1^ and 329(4) cm^–1^. These compare with published results for the 4-methylthiazole and 5-methylthiazole monomers of 357.6(1) cm^–1^ and 332.0(8) cm^–1^ respectively (determined using the internal axis method). The results and conclusions of this work are insensitive to small variations in the assumed values of *F* and *F*_0_. For example, if it were assumed that *F*_0_ = 159.21 GHz (rather than 160.0 GHz) in the XIAM fit for 4-MT···H_2_O, this leads to a value of *V*_3_ of 338.53(56) cm^–1^ which is only slightly (1.5 cm^–1^) lower than the result shown in Table 1. The *r*_0_ geometries (determined as described below) for each of 4-MT···H_2_O and 5-MT···H_2_O have ∠(*i*,*b*) of 46.7(33)° and 70.9(18)° respectively. The XIAM and BELGI-C_s_-hyperfine fits respectively yield results for ∠(*i*,*b*) of 45.38(39)° and 43.6(10)° for 4-MT···H_2_^16^O; and results of 71.66(71)° and 72.10(51)° for 5-MT···H_2_^16^O. The values of ∠(*i*,*b*) and *V*_3_ for each complex vary slightly from one isotopologue to another, as expected, on account of statistical variance, changes in zero-point effects on isotopic substitution and slight changes in the orientation of inertial axes on isotopic substitution. The overall level of agreement is satisfactory, given the approximations involved in the determination of the *r*_0_ geometries and the uncertainties in the various determined parameters.

**Table 1:** Results of XIAM and BELGI-C_s_-Hyperfine Fits (using Watson’s *S* Reduction) of Spectroscopic Parameters to the Frequencies of *A*- and *E*-Species Transitions of the Main Isotopologues^a^

	4-MT···H_2_^16^O		5-MT···H_2_^16^O	
	XIAM^b^	BELGI^c^	XIAM^b^	BELGI^c^
*A*_0_ (MHz)	3863.381(53)^d^	3863(21)	4857.887(94)^d^	4857.9(16)
*B*_0_ (MHz)	1262.9188(25)	1263(21)	1085.7196(10)	1085.7(16)
*C*_0_ (MHz)	958.3805(22)	958.37546(84)	892.5126(11)	892.5059(11)
*D*_*J*_ (kHz)	0.2430(97)	–	0.1835(57)	–
*D*_*JK*_ (kHz)	2.123(84)	–	12.051(37)	–
*d*_1_ (kHz)	–0.0604(97)	–	–	–
χ_aa_(N3) (MHz)	–3.252(19)	–3.248(42)	–2.949(37)	–2.959(53)
[*χ*_*bb*_(N3) – *χ*_*cc*_(N3)] (MHz)	[−1.30]^e^	–1.165(97)	[−1.17]^e^	–1.18(15)
*F*_0_ (GHz)	[160.0]	[160.0]	[160.0]	[160.0]
*V*_3_ (cm^–1^)	340.05(56)	329(5)	325.16(38)	329(4)
∠(*i*,*b*) (deg)	45.38(39)	43.6(10)	71.66(71)	72.10(51)
Δ_0_ (u Å^2^)	–3.6540(23)	–3.670(41)	–3.2681(22)	–3.2612(16)
σ_RMS_ (kHz)	21.9	20.5	20.5	17.7
*N*_A_*/N*_E_	82/54	82/54	68/53	68/53

a The values of *F*_0_ are fixed to 160 GHz (see text for justification). *N*_A_ and *N*_E_ denote the number of *A*-species and *E*-species transitions respectively included in the fit.

b Values in the principal axis system.

c Values after transformation into the principal axis system. Detailed results of BELGI-Cs-hyperfine fits in the Rho Axis System (including centrifugal distortion constants) are provided in Table S10. Centrifugal distortion constants determined by BELGI-Cs cannot be directly compared with those determined by XIAM because of the different models employed.

d The results of the ωB97X-D/aug-cc-pVQZ calculation are *A*_*e*_ = 3681.328 MHz, *B*_*e*_ = 1300.843 MHz, and *C*_*e*_ = 972.818 MHz for 4-MT···H_2_O and *A*_*e*_ = 4532.340 MHz, *B*_*e*_ = 1091.668 MHz, and *C*_*e*_ = 887.458 MHz for 5-MT···H_2_O.

e [*χ*_*bb*_(N3) – *χ*_*cc*_(N3)] held fixed to the value determined by the *A*-species only (*effective*) fit for 4-MT···H_2_^16^O or 5-MT···H_2_^16^O as appropriate (see Tables S5 and S6).

### Molecular Geometry

It will be shown that 4-MT···H_2_O and 5-MT···H_2_O adopt the connectivities shown in Figure 1 which were prepared from the results of calculations at the ωB97X-D/aug-cc-pVQZ level. To distinguish between the two hydrogen atoms of the water subunit in each complex, from here forward, the hydrogen atom that participates in the intermolecular bond is denoted as H_b_ and the nonbonded hydrogen atom is denoted as H_nb_. Comparing the *r*_s_ coordinates (derived from the experimental results as described below) with the calculated *r*_e_ coordinates for H_b_ and H_nb_, it proved straightforward to reliably assign each isotopologue to its correct spectrum. The convention applied hereafter is that the isotopologue labeled “DOH” has D in the H_b_ position while the isotopologue labeled “HOD” has D in the H_nb_ position. For all calculations and fits of geometrical parameters, the rotational constants determined by the (XIAM) global fits of *A*- and *E*-species transitions were used.

Planar moments (*P*_*aa*_, *P*_*bb*_, and *P*_*cc*_) were calculated for each isotopologue of 4-MT···H_2_O and 5-MT···H_2_O as follows;
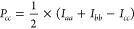
The results are shown in Table S21. For each of the complexes studied, the *P*_*cc*_ for isotopologues that contain H_2_^18^O or DOH are very similar to those of the parent isotopologues confirming that both H_b_ and the oxygen atom lie within the *ab* plane. For each isotopologue that contains HOD or D_2_O, the magnitude of *P*_*cc*_ is found to be slightly greater than that of the parent isotopologue. The inertial defect, Δ_0_, of each complex can be calculated from the moments of inertia about the *a*, *b* and *c* inertial axes using the following equation:
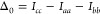
Inertial defects provide insight into the molecular geometry of a molecule or complex. In the vibrational ground state, the inertial defect is expected to be nonzero and a small positive result is expected where a molecule is planar. In-plane vibrations make positive contributions to Δ_0_ whereas negative contributions are expected from out-of-plane vibrations. The inertial defects of 4-methylthiazole^8^ and 5-methylthiazole^9^ monomers were calculated to be −3.091994(33) and −3.0769(7) u Å^2^, respectively while the same parameter was reported^4^ to be 0.0744(4) u Å^2^ for thiazole. The values of Δ_0_ for 4-MT···H_2_^16^O and 5-MT··· H_2_^16^O are calculated to be −3.6540(23) and −3.2681(22) u Å^2^, respectively. Evidently, the attachment of H_2_O to each of 4-methylthiazole and 5-methylthiazole adds a very small amount of out-of-plane mass such that Δ_0_ becomes more negative. The value of Δ_0_ becomes even more negative on substitution of a deuterium atom into the H_nb_ position of each complex. From the planar moments and inertial defects, therefore, it can be concluded that H_nb_ contributes mass outside of the *ab* plane even while H_b_ and O lie within (or close to) this plane for each complex. Previous studies have identified that H_nb_ undergoes rapid zero-point vibrational motion in complexes formed between a heteroaromatic ring and H_2_O. These motions rapidly interchange H_nb_ between equivalent positions on either side of the plane of the heteroaromatic ring. Hence, in the zero-point state, H_nb_ contributes additional out-of-plane mass even while the complex has an average geometry in which H_nb_ lies in the plane of the heavy atoms of the thiazole ring.

Substitution (*r*_s_) coordinates of the H and O atoms of the water subunit were determined from the measured shifts in rotational constants on isotopic substitution using the Kraitchman method implemented in the program, KRA.^46^ The coordinates (and their Costain errors^47^) presented in Table 2 were calculated from the results of the global fits presented in Tables 1, S8, and S9. The method calculates the magnitudes of coordinates but not their signs, so the latter were chosen to be consistent with the results of the ωB97X-D/aug-cc-pVQZ calculations. For both 4-MT···H_2_O and 5-MT···H_2_O, imaginary or very small coordinates were obtained for the *c*-coordinates of H_b_ and O confirming that these atoms lie within or close to the *ab* plane in each complex. The *r*_s_ coordinates determined for each complex are in good agreement with the *r*_e_ results calculated at the ωB97X-D/aug-cc-pVQZ level except for minor differences between the coordinates calculated for H_nb_ by each method (particularly the *c*-coordinate). This reflects that the calculations are for the equilibrium (*r*_e_) geometry, whereas the microwave spectrum is measured for the zero-point vibrational state of each complex. Precise agreement is therefore not expected between the calculated values of *r*_e_ coordinates and experimentally derived *r*_s_ and *r*_0_ coordinates of H_nb_ (particularly the *c*-coordinate). Nevertheless, the results are sufficiently consistent to confirm the model geometry of Figure 1 and to proceed to the determination of the *r*_0_ parameters.

**Table 2:** Comparison of DFT Calculated and Experimentally Determined *r*_s_ Atomic Coordinates of H_2_O in 4-MT···H_2_O and 5-MT···H_2_O

4-MT···H_2_O				
	Method	*a*/Å	*b*/Å	*c*/Å
H_b_	*r*_e_(calc.)	2.426683^a^	0.825440	–0.006987
	*r*_s_(exp.)	2.33263(77)^b^	0.9330(19)	[0]^c^
O	*r*_e_(calc.)	3.381957	0.878949	–0.162564
	*r*_s_(exp.)	3.42010(46)	0.7971(20)	[0]
H_nb_	*r*_e_(calc.)	3.754232	1.191681	0.659280
	*r*_s_(exp.)	3.78858(51)	1.3515(15)	0.3282(61)

5-MT···H_2_O				
	Method	*a*/Å	*b*/Å	*c*/Å
H_b_	*r*_e_(calc.)	2.944962	0.431497	0.028953
	*r*_s_(exp.)	2.89860(58)	0.7489(23)	0.046(38)
O	*r*_e_(calc.)	3.903074	0.315986	0.115239
	*r*_s_(exp.)	3.85045(41)	0.1796(87)	[0]
H_nb_	*r*_e_(calc.)	4.269998	0.647495	–0.701634
	*r*_s_(exp.)	4.34578(51)	0.6656(34)	–0.4576(50)

a *r*_e_ geometries calculated at the ωB97X-D/aug-cc-pVQZ level of theory.

b Numbers in parentheses are Costain errors for *r*_s_ results and one standard deviation in units of the last significant figure for *r*_0_ results.

c Imaginary values obtained for *r*_s_ coordinates are indicated in square brackets and assumed to be zero.

For each complex, the *r*_0_ method^48^ (as implemented in the STRFIT program made available on the PROSPE Web site^46^) was used to determine intermolecular bond lengths and angles. In each case, it was first assumed that geometrical parameters that are internal to the methylthiazole monomer are equal to their values in the calculated *r*_e_ geometry of the complex. The values of dihedral angles (∠(C2–N3···H_b_–O) and ∠(H_nb_–O–H_b_···N3)) were then chosen to fix the position of each of H_b_ and O to be within the plane of the ring. The H_nb_ atom of each complex is expected to undergo rapid zero-point vibrations between equivalent positions on either side of the plane of the ring. Different assumptions for the ∠(C2–N3···H_b_–O) and ∠(H_nb_–O–H_b_···N3) dihedral angles were tested as described below. Three coordinates were fitted to provide insight into the position and orientation of the water molecule. These include the intermolecular bond distance *r*(H_b_···N3), and two intermolecular bond angles, ∠ (H_b_···N3–C2) and ∠(O–H_b_···N3). The described analysis leads to definitive results for 5-MT···H_2_O but some ambiguity with regard to the geometry of 4-MT···H_2_O.

Close to the H_2_O binding site of 5-MT···H_2_O, the geometrical arrangement of atoms is known (confirmed by the values of *r*_s_ coordinates) to be very similar to that found in each of thiazole···H_2_O,^25^ imidazole···H_2_O^26^ and *N*-methylimidazole···H_2_O.^27^ Consistent with assumptions made during these earlier works, the assumptions that ∠(O–H_b_···N3–C2) = 0° and ∠(H_nb_–O–H_b_···N3) = 180° in the fit leads to *r*(H_b_···N3) = 2.0037(42) Å, ∠(H_b_···N3–C2) = 99.40(78)° and ∠(O–H_b_···N3) = 166.0(23)°. These are in good agreement with the calculated (ωB97X-D/aug-cc-pVQZ) values of geometrical parameters and lead to atomic coordinates that are consistent with the *r*_s_ coordinates of Table 2. The parameters cannot be fitted (the fit does not converge) under the alternative assumption that ∠(O–H_b_···N3–C2) = 180°. The situation is somewhat different for 4-MT···H_2_O. It is possible to fit the *r*(H_b_···N3), ∠(H_b_···N3–C2), or ∠(O–H_b_···N3) geometrical parameters (and obtain low uncertainties in parameter values) while assuming either that ∠(O–H_b_···N3–C2) is equal to 0° (fit 1, Table 3) or that this parameter is equal to 180° (fit 2, Table 3). Each of these alternative assumptions yields results for the fitted geometrical parameters that are very similar. Atomic coordinates calculated as described are presented in Tables S22–S25 of the Supporting Information. The experimentally determined values of nuclear quadrupole coupling constants (e.g., of the N atom) represent projections of the nuclear quadrupole coupling tensor onto inertial axes within the complex. Each of the two alternative geometries presented for 4-MT···H_2_O has a very similar orientation of its inertial axis framework. At the precision of the experiments, therefore, the experimentally determined nuclear quadrupole coupling constants do not distinguish between the two alternative molecular geometries. Hence, fits 1 and 2 are equally acceptable solutions for the molecular geometry of 4-MT···H_2_O from the perspective of the analysis of the experimental data of the present work.

**Table 3:** Comparison of the DFT Calculated and Experimentally Determined Structural Parameters

	4-MT···H_2_O	
Parameter	Method	Value
*r*(H_b_···N3)/Å	*r*_e_(calc.)	1.95708^a^
	*r*_0_: fit 1 (exp.)^b^	2.0265(87)^c^
	*r*_0_: fit 2 (exp.)^b^	2.0296(68)
∠(H_b_···N3–C2)/deg	*r*_e_(calc.)	130.89
	*r*_0_: fit 1 (exp.)	139.2(22)
	*r*_0_: fit 2 (exp.)	134.7(14)
∠(O–H_b_···N3)/deg	*r*_e_(calc.)	170.90
	*r*_0_: fit 1 (exp.)	169.3(71)
	*r*_0_: fit 2 (exp.)	167.4(43)

	5-MT···H_2_O	
Parameter	Method	Value
*r*(H_b_···N3)/Å	*r*_e_(calc.)	1.95966
	*r*_0_(exp.)	2.0037(42)
∠(H_b_···N3–C2)/deg	*r*_e_(calc.)	112.01
	*r*_0_(exp.)	99.40(78)
∠(O–H_b_···N3)/deg	*r*_e_(calc.)	168.35
	*r*_0_(exp.)	166.0(23)

a *r*_e_ geometries calculated at the ωB97X-D/aug-cc-pVQZ level of theory.

b Fit 1 assumes ∠(O–H_b_···N3–C2) = 0° and ∠(H_nb_–O–H_b_···N3) = 180°. Fit 2 assumes ∠(O–H_b_···N3–C2) = 180° and ∠(H_nb_–O–H_b_···N3) = 180°

c Numbers in parentheses are one standard deviation in units of the final significant figure

The ambiguity in the molecular geometry of 4-MT···H_2_O will be resolved in Section 5 with reference to a broader, emerging trend in the molecular geometries of methylthiazole···H_2_O and methylimidazole···H_2_O complexes. Values of fitted *r*_0_ parameters depend on assumptions regarding parameters that are internal to the heteroaromatic monomer and are especially sensitive to assumptions about the positions of heavy atoms, and isotopic substitutions were not performed for atoms within the 4-methylthiazole subunit. Principally for these reasons, the true uncertainties in the *r*_0_ parameters of Tables 3 and 4 can be expected to be greater than are implied by the standard deviations quoted. However, it is satisfying to note that the *r*_s_ and *r*_0_ (fit 2) coordinates of H_b_ and O are highly consistent (Table S26).

**Table 4:** Comparison of Experimentally Determined (*r*_0_) Structural Parameters for Complexes Formed between 5-Membered *N*-Heterocyclic Rings and H_2_O

Complex^a^	H-bond donor on het. ring^b^	*r*(H_b_···N3)/Å	∠(H_b_···N3–C2)/deg	∠(O–H_b_···N3)/deg
imidazole···H_2_O	C–H on C2	1.927(27)^c^	99.9(41)	172.1(26)
*N*-methylimidazole···H_2_O	C–H on C2	1.922(4)	101.0(16)	177(5)
thiazole···H_2_O	C–H on C2	1.977(7)	95.6(4)	168.9(1)
5-MT···H_2_O	C–H on C2	2.0037(42)	99.40(78)	166.0(23)
				
2-methylimidazole···H_2_O	C–CH_3_ on C2	1.923(5)	116.9(9)	166.3(28)
4-MT···H_2_O	C–CH_3_ on C4	2.0296(68)^d^	134.7(14)^d^	167.4(44)^d^

a Results for imidazole···H_2_O, *N*- and 2-methylimidazole···H_2_O complexes from refs (26 and 27) respectively. Results for thiazole···H_2_O following reanalysis of results presented in ref (25) (see Table S27).

b Indicates the atom or group of the heteroaromatic subunit which acts as the hydrogen bond donor in the secondary (weaker) hydrogen bond within the complex.

c Uncertainties in parentheses are those quoted in the primary source.

d Results of fit 2 for 4-MT···H_2_O (see Table 3).

### Noncovalent Interactions

**Figure 3:**
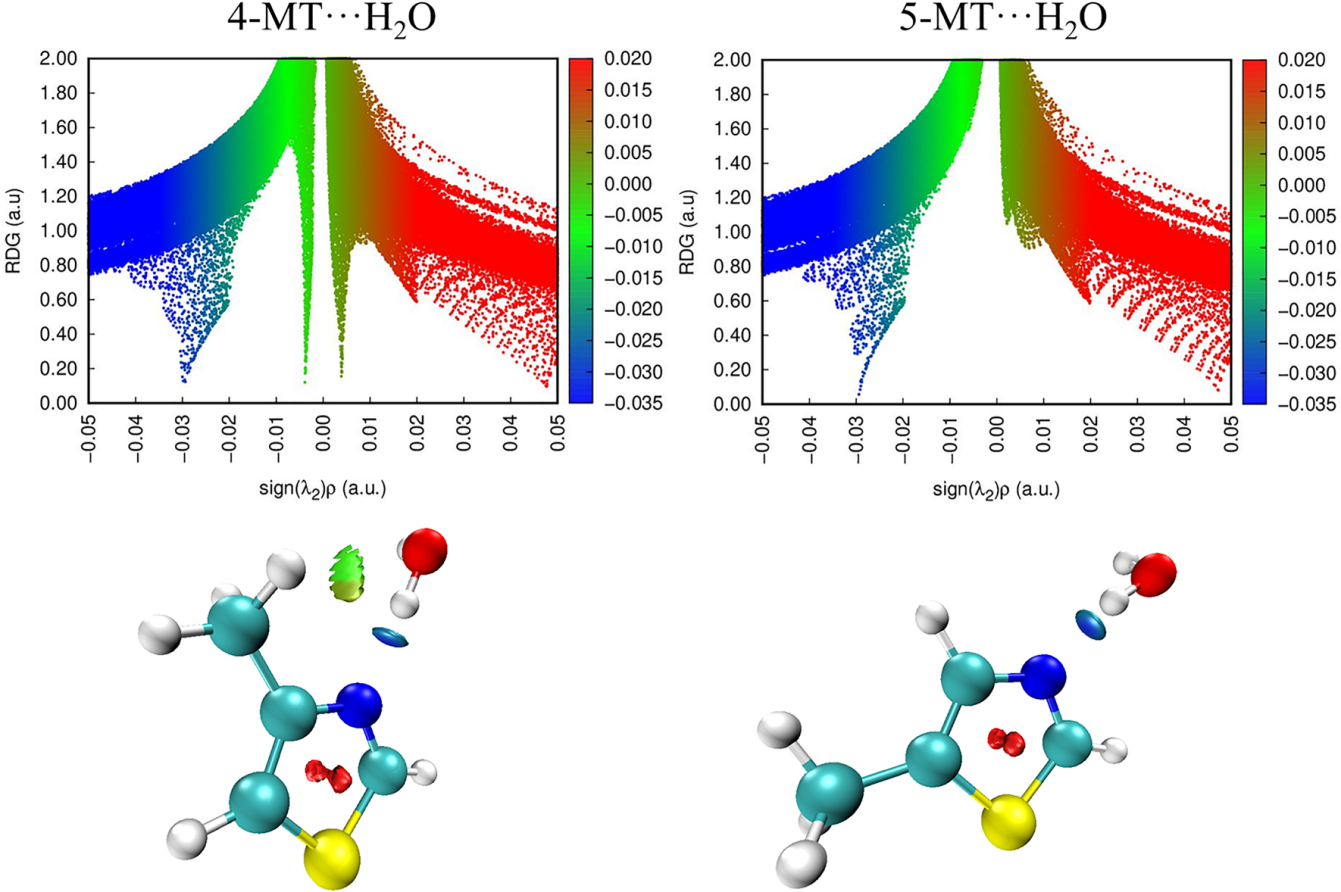
NCI isosurfaces and plots of the RDG (a.u.) vs sign(λ_2_)ρ of 4-MT···H_2_O and 5-MT···H_2_O. Positive and negative values of sign(λ_2_)ρ respectively denote repulsive (red) and attractive (blue) interactions. The isosurface *s* value is 0.5 au.

Noncovalent interaction (NCI)^49^ and natural bond orbital (NBO)^50^ analyses were performed to provide insights into the intermolecular interactions present within 4-MT···H_2_O and 5-MT···H_2_O. The analysis of molecular geometry described above showed that the water molecule binds to the nitrogen atom of the thiazole ring via a hydrogen bond which is nonlinear in each complex. It was further suggested that each complex is stabilized by a secondary interaction involving the O atom of H_2_O. NCI plots of the reduced density gradient, RDG, versus the sign of the second eigenvalue of the Hessian matrix (λ_2_) of the electronic density (ρ), sign(λ_2_)ρ, were generated using the program Multiwfn^51^ and are displayed in Figure 3. These plots were prepared for the geometries optimized at the ωB97X-D/aug-cc-pVQZ level of theory. For both 4-MT···H_2_O and 5-MT···H_2_O, a dark blue disk between the nitrogen atom of the thiazole ring and the hydrogen atom of the water molecule indicates a strong attractive (hydrogen bonding) interaction is present within both complexes. The plots indicate that the strength of the attractive interactions is approximately the same in both complexes. A second isosurface is also present within the 4-MT···H_2_O complex which shows an area of weak attraction (light green, −(λ_2_)ρ value) and an area of weak repulsion (dark green, +(λ_2_)ρ value). There are no isosurface disks that would imply the presence of secondary interactions within 5-MT···H_2_O which is not consistent with the experimental result that ∠(O–H_b_···N3) of 5-MT···H_2_O deviates from linearity (∠(O–H_b_···N3) = 166.0(23)° as shown in Tables 3 and 4). This minor inconsistency presumably arises because an interaction is present but extremely weak. It should be noted that a previous study which also employed both microwave spectroscopy experiments and NCI analyses did not find evidence of a secondary interaction in thiazole···H_2_O.^25^ During the present work, the molecular geometry of thiazole···H_2_O was reanalyzed (using the results presented within the original study) and it was thereby determined that ∠(O–H_b_···N3) = 168.9(1)° for thiazole···H_2_O (see Table 4 and further details in Table S27). The implication is that the geometry of the noncovalent interactions present within 5-MT···H_2_O is very similar to that within thiazole···H_2_O as expected.

**Figure 4:**
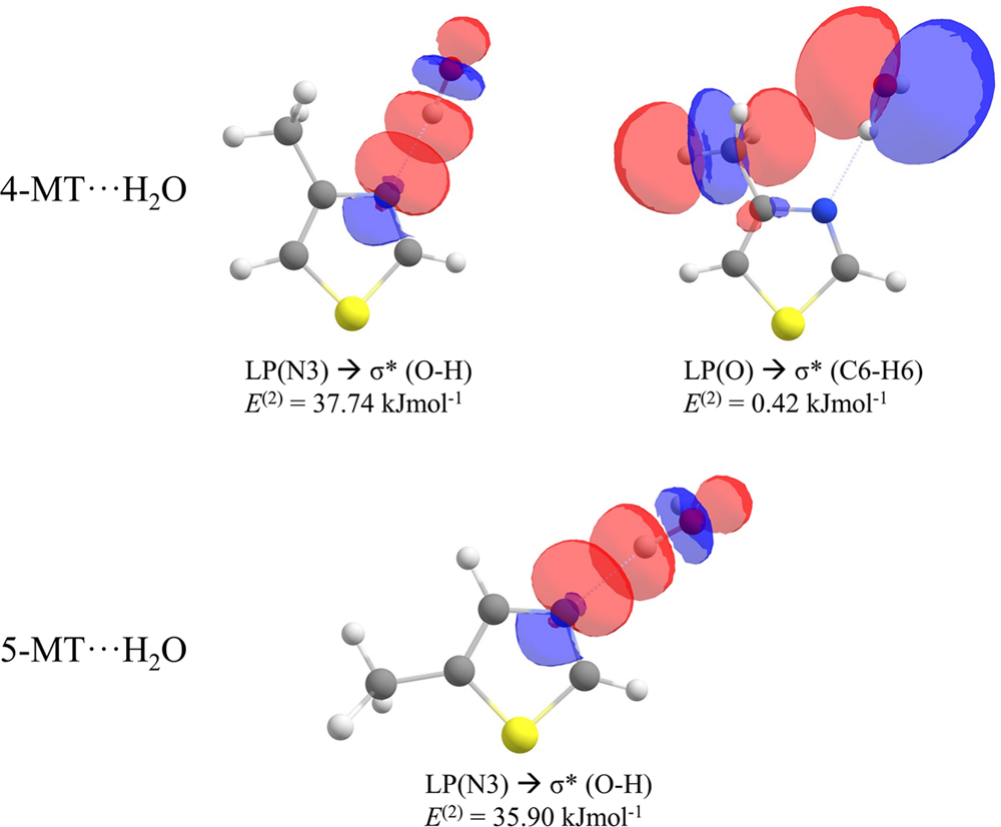
NBO plots associated with intermolecular interactions present within 4-MT···H_2_O (upper row) and 5-MT···H_2_O (lower row). *E*^(2)^ represents the second order perturbation energy of each interaction.

NBO analysis was performed at the same level of theory as described above to provide information about the intermolecular orbital interactions present within this complex and the second-order stabilization energies (*E*^(2)^) associated with the interactions. The results of the NBO analysis are presented in Figure 4 and Table S28. In both complexes, the largest *E*^(2)^ contribution corresponds to the interaction between the lone pair of the nitrogen atom and the antibonding σ*(H_b_–O) orbital. In 4-MT···H_2_O and 5-MT···H_2_O, the second order stabilization energies are 37.74 and 35.90 kJ mol^–1^ respectively and therefore consistent with the result reported previously for the thiazole···H_2_O complex (*E*^(2)^ = 35.60 kJ mol^–1^). The NCI analysis reveals an additional weak interaction within 4-MT···H_2_O between the oxygen atom of the water molecule and the hydrogen atoms of the methyl group. The same interaction was identified in the NBO analysis, which shows the lone pair of the oxygen atom interacting with a σ*(C–H) antibonding orbital on the methyl group. This interaction is calculated to have a second-order stabilization energy of 0.42 kJ mol^–1^ which is apparently strong enough to cause the primary hydrogen bond (between H_b_ and N) to deviate from linearity. The effect of this interaction on the *V*_3_ barrier to internal rotation of the methyl group is discussed further below.

## 5 Discussion

Section 4 (under Molecular Geometry) explained that it is not possible to distinguish between two alternative geometries of 4-MT···H_2_O from the experimental data of the present work alone. Changing the ∠(O–H_b_···N3–C2) dihedral angle from 0° to 180° while fixing all other parameters very nearly transforms the first alternative geometry into the other. It will be shown that only one of the alternative geometries allowed by the experimental data for 4-MT···H_2_O is consistent with a clear trend emerging from studies of related complexes and rationalized by the noncovalent interactions present.

Thiazole and imidazole are both 5-membered rings that contain heteroatoms in the 1 and 3 positions. Geometrical parameters are available for six monohydrated complexes of heteroaromatics where H_2_O acts as a hydrogen bond donor while the pyridinic nitrogen of thiazole or imidazole acts as a hydrogen bond acceptor (Table 4) in each case. The length of the primary hydrogen bond in imidazole···H_2_O is shorter than that in thiazole···H_2_O by 0.050(28) Å. This reflects a general trend whereby the length of the primary hydrogen bond in thiazole-containing monohydrate complexes is slightly shorter than that in those that contain imidazole. Imidazole···H_2_O,^26^ thiazole···H_2_O,^25^*N*-methylimidazole···H_2_O,^27^ and 5-MT···H_2_O represent an important subset of the complexes shown in Table 4. Each of these complexes has been shown to bind H_2_O via a strong hydrogen bonding interaction at the pyridinic nitrogen atom and a weaker hydrogen-bonding interaction between the O atom of H_2_O and a hydrogen attached directly to C2 of the heteroaromatic. The *r*_0_ results of Table 3 imply that the length of this weaker (O···H) hydrogen bond is 2.953(4) Å for 5-MT···H_2_O. The CH_3_ group of each of *N*-methylimidazole···H_2_O and 5-MT···H_2_O is remote (on the opposite side of the ring) from the H_2_O subunit and therefore not positioned to interact with it through hydrogen bonding. Each complex of the subset identified above thus has a similar balance of electrostatic forces at the H_2_O binding site which leads to each complex having a very similar value of each of ∠(H_b_···N3–C2) and ∠(O–H_b_···N3). On the other hand, the value of ∠(H_b_···N3–C2) for 2-methylimidazole···H_2_O^27^ is somewhat (∼16°) greater than found for imidazole···H_2_O, thiazole···H_2_O, *N*-methylimidazole···H_2_O and 5-MT···H_2_O. This difference is a consequence of the position of the CH_3_ group on C2 of the heteroaromatic in 2-methylimidazole···H_2_O which allows for a weak hydrogen bond between O of H_2_O and the CH_3_ group (rather than an individual hydrogen atom attached to C2, as was the case in the other complexes identified above). Hence, the values of geometrical parameters for the five complexes mentioned above can be rationalized with the noncovalent interactions present. This is a useful perspective from which to re-examine the alternative possibilities for the geometry of 4-MT···H_2_O allowed by the experimental data.

Each of the two alternatives for the geometry of 4-MT···H_2_O would imply a significantly greater value of ∠(H_b_···N3–C2) than that of any of the other complexes of Table 4. The various results for ∠(H_b_···N3–C2) presented in Table 4 can be interpreted as an indication of the noncovalent interactions present in these complexes and imply that the balance of noncovalent interactions at the H_2_O binding site of 4-MT···H_2_O is somewhat different from that which governs the geometries of the other complexes. The simplest physical rationalization of the results for 4-MT···H_2_O and the overall trend, therefore, is to invoke a weak interaction between the O of H_2_O and the CH_3_ group attached to C4 of 4-MT which would imply that ∠(O–H_b_···N3–C2) should be assumed equal to 180°, in the zero-point geometry, with the lone pair on the oxygen oriented toward the CH_3_ group. The ambiguity in the molecular geometry of 4-MT···H_2_O is thus resolved in favor of the model summarized by the results of fit 2. In this molecular geometry, two hydrogens of the CH_3_ group are oriented such that ∠(N3–C4–C6–H) = ±60° and the distance between oxygen and each of these hydrogens is 3.118(4) Å. An internal rotation of CH_3_ (an adjustment of ∠(N3–C4–C6–H)) by ±60° from this geometry would locate a single hydrogen atom of CH_3_ in the plane of the heavy atoms of the complex and only 2.500(4) Å from the oxygen atom. The results for ∠(O–H_b_···N3) are similar across the entire series of complexes suggesting that this parameter is less sensitive to the detailed balance of electrostatic forces present than is ∠(H_b_···N3–C2). In each complex featured in Table 4, the primary hydrogen bond deviates slightly from linearity because of the secondary interaction between O of the H_2_O subunit and the neighboring H atom or CH_3_ attached to C2/C4 of the heteroaromatic.

The optimized (*r*_e_) geometry of 4-MT···H_2_O has ∠(O–H_b_···N3–C2) = 141.7° and ∠(H_nb_–O–H_b_···N3) = 140.1°. The former of these angles is significantly closer to 180° than to 0° in support of the aforementioned conclusion that fit 2 leads to the more accurate description of the experimentally determined molecular geometry. However, the experiment probes the zero-point state of the complex, while the calculation is of the equilibrium (*r*_e_) geometry. The DFT-calculated geometry of 5-MT···H_2_O has ∠(O–H_b_···N3–C2) = −6.9° and ∠(H_nb_–O–H_b_···N3) = 122.7°. This is again consistent with the assumptions made when performing fits to determine geometrical parameters of this complex (∠(O–H_b_···N3–C2) = 0° and ∠(H_nb_–O–H_b_···N3) = 180°) and with the rationale used to explain the overall trend in the molecular geometries discussed above.

A previous study^27^ reported an interesting trend in the (*V*_3_) barriers to internal rotation of the CH_3_ group in *N*-methylimidazole, 2-methylimidazole, and each of their complexes with H_2_O. The *N*-methylimidazole···H_2_O complex has *V*_3_ of 182.21(12) cm^–1^ which is very similar to the value of 185.104(11) cm^–1^ recorded for the *N*-methylimidazole monomer where the comparison was between the results of fits using XIAM. This is consistent with the CH_3_ group being remote from and not interacting with the H_2_O molecule in the geometry of the *N*-methylimidazole···H_2_O complex. The 2-methylimidazole···H_2_O complex was found to have *V*_3_ of 154.99(8) cm^–1^ which is somewhat greater than the *V*_3_ of the 2-methylimidazole monomer which was determined to be 122.7529(38) cm^–1^ (where the results quoted were obtained using XIAM). The difference was shown to be significantly greater than the dependency of *V*_3_ on the specific Hamiltonian employed, fits performed using BELGI-C_s_ yielded results for *V*_3_ of 173.6(16) cm^–1^ and 150.68(90) cm^–1^ for *N*-methylimidazole···H_2_O and 2-methylimidazole···H_2_O respectively which are each within 5% of the comparable XIAM results. The difference between the *V*_3_ values determined for 2-methylimidazole and for 2-methylimidazole···H_2_O was interpreted to be a result of the presence of a weak electrostatic (through-space, hydrogen-bonding) interaction between H_2_O and the CH_3_ group of the complex.

The splitting between an *A* species transition and its *E* species counterpart increases with a decreasing (*V*_3_) barrier. It is therefore possible to experimentally measure low barriers with greater precision than higher barriers. The *V*_3_ for 4-MT···H_2_O and 5-MT···H_2_O are each significantly higher (and hence determined with lower precision) than those reported previously for *N*-methylimidazole···H_2_O and 2-methylimidazole···H_2_O. The result for *V*_3_ for the 5-methylthiazole monomer was reported as 332.0(8) cm^–1^ when analyzed by an internal axis method^9^ which compares with 325.16(38) cm^–1^ and 329(4) cm^–1^ for 5-MT···H_2_O when fitted during the present work using XIAM and BELGI respectively. The differences between the XIAM and BELGI results show that the determined value of *V*_3_ for 5-MT···H_2_O is dependent on the Hamiltonian model used in the analysis. It will also depend somewhat on the data set (i.e., the range of spectroscopic transitions that have been measured) and the value assumed for *F*_0_ which could not be directly fitted and was assumed to be equal to the value for the 5-methylthiazole monomer. Hence, the small difference between the values of *V*_3_ determined previously for 5-methylthiazole and herein for 5-MT···H_2_O may arise because of limitations of the models employed and the narrow range of rotational transitions measured (with data obtained for σ = 0 and σ = ± 1 torsional levels of the *v*_*t*_ = 0 state only); and may not necessarily attribute to fundamental molecular physical factors. The results for the 4-MT···H_2_O complex invite a more detailed interpretation. The present work determined values of *V*_3_ of 340.05(56) cm^–1^ and 329(5) cm^–1^ for 4-MT···H_2_O when fits were performed using XIAM and BELGI respectively. The same parameter was determined to be 357.6(1) cm^–1^ for the 4-methylthiazole monomer.^8^ The difference between the *V*_3_ barriers determined for 4-MT···H_2_O and 4-methylthiazole, with the result for *V*_3_ being slightly *lower* for the former than that for the latter, initially seems anomalous. It was previously noted that *V*_3_ for 2-methylimidazole···H_2_O is *higher* than for 2-methylimidazole and this difference was attributed to the presence of a weak hydrogen bond between the oxygen atom of H_2_O and the CH_3_ group in the 2-methylimidazole···H_2_O complex. However, there are effects that might explain the results for 4-MT···H_2_O even though this complex also contains a weak hydrogen bond similar to that found in 2-methylimidazole···H_2_O.

Ouyang et al. reported analyses of the rotational spectra of mono- and dihydrates of acetic acid in 2008.^52^ In each of these complexes, the water molecule(s) coordinates to the carboxyl group of the acid on the opposite side of the molecule from the CH_3_ group. The water molecule within CH_3_COOH···H_2_O simultaneously acts as both a hydrogen bond donor (to the carboxyl oxygen atom) and a hydrogen bond acceptor (from the carboxyl O–H). A redistribution of electrons within the carboxyl group (a consequence of the coordination of the H_2_O molecule) leads to a significantly *reduced V*_3_ barrier of the CH_3_ group relative to that in the acetic acid monomer. The CH_3_COOH···(H_2_O)_2_ complex was shown to contain three hydrogen bonds which cooperatively reinforce each other such that there is a further decrease in the *V*_3_ barrier of CH_3_ relative to that for the unhydrated acetic acid monomer. Hence, the *V*_3_ barriers of CH_3_COOH, CH_3_COOH···(H_2_O) and CH_3_COOH···(H_2_O)_2_ are 168.16(10), 138.396(5), and 118.482(2) cm^–1^ respectively and the differences between these arise because of changes in electronic charge distribution within the acetic acid subunit. This benchmark study and other works^53,54^ show that the *V*_3_ barrier at a CH_3_ group can be significantly reduced by charge redistribution within a molecule, and that such redistribution might be caused by hydrogen bonding between one molecule and another. A more recent example of the same effect (but caused by a homochalcogen bond rather than a hydrogen bond) was described by Obenchain et al. in 2018. The *V*_3_ barrier in the dimethylsulfide monomer is 735.784(44) cm^–1^ whereas it is 656.1(14) cm^–1^ in the dimethylsulfide···SO_2_ complex.^55^

The observed reduction in *V*_3_ implies that charge redistribution occurs within the 4-methylthiazole monomer on the attachment of H_2_O leading to a measurable effect (a slight reduction) of the *V*_3_ barrier to internal rotation of the CH_3_ group. However, as described in Section 4 (Molecular Geometry), the experimentally determined molecular geometry of 4-MT···H_2_O implies that a weak hydrogen bond is present between the H_2_O subunit and CH_3_, and (from the evidence of previous work) the presence of this bond will tend to increase the value of *V*_3_ (relative to *V*_3_ for the isolated 4-methylthiazole monomer). Evidently, the effects of through-space (hydrogen bonding and other electrostatic) interactions and through-bond contributions (which arise because of charge redistribution within a molecular subunit) to *V*_3_ may be convoluted for this complex, may counteract each other, and cannot be independently distinguished through analysis of the experimental data alone. It should be noted that this will also be true of through-space and through-bond contributions to *V*_3_ for the 2-methylimidazole···H_2_O complex reported previously. In that case, it was not necessary to invoke a through-bond contribution to explain the determined value of *V*_3_ for 2-methylimidazole···H_2_O but it was also not possible to exclude that such an effect might be present. The value of *V*_3_ for 2-methylimidazole···H_2_O is significantly higher than that for the 2-methylimidazole monomer. A through-bond contribution to *V*_3_ of 2-methylimidazole···H_2_O might act to reduce *V*_3_ (relative to the value for the isolated 2-methylimidazole monomer) even while the effect of the hydrogen bond between the O atom of H_2_O and the CH_3_ group of 2-methylimidazole dominates and leads to the observed increase in the parameter.

The second-order stabilization energy of the interaction between the lone pair of the oxygen atom and the σ*(C–H) antibonding orbital on the methyl group of 2-methylimidazole···H_2_O is calculated herein to be 2.83 kJ mol^–1^ (at the B3LYP/aug-cc-pVTZ level of theory). The same interaction within 4-MT···H_2_O is calculated to be 0.42 kJ mol^–1^. This difference between the strengths of the secondary interactions in 2-methylimidazole···H_2_O and in 4-MT···H_2_O partly explains the observed values of *V*_3_ for these complexes. Evidently, the described hydrogen bond within 2-methylimidazole···H_2_O is stronger than that present within 4-MT···H_2_O and therefore has a more significant effect on *V*_3_. The effect of through-bond charge redistribution internal to the 4-MT subunit may be the dominant effect for 4-MT···H_2_O explaining why *V*_3_ is lower for this complex than it is for the 4-methylthiazole monomer. The *V*_3_ of each of *N*-methylimidazole···H_2_O and 5-MT···H_2_O have been found to be similar to those of the respective monomers, *N*-methylimidazole and 5-methylthiazole. For each of these complexes, the possibility that through-space and through-bond interactions cause large effects that are opposite and equal seems less likely than the alternative possibility that both through-space and through-bond contributions to *V*_3_ are negligible given that CH_3_ is remote from the coordinating H_2_O.

## 6 Conclusions

Broadband rotational spectra of five isotopologues of each of 4-MT···H_2_O and 5-MT···H_2_O have been recorded over the 7.0–18.5 GHz region. The (*V*_3_) barriers to the internal rotation of the CH_3_ group within each complex have been determined. Each complex contains a primary hydrogen bond between the nitrogen atom of the thiazole ring and an H atom of H_2_O. This bond deviates slightly from linearity because of a secondary, weak hydrogen bond between the O group and either the CH_3_ group attached to C4 (in 4-MT···H_2_O) or the hydrogen atom attached to C2 (in 5-MT···H_2_O). The barrier to internal rotation, *V*_3_, for the CH_3_ group in 4-MT···H_2_O is slightly lower than the published result for the 4-methylthiazole monomer which may be a result of internal charge redistribution within 4-methylthiazole following its coordination by H_2_O. At the precision with which the present experiments have been performed, *V*_3_ for 5-MT···H_2_O is unchanged from the result determined previously for the 5-methylthiazole monomer.

## Supporting Information Available

Tables S1–S3, atomic coordinates of optimized geometries of 4-MT···H_2_O and 5-MT···H_2_O; Table S4, comparison of calculated and experimentally determined parameters; Tables S5 and S6, spectroscopic constants determined for 4-MT···H_2_O and 5-MT···H_2_O using PGOPHER; Table S7, nuclear quadrupole coupling constants in the principle nuclear axis framework determined using QDIAG; Tables S8 and S9, spectroscopic parameters determined from global (XIAM) fits obtained by fitting to the frequencies of *A*- and *E*-species transitions for five isotopologues of 5-MT···H_2_O; Table S10, molecular parameters of 4-MT···H_2_^16^O and 5-MT···H_2_^16^O in the rho axis system (RAM) obtained using the BELGI-Cs-hyperfine code; Tables S11–S20, observed transition frequencies of *A*- and *E*-species transitions of isotopologues of 4-MT···H_2_O and 5-MT···H_2_O alongside ν_obs_ – ν_calc_ values obtained after fitting with the XIAM and BELGI-Cs hyperfine programs; Table S21, inertial defects and planar moments of 4-MT···H_2_O and 5-MT···H_2_O calculated from rotational constants determined from global (XIAM) fits; Table S22–S24, results of fits of structural parameters (4-MT···H_2_O, fit 1), (4-MT···H_2_O, fit 2) and 5-MT···H_2_O; Table S25, atomic coordinates of 4-MT···H_2_O and 5-MT···H_2_O determined by the *r*_0_ method; Table S26, comparison of DFT calculated and experimentally determined (*r*_s_ and *r*_0_) atomic coordinates of H_2_O in 4-MT···H_2_O and 5-MT···H_2_O; Table S27, results of a new fit of structural parameters of thiazole···H_2_O; Table S28, NBO stabilization energy contributions (≥0.21 kJ mol-1) for 4-MT···H_2_O and 5-MT···H_2_O calculated at the B3LYP(D3BJ)/aug-cc-pVTZ level of theory (PDF)
