# Supplementary material for: Noncovalent Interactions in the Molecular Geometries of 4-Methylthiazole···H2O and 5-Methylthiazole···H2O Revealed by Microwave Spectroscopy
Source: J Phys Chem A. 2023 Sep 26;127(39):8133–45. doi: 10.1021/acs.jpca.3c05360 (PMC10561259; doi:10.1021/acs.jpca.3c05360)
Supplement: Supplementary file 1 — jp3c05360_si_001.pdf [file jp3c05360_si_001.pdf]

Journal of Physical Chemistry A

Supplementary Information

Non-covalent Interactions in the Molecular Geometries of 4-Methylthiazole···H_2O and 5-Methylthiazole···H_2O Revealed by Microwave Spectroscopy.

Charlotte N. Cummings[1], Isabelle Kleiner[2] and Nicholas R. Walker[1,*]

1) Chemistry- School of Natural and Environmental Sciences, Newcastle University, Bedson Building, Newcastle-upon-Tyne, NE1 7RU, UK.
2) Université de Paris and Univ. Paris Est Creteil, CNRS, LISA, F-75013 Paris, France.

KEYWORDS: rotational spectroscopy, thiazole, microsolvation, chirped-pulse, microwave.

AUTHOR INFORMATION:

* Corresponding Author: Nicholas R. Walker, nick.walker@ncl.ac.uk

Table S1 - Atomic coordinates of the optimised geometries of 4-MT···H_2O and 5-MT···H_2O calculated at the ωB97X-D/aug-cc-pVQZ level of theory.

	4-MT···H_2O[a]		
	a /Å	*b* /Å	*c* /Å
S(1)	2.022138	−0.463750	−0.053039
C(2)	0.510681	−1.248050	0.110898
H(2)	0.435180	−2.322442	0.177219
N(3)	−0.510960	−0.454331	0.142843
C(4)	−0.129991	0.861572	0.034237
C(5)	1.210948	1.043290	−0.081379
H(5)	1.745598	1.971735	−0.180835
C(6)	−1.174024	1.926922	0.056372
H(6)	−1.689065	1.932148	1.016878
H(6)	−0.734364	2.907808	−0.108679
H(6)	−1.925286	1.737917	−0.709307
H_b	−2.426683	−0.825440	−0.006987
O	−3.381957	−0.878949	−0.162564
H_{nb}	−3.754232	−1.191681	0.659280
	5-MT···H_2O		
	a /Å	*b* /Å	*c* /Å
S(1)	−1.094843	−1.169022	0.016412
C(2)	0.606365	−0.960226	−0.049631
H(2)	1.284566	−1.799800	−0.058986
N(3)	0.994839	0.272890	−0.081158
C(4)	−0.078202	1.121547	−0.053273
H(4)	0.090801	2.187781	−0.072187
C(5)	−1.303401	0.541368	0.000442
C(6)	−2.640493	1.201188	0.051162
H(6)	−3.281370	0.877418	−0.768448
H(6)	−3.157671	0.986094	0.986003
H(6)	−2.515386	2.279697	−0.024839
H_b	2.944962	0.431497	0.028953
O	3.903074	0.315986	0.115239
H_{nb}	4.269998	0.647495	−0.701634

[a] The signs of coordinates of 4-MT···H_2O are as computed by the original calculation. The signs of *a* and *b* coordinates have been reversed in Table 2 of the paper and Table S26 of the supplementary information (the coordinate system has been rotated about the *c* axis by 180 degrees) for clarity of presentation.

Table S2 - Atomic coordinates of the optimised geometries of 4-MT···H_2O and 5-MT···H_2O calculated at the B3LYP(D3BJ)/aug-cc-pVTZ level of theory.

	4-MT···H_2O		
	a /Å	b /Å	c /Å
S(1)	2.048543	−0.434163	−0.015810
C(2)	0.531089	−1.261319	0.025755
H(2)	0.480429	−2.338631	0.037296
N(3)	−0.508232	−0.484435	0.038429
C(4)	−0.154065	0.849721	0.013730
C(5)	1.191200	1.064564	−0.016577
H(5)	1.713605	2.004575	−0.040494
C(6)	−1.223099	1.891378	0.018329
H(6)	−1.846435	1.795576	0.907643
H(6)	−0.794020	2.890831	−0.005945
H(6)	−1.880317	1.767554	−0.842726
H_b	−2.405256	−0.850513	0.001525
O	−3.378706	−0.851451	−0.069344
H_{nb}	−3.680910	−1.547101	0.519833
	5-MT···H_2O		
	a /Å	b /Å	c /Å
S(1)	−1.178570	−1.151347	0.016925
C(2)	0.551390	−1.056128	−0.038781
H(2)	1.165199	−1.942983	−0.044489
N(3)	1.020029	0.152767	−0.066915
C(4)	0.006543	1.079362	−0.044084
H(4)	0.251809	2.130448	−0.059217
C(5)	−1.261452	0.585962	0.000843
C(6)	−2.552383	1.332282	0.035127
H(6)	−3.180244	1.091810	−0.823820
H(6)	−3.124297	1.107134	0.936229
H(6)	−2.355975	2.403311	0.019719
H_b	2.937721	0.360068	0.011373
O	3.909011	0.348758	0.105097
H_{nb}	4.250549	0.600845	−0.756559

Table S3 - Atomic coordinates of the optimised geometries of 4-MT···H_2O and 5-MT···H_2O calculated at the MP2/aug-cc-pVDZ level of theory.

4-MT···H_2O			
	a /Å	b /Å	c /Å
S(1)	2.098264	−0.330444	−0.000175
C(2)	0.648656	−1.280138	0.000266
H(2)	0.688944	−2.369866	0.000440
N(3)	−0.476447	−0.577919	0.000418
C(4)	−0.219749	0.780812	0.000180
C(5)	1.133427	1.101161	−0.000191
H(5)	1.592007	2.089457	−0.000491
C(6)	−1.363501	1.756591	0.000186
H(6)	−1.998345	1.603714	0.886192
H(6)	−0.986843	2.789683	0.000997
H(6)	−1.997432	1.604830	−0.886677
H_b	−2.399050	−0.918171	0.000113
O	−3.370177	−0.798476	−0.000465
H_{nb}	−3.724637	−1.695848	0.001302
5-MT···H_2O			
	a /Å	b /Å	c /Å
S(1)	−0.953136	−1.211028	0.003870
C(2)	0.729757	−0.789703	−0.040356
H(2)	1.512100	−1.549810	−0.051566
N(3)	0.973697	0.515140	−0.053705
C(4)	−0.206124	1.223618	−0.027824
H(4)	−0.181334	2.315012	−0.032760
C(5)	−1.376631	0.474574	0.004669
C(6)	−2.798341	0.961845	0.038872
H(6)	−3.367963	0.607240	−0.834229
H(6)	−3.318454	0.625077	0.949147
H(6)	−2.802345	2.062247	0.028039
H_b	2.934277	0.568506	0.017325
O	3.849344	0.233857	0.093368
H_{nb}	4.315977	0.651425	−0.640987

Table S4 - Spectroscopic parameters of 4-MT···H_2O and 5-MT···H_2O calculated at different levels of theory.

	4-MT···H_2O					
	ωB97X-D/aug-cc-pVQZ		B3LYP(D3BJ) /aug-cc-pVTZ		MP2/aug-cc-pVDZ	
A_e (MHz)	3681.328	– 4.7 %[a]	3753.020	– 2.9 %	4031.812	+ 4.4 %
B_e (MHz)	1300.843	+ 3.0 %	1291.582	+ 2.3 %	1255.932	– 0.6 %
C_e (MHz)	972.818	+ 1.5 %	968.054	+ 1.0 %	963.409	+ 0.5 %
χ_{aa} (N3) (MHz)	–3.7393	+ 15.2 %	–3.5887	+ 10.5 %	–2.4257	– 25.3 %
[χ_{bb}(N3) – χ_{cc}(N3)] (MHz)	–1.5097	+ 16.1 %	–1.3805	+ 6.2 %	–1.4242	+ 9.6 %
V_3 (cm^{-1})[b]	287.173	– 15.2 %	288.398	– 14.8 %	309.154	– 8.7 %
|μ_a|, |μ_b|, |μ_c| (D)[c]	2.86, 0.75, 1.36		2.96, 0.17, 0.95		2.82, 0.19, 0.0	
	**5-MT···H_2O**					
	ωB97X-D/aug-cc-pVQZ		B3LYP(D3BJ) /aug-cc-pVTZ		MP2/aug-cc-pVDZ	
A_e (MHz)	4532.340	– 6.7 %	4318.523	–11.0 %	4740.083	– 2.4 %
B_e (MHz)	1091.668	+ 0.5 %	1096.919	+ 1.0 %	1085.845	0.0 %
C_e (MHz)	887.458	– 0.5 %	882.231	– 1.2 %	890.486	– 0.2 %
χ_{aa} (N3) (MHz)	–3.7701	+ 28.4 %	–3.8673	+ 31.7%	–2.9369	+ 0.03 %
[χ_{bb}(N3) – χ_{cc}(N3)] (MHz)	–1.8699	+ 59.8 %	–1.3865	+18.5 %	–1.0101	– 13.6 %
V_3 (cm^{-1})	297.946	– 7.6 %	318.831	– 1.3 %	283.265	– 12.2 %
|μ_a|, |μ_b|, |μ_c| (D)	4.13, 0.49, 1.30		4.30, 0.35, 1.37		3.85, 0.66, 1.17	

[a] Calculated by $[(A_e – A_0)/A_0] \times 100$ % where A_e are the results of computational calculations presented above and A_0 are the results of the global fits determined using XIAM.

[b] Calculated by scanning the ∠(H–C6–C4–N3) or ∠(H–C6–C5–C4) dihedral angle as appropriate for each complex.

[c] Electric dipole moment components along the principal inertial axes.

Table S5- Spectroscopic constants determined for five isotopologues of 4-MT$\cdots$$H_2O$ by *A*- species only (effective) fits performed using PGOPHER (Ref 41).

	4-MT$\cdots$$H_2O$				
	$H_2{}^{16}O$	$H_2{}^{18}O$	DOH	HOD	D_2O
A_0' (MHz)	3863.593(29)[a]	3829.970(45)	3838.701(58)	3810.250(59)	3787.508(75)
B_0' (MHz)	1262.9442(17)	1193.8902(16)	1245.9908(27)	1218.7228(33)	1203.1565(15)
C_0' (MHz)	958.38922(100)	916.1630(13)	947.0710(22)	929.9074(25)	919.5068(18)
D_J (kHz)	0.2380(80)	0.2039(62)	0.231(14)	0.213(20)	0.213(13)
D_{JK} (kHz)	2.309(64)	2.761(61)	2.32(22)	2.24(14)	2.08(28)
d_1 (kHz)	−0.0421(61)	−0.0422(60)	−0.039(12)	−0.051(14)	-
χ_{aa} (N3) (MHz)	−3.247(46)	−3.344(34)	−3.28(14)	−3.27(11)	−3.31(13)
[χ_{bb} (N3) − χ_{cc} (N3)]	−1.30(28)	[−1.30][b]	[−1.30]	[−1.30]	[−1.30]
σ_{rms} (kHz)	22.9	18.0	23.7	23.8	24.1
N	82	77	48	44	38

[a] Numbers in parentheses are one standard deviation in units of the last significant figure.

[b] The value of [χ_{bb} (N3) − χ_{cc} (N3)] fixed to the value determined for 4-MT$\cdots$$H_2{}^{16}O$.

Table S6 - Spectroscopic constants determined for five isotopologues of 5-MT$\cdots H_2O$ by *A*- species only (effective) fits performed using PGOPHER (Ref 41).

	5-MT$\cdots H_2O$				
	$H_2{}^{16}O$	$H_2{}^{18}O$	DOH	HOD	D_2O
A_0' (MHz)	4859.032(66)[a]	4856.524(91)	4833.356(64)	4829.876(14)	4805.957(96)
B_0' (MHz)	1085.71851(67)	1021.60625(67)	1066.47904(62)	1042.9804(12)	1025.58745(86)
C_0' (MHz)	892.51934(75)	848.6413(11)	878.64582(81)	863.11291(93)	850.42008(100)
D_J (kHz)	0.1808(42)	0.1887(36)	0.1793(38)	0.1401(69)	0.1678(59)
D_{JK} (kHz)	12.089(24)	11.430(64)	11.682(67)	7.86(21)	10.85(11)
χ_{aa} (N3) (MHz)	−2.936(62)	−2.939(85)	−2.91(10)	−2.80(15)	−2.55(28)
[χ_{bb} (N3) − χ_{cc} (N3)] (MHz)	−1.17(39)	[−1.17][b]	[−1.17][b]	[−1.17][b]	[−1.17][b]
σ_{rms} (kHz)	16.7	16.5	16.9	20.9	18.9
N	68	64	61	39	52

[a] Numbers in parentheses are one standard deviation in units of the last significant figure.

[b] The value of [χ_{bb} (N3) − χ_{cc} (N3)] fixed to the value determined for 5-MT$\cdots H_2{}^{16}O$.

Table S7- Nuclear quadrupole coupling constants in the principle nuclear axis framework determined using QDIAG.

	4-MT···H_2O[a]	4-Methylthiazole[b]	Thiazole[c]
χ_{xx} (N3) (MHz)	1.55(47)	1.79(60)	1.83(18)
χ_{yy} (N3) (MHz)	2.27(23)	2.5385(66)	2.585(11)
χ_{zz} (N3) (MHz)	−3.82(13)	−4.33(60)	−4.41(18)
	5-MT···H_2O[a]	5-Methylthiazole[b]	Thiazole[c]
χ_{xx} (N3) (MHz)	1.56(52)	1.75(43)	1.83(18)
χ_{yy} (N3) (MHz)	2.05(20)	2.711(14)	2.585(11)
χ_{zz} (N3) (MHz)	−3.62(37)	−4.46(43)	−4.41(18)

[a] Calculated from experimentally determined values of nuclear quadrupole coupling constants, χ_{aa}, χ_{bb} and χ_{cc} (displayed in tables S5 and S6) and the off-diagonal term χ_{ab} calculated at the ωB97X-D/aug-cc-pVQZ level of theory.

[b] Calculated from results provided in references 8 and 9 and the off-diagonal term χ_{ab} calculated at the ωB97X-D/aug-cc-pVQZ level of theory.

[c] Results for thiazole provided in ref 4.

Table S8- Spectroscopic parameters determined from global (XIAM) fits obtained by fitting to the frequencies of *A*- and *E*- species transitions for five isotopologues of 4-MT···H_2O.

	$H_2{}^{16}O$	$H_2{}^{18}O$	DOH[a]	HOD[a]	D_2O
A_0 (MHz)	3863.381(53)[b]	3829.702(62)	3838.532(84)	3809.98(10)	3787.428(93)
B_0 (MHz)	1262.9188(25)	1193.8656(25)	1245.9618(17)	1218.7017(42)	1203.1367(30)
C_0 (MHz)	958.3805(22)	916.1610(22)	947.0629(22)	929.9048(39)	919.4958(33)
D_J (kHz)	0.2430(97)	0.2141(94)	0.198(17)	0.255(19)	0.214(17)
D_{JK} (kHz)	2.123(84)	2.937(86)	2.08(29)	2.23(28)	1.82(38)
d_1 (kHz)	–0.0604(97)	–0.0421(98)	[–0.039]	–0.061(19)	–0.069(16)
χ_{aa} (N3) (MHz)	–3.252(19)	–3.335(22)	–3.198(84)	–3.275(63)	–3.344(63)
[χ_{bb}(N3) – χ_{cc}(N3)] (MHz)	[–1.30]	[–1.30]	[–1.30]	[–1.30]	[–1.30]
F_0 (GHz)	[160.0][c]	[160.0]	[160.0]	[160.0]	[160.0]
V_3 (cm^{-1})	340.05(56)	339.43(70)	339.50(97)	344.2(12)	342.21(11)
∠ (*i*, *b*) (°)	45.38(39)	44.17(46)	45.68(71)	44.72(85)	44.12(74)
Δ_0 (u Å)	–3.6540(23)	–3.6493(27)	–3.6453(32)	–3.859(4)	–3.861(4)
σ_{RMS} (kHz)	21.9	22.9	28.6	28.7	21.9
N_A/N_E[d]	82/54	77/44	48/29	44/17	38/20

[a] The ordering of atoms is H_bOH_{nb} such that H_b is deuterium for the isotopologue denoted as "DOH".

[b] Numbers in parentheses are one standard deviation in units of the last significant figure.

[c] The value of F_0 is fixed to 160 GHz.

[d] N_A and N_E denote the number of *A*- species and *E*- species transitions respectively included in the fit.

Table S9- Spectroscopic parameters determined from global (XIAM) fits obtained by fitting to the frequencies of *A*- and *E*- species transitions for five isotopologues of 5-MT···H_2O.

	$H_2{}^{16}O$	$H_2{}^{18}O$	DOH[a]	HOD[a]	D_2O
A_0 (MHz)	4857.887(94)[b]	4855.47(11)	4832.393(93)	4829.07(27)	4804.82(18)
B_0 (MHz)	1085.7196(10)	1021.60693(83)	1066.48040(90)	1042.9847(25)	1025.5885(16)
C_0 (MHz)	892.5126(11)	848.6339(14)	878.6371(12)	863.1044(23)	850.4142(18)
D_J (kHz)	0.1835(57)	0.1873(46)	0.1753(55)	0.145(11)	0.172(10)
D_{JK} (kHz)	12.051(37)	11.359(78)	11.707(98)	8.10(35)	10.98(20)
χ_{aa}(N3) (MHz)	−2.949(37)	−2.915(39)	−2.907(49)	−2.74(12)	−2.86(10)
[χ_{bb}(N3) − χ_{cc}(N3)] (MHz)	[−1.17]	[−1.17]	[−1.17]	[−1.17]	[−1.17]
F_0 (GHz)	[160.0][c]	[160.0]	[160.0]	[160.0]	[160.0]
V_3 (cm^{-1})	325.16(38)	324.86(36)	324.44(40)	324.82(80)	324.52(72)
∠ (i, b) (°)	71.66(71)	70.90(66)	70.42(71)	70.8(15)	70.1(12)
Δ_0 (u Å)	−3.2681(22)	−3.2541(26)	−3.2719(22)	−3.668(6)	−3.677(4)
σ_{RMS} (kHz)	20.5	17.4	19.1	23.6	25.1
N_A/N_E[d]	68/53	64/61	61/49	39/20	52/39

[a] The ordering of atoms is H_bOH_{nb} such that H_b is deuterium for the isotopologue denoted as "DOH".

[b] Numbers in parentheses are one standard deviation in units of the last significant figure.

[c] The value of F_0 is fixed to 160 GHz.

[d] N_A and N_E denote the number of *A*-species and *E*- species transitions respectively included in the fit.

Table S10- Molecular parameters of 4-Methylthiazole···$H_2{}^{16}O$ and 5-Methylthiazole···$H_2{}^{16}O$ (main isotopologues) in the rho axis system (RAM) obtained using the BELGI-C_s-hyperfine code.

Parameter.[a]	Unit	4-MT···H_2O	5-MT···H_2O	Operator
A	MHz	3590.(17)	4838.4(12)	P_a^2
B	MHz	1537.(17)	1105.3(12)	P_b^2
C	MHz	958.37546 (84)	892.5059(11)	P_c^2
D_{ab}	MHz	798.(22)	−270.9(80)	$\{P_a,P_b\}$
Δ_J	kHz	0.1101 (97)	0.1479(41)	$-P^4$
Δ_{JK}	kHz	2.579(58)	12.137(23)	$-P^2P_a^2$
$2\chi_{aa}$	MHz	−7.640(71)	−5.96(11)	
$2\chi_{bb}$	MHz	3.23(21)	1.82(31)	
$2\chi_{ab}$	MHz	0.	0.	
V_3	cm^{-1}	329.(5)	329.(4)	(1/2)(1−cos3α)
ρ	unitless	0.0161(11)	0.0304(16)	P_aP_α
F	cm^{-1}	5.3983[b]	5.4909[b]	P_α^2
N_A / N_E / N_q[c]		82/54/136	68/53/121	
rms[d]	kHz	20.5	17.7	

[a] All parameters refer to the rho axis system and cannot be directly compared to those referred to the principal axis system. P_a, P_b, and P_c are the components of the overall rotation angular momentum, P is the angular momentum of the internal rotor rotating about the internal rotor axis by an angle α. {u,v} is the anti-commutator uv + vu. The product of the parameter and operator from a given row yields the term actually used in the vibration-rotation-torsion Hamiltonian, except for F, and A, which occur in the Hamiltonian in the form $F(P-P_a)^2 + AP_a^2$, where $F = h^2/8\pi^2 crI_\alpha$ (cm^{-1}). Statistical uncertainties are shown as one standard uncertainty in the last digit.

[b] Fixed to the value from the XIAM A/E fit.

[c] Number of A- and E- species transitions as well as hyperfine components.

[d] Root-mean-square deviation of the fit.

Table S11: Observed transition frequencies of *A*- and *E*- species transitions of 4-MT···$H_2{}^{16}O$ main isotopologue. ν_{obs} - ν_{calc} values obtained after fitting with the XIAM and BELGI-Cs hyperfine programs.

J'	K_{-1}'	K_1'	*F'*		*J''*	K_{-1}''	K_1''	*F''*	Symmetry	ν_{obs} (MHz)	ν_{obs} - ν_{calc} (MHz) XIAM	ν_{obs} - ν_{calc} (MHz) BELGI
4	1	4	4	→	3	1	3	3	A	8229.331	0.004	0.011
4	1	4	5	→	3	1	3	4	A	8229.514	0.041	0.053
4	0	4	3	→	3	0	3	2	A	8642.898	0.036	0.037
4	0	4	4	→	3	0	3	3	A	8642.898	-0.029	0.004
4	2	3	4	→	3	2	2	3	A	8865.235	0.011	0.007
4	2	3	5	→	3	2	2	4	A	8865.677	-0.006	0.002
4	2	3	3	→	3	2	2	2	A	8865.809	0.011	0.020
4	2	3	4	→	3	2	2	3	E	8869.335	0.007	0.003
4	2	3	5	→	3	2	2	4	E	8869.777	-0.008	-0.003
4	2	3	3	→	3	2	2	2	E	8869.909	0.009	0.014
4	3	2	4	→	3	3	1	3	A	8931.105	-0.004	-0.005
4	3	2	5	→	3	3	1	4	A	8932.072	0.008	0.003
4	3	2	4	→	3	3	1	3	E	8934.655	-0.020	-0.020
4	3	2	5	→	3	3	1	4	E	8935.621	0.008	-0.011
4	3	1	4	→	3	3	0	3	A	8938.936	0.002	0.000
4	3	1	5	→	3	3	0	4	A	8939.884	-0.008	-0.009
4	3	1	3	→	3	3	0	2	A	8940.265	0.000	0.000
4	3	1	4	→	3	3	0	3	E	8934.972	-0.001	-0.003
4	3	1	5	→	3	3	0	4	E	8935.964	0.035	0.031
4	3	1	3	→	3	3	0	2	E	8936.288	-0.014	-0.017
4	2	2	4	→	3	2	1	3	A	9107.550	0.009	0.005
4	2	2	5	→	3	2	1	4	A	9107.960	-0.002	-0.000
4	2	2	3	→	3	2	1	2	A	9108.085	0.013	0.014
4	2	2	4	→	3	2	1	3	E	9103.030	-0.001	-0.002
4	2	2	5	→	3	2	1	4	E	9103.440	0.000	-0.006
4	2	2	3	→	3	2	1	2	E	9103.565	0.015	0.009
4	1	3	4	→	3	1	2	3	A	9439.711	-0.004	-0.020
4	1	3	3	→	3	1	2	2	A	9439.849	0.024	0.008
4	1	3	5	→	3	1	2	4	A	9439.849	0.003	-0.016
4	1	3	4	→	3	1	2	3	E	9439.384	0.000	-0.016
4	1	3	3	→	3	1	2	2	E	9439.522	0.024	0.012
4	1	3	5	→	3	1	2	4	E	9439.522	0.003	-0.012
5	1	5	4	→	4	1	4	3	A	10247.718	0.024	0.033
5	1	5	6	→	4	1	4	5	A	10247.718	-0.046	-0.012
5	0	5	5	→	4	0	4	4	A	10647.209	-0.011	-0.014
5	0	5	4	→	4	0	4	3	A	10647.209	-0.005	-0.014
5	0	5	5	→	4	0	4	4	E	10647.029	-0.020	-0.030
5	0	5	4	→	4	0	4	3	E	10647.029	-0.005	-0.030
5	2	4	5	→	4	2	3	4	A	11050.312	0.022	0.018
5	2	4	4	→	4	2	3	3	A	11050.556	-0.020	-0.000

5	2	4	5	→	4	2	3	4	E	11051.392	0.026	0.021
5	2	4	4	→	4	2	3	3	E	11051.643	-0.013	0.009
5	3	3	5	→	4	3	2	4	A	11179.711	-0.001	-0.003
5	3	3	6	→	4	3	2	5	A	11180.199	-0.006	-0.009
5	3	3	4	→	4	3	2	3	A	11180.335	0.008	0.004
5	3	3	5	→	4	3	2	4	E	11189.825	-0.007	-0.006
5	3	3	6	→	4	3	2	5	E	11190.315	-0.003	-0.010
5	3	3	4	→	4	3	2	3	E	11190.450	0.010	0.004
5	3	2	5	→	4	3	1	4	A	11206.841	0.003	0.002
5	3	2	6	→	4	3	1	5	A	11207.366	0.036	0.036
5	3	2	4	→	4	3	1	3	A	11207.459	0.007	0.008
5	3	2	5	→	4	3	1	4	E	11196.222	0.005	0.004
5	3	2	6	→	4	3	1	5	E	11196.711	-0.002	0.002
5	3	2	4	→	4	3	1	3	E	11196.850	0.016	0.020
5	2	3	5	→	4	2	2	4	A	11509.166	0.011	0.005
5	2	3	6	→	4	2	2	5	A	11509.373	0.008	0.009
5	2	3	4	→	4	2	2	3	A	11509.373	-0.013	-0.012
5	2	3	5	→	4	2	2	4	E	11507.566	0.009	0.008
5	2	3	6	→	4	2	2	5	E	11507.773	0.008	0.012
5	2	3	4	→	4	2	2	3	E	11507.773	-0.013	-0.009
5	1	4	6	→	4	1	3	5	A	11743.322	-0.033	-0.048
5	1	4	4	→	4	1	3	3	A	11743.322	0.023	-0.024
5	1	4	6	→	4	1	3	5	E	11742.957	-0.019	-0.036
5	1	4	4	→	4	1	3	3	E	11742.957	0.023	-0.011
6	0	6	5	→	5	0	5	4	A	12588.589	-0.026	-0.020
6	0	6	6	→	5	0	5	5	A	12588.589	0.018	-0.008
6	0	6	5	→	5	0	5	4	E	12588.457	0.015	0.009
6	0	6	6	→	5	0	5	5	E	12588.457	0.018	0.022
6	2	5	6	→	5	2	4	5	A	13214.691	0.020	0.017
6	2	5	7	→	5	2	4	6	A	13214.874	0.036	0.055
6	2	5	5	→	5	2	4	4	A	13214.874	0.035	0.054
6	4	3	6	→	5	4	2	5	A	13412.059	-0.030	-0.026
6	4	3	7	→	5	4	2	6	A	13412.571	0.006	-0.021
6	4	2	6	→	5	4	1	5	A	13414.106	0.003	0.006
6	4	2	7	→	5	4	1	6	A	13414.578	-0.034	-0.028
6	3	4	6	→	5	3	3	5	A	13432.240	-0.002	-0.003
6	3	4	7	→	5	3	3	6	A	13432.518	-0.011	-0.014
6	3	4	6	→	5	3	3	5	E	13445.864	-0.001	-0.002
6	3	4	7	→	5	3	3	6	E	13446.129	-0.025	-0.027
6	3	3	6	→	5	3	2	5	A	13503.305	0.003	0.003
6	3	3	7	→	5	3	2	6	A	13503.564	-0.021	-0.020
6	3	3	6	→	5	3	2	5	E	13489.071	0.003	0.009
6	3	3	7	→	5	3	2	6	E	13489.325	-0.026	-0.019
6	2	4	7	→	5	2	3	6	A	13951.941	-0.004	-0.016
6	2	4	5	→	5	2	3	4	A	13951.941	0.002	-0.014
6	2	4	7	→	5	2	3	6	E	13951.071	-0.009	-0.014

6	2	4	5	→	5	2	3	4	E	13951.071	0.002	-0.012
6	1	5	7	→	5	1	4	6	A	14002.219	0.007	-0.008
6	1	5	5	→	5	1	4	4	A	14002.219	0.018	0.011
6	1	5	7	→	5	1	4	6	E	14001.793	0.011	-0.007
6	1	5	5	→	5	1	4	4	E	14001.793	0.018	0.012
7	1	7	8	→	6	1	6	7	A	14225.252	-0.021	-0.010
7	1	7	6	→	6	1	6	5	A	14225.252	0.026	0.016
7	1	7	7	→	6	1	6	6	A	14225.252	0.043	0.031
7	2	6	8	→	6	2	5	7	A	15355.083	-0.025	-0.025
7	2	6	6	→	6	2	5	5	A	15355.083	0.006	-0.019
7	3	5	7	→	6	3	4	6	A	15684.734	0.014	0.014
7	3	5	8	→	6	3	4	7	A	15684.916	-0.002	0.012
7	3	5	6	→	6	3	4	5	A	15684.916	-0.020	-0.005
7	3	5	7	→	6	3	4	6	E	15693.013	0.008	0.006
7	3	5	8	→	6	3	4	7	E	15693.206	0.008	0.014
7	3	5	6	→	6	3	4	5	E	15693.206	-0.010	-0.003
7	3	4	7	→	6	3	3	6	A	15839.850	0.015	0.016
7	3	4	8	→	6	3	3	7	A	15840.023	0.002	0.016
7	3	4	6	→	6	3	3	5	A	15840.023	-0.014	0.000
7	3	4	7	→	6	3	3	6	E	15830.833	0.010	0.022
7	3	4	8	→	6	3	3	7	E	15830.982	-0.021	-0.001
7	3	4	6	→	6	3	3	5	E	15830.982	-0.036	-0.017
8	1	8	7	→	7	1	7	6	A	16187.709	-0.033	-0.024
8	1	8	8	→	7	1	7	7	A	16187.709	0.013	-0.012
8	1	8	9	→	7	1	7	8	A	16187.709	-0.021	-0.044
7	1	6	8	→	6	1	5	7	A	16201.811	0.002	-0.011
7	1	6	7	→	6	1	5	6	A	16201.811	0.052	0.038
7	1	6	6	→	6	1	5	5	A	16201.811	0.014	0.003
7	1	6	8	→	6	1	5	7	E	16201.359	0.020	0.000
7	1	6	7	→	6	1	5	6	E	16201.359	0.052	0.049
7	1	6	6	→	6	1	5	5	E	16201.359	0.014	0.015
8	0	8	8	→	7	0	7	7	A	16377.769	-0.019	-0.016
8	0	8	9	→	7	0	7	8	A	16377.769	-0.038	-0.051
8	0	8	7	→	7	0	7	6	A	16377.769	-0.018	-0.031
7	2	5	8	→	6	2	4	7	A	16406.542	-0.003	-0.015
7	2	5	7	→	6	2	4	6	A	16406.542	0.065	0.052
7	2	5	6	→	6	2	4	5	A	16406.542	0.007	-0.008
7	2	5	8	→	6	2	4	7	E	16405.832	-0.034	-0.039
7	2	5	7	→	6	2	4	6	E	16405.832	0.065	0.029
7	2	5	6	→	6	2	4	5	E	16405.832	0.007	-0.031
9	1	9	8	→	8	1	8	7	A	18136.536	0.024	0.030
9	1	9	10	→	8	1	8	9	A	18136.536	-0.017	0.014
9	0	9	8	→	8	0	8	7	A	18264.548	0.014	0.018
9	0	9	10	→	8	0	8	9	A	18264.548	-0.016	0.002
8	1	7	9	→	7	1	6	8	A	18327.957	-0.011	-0.023
8	1	7	8	→	7	1	6	7	A	18327.957	0.050	0.022

8	1	7	7	→	7	1	6	6	A	18327.957	0.011	-0.012
8	1	7	9	→	7	1	6	8	E	18327.489	0.016	-0.009
8	1	7	8	→	7	1	6	7	E	18327.489	0.050	0.037
8	1	7	7	→	7	1	6	6	E	18327.489	0.011	0.003

Table S12: Observed transition frequencies of *A*- and *E*- species transitions of 4-MT···$H_2^{18}O$. ν_{obs} - ν_{calc} values obtained after fitting using the XIAM program.

J'	K_{-1}'	K_1'	*F'*		*J"*	K_{-1}"	K_1"	*F"*	Symmetry	ν_{obs} (MHz)	ν_{obs} - ν_{calc} (MHz)
4	1	4	4	→	3	1	3	3	A	7845.788	0.010
4	1	4	5	→	3	1	3	4	A	7845.939	0.006
4	0	4	5	→	3	0	3	4	A	8238.781	-0.029
4	2	3	4	→	3	2	2	3	A	8423.546	0.012
4	2	3	5	→	3	2	2	4	A	8423.975	-0.030
4	2	3	3	→	3	2	2	2	A	8424.120	-0.003
4	2	3	4	→	3	2	2	3	E	8428.167	-0.005
4	2	3	5	→	3	2	2	4	E	8428.627	-0.001
4	2	3	3	→	3	2	2	2	E	8428.761	0.014
4	3	2	4	→	3	3	1	3	A	8478.063	0.010
4	3	2	5	→	3	3	1	4	A	8479.033	-0.014
4	3	2	3	→	3	3	1	2	A	8479.435	0.006
4	3	1	4	→	3	3	0	3	A	8483.905	0.007
4	3	1	5	→	3	3	0	4	A	8484.900	0.013
4	3	1	3	→	3	3	0	2	A	8485.276	0.007
4	2	2	4	→	3	2	1	3	A	8624.909	-0.008
4	2	2	5	→	3	2	1	4	A	8625.335	0.001
4	2	2	3	→	3	2	1	2	A	8625.465	0.018
4	2	2	4	→	3	2	1	3	E	8619.903	0.004
4	2	2	5	→	3	2	1	4	E	8620.323	-0.003
4	2	2	3	→	3	2	1	2	E	8620.453	0.015
4	1	3	4	→	3	1	2	3	A	8950.800	-0.018
4	1	3	3	→	3	1	2	2	A	8950.940	0.025
4	1	3	4	→	3	1	2	3	E	8950.487	-0.017
4	1	3	3	→	3	1	2	2	E	8950.624	0.022
5	1	5	4	→	4	1	4	3	A	9774.475	-0.003
5	1	5	6	→	4	1	4	5	A	9774.475	-0.047
5	0	5	4	→	4	0	4	3	A	10165.125	-0.043
5	0	5	4	→	4	0	4	3	E	10165.025	0.019
5	2	4	5	→	4	2	3	4	A	10503.688	0.018
5	2	4	4	→	4	2	3	3	A	10503.934	-0.024
5	2	4	6	→	4	2	3	5	A	10503.934	0.001
5	2	4	5	→	4	2	3	4	E	10504.973	0.019
5	2	4	4	→	4	2	3	3	E	10505.225	-0.019
5	2	4	6	→	4	2	3	5	E	10505.225	0.007
5	3	3	5	→	4	3	2	4	A	10611.340	-0.007
5	3	3	6	→	4	3	2	5	A	10611.860	0.013
5	3	3	4	→	4	3	2	3	A	10612.000	0.027
5	3	2	5	→	4	3	1	4	A	10631.655	0.008
5	3	2	6	→	4	3	1	5	A	10632.150	-0.008
5	3	2	4	→	4	3	1	3	A	10632.288	0.005
5	3	2	5	→	4	3	1	4	E	10623.101	0.011

5	3	2	6	→	4	3	1	5	E	10623.600	-0.006
5	3	2	4	→	4	3	1	3	E	10623.741	0.011
5	2	3	5	→	4	2	2	4	A	10888.361	-0.015
5	2	3	6	→	4	2	2	5	A	10888.572	0.005
5	2	3	4	→	4	2	2	3	A	10888.572	-0.017
5	2	3	5	→	4	2	2	4	E	10886.590	-0.014
5	2	3	6	→	4	2	2	5	E	10886.806	0.010
5	2	3	4	→	4	2	2	3	E	10886.806	-0.011
5	1	4	5	→	4	1	3	4	A	11142.755	0.026
5	1	4	4	→	4	1	3	3	A	11142.761	-0.053
5	1	4	5	→	4	1	3	4	E	11142.421	0.047
5	1	4	4	→	4	1	3	3	E	11142.421	-0.059
6	1	6	6	→	5	1	5	5	A	11685.791	-0.003
6	1	6	5	→	5	1	5	4	A	11685.791	-0.023
6	0	6	5	→	5	0	5	4	A	12033.163	-0.031
6	0	6	6	→	5	0	5	5	A	12033.163	0.016
6	0	6	5	→	5	0	5	4	E	12033.034	0.003
6	0	6	6	→	5	0	5	5	E	12033.034	0.016
6	2	5	6	→	5	2	4	5	A	12566.663	0.017
6	2	5	7	→	5	2	4	6	A	12566.800	-0.013
6	2	5	5	→	5	2	4	4	A	12566.800	-0.014
6	2	5	6	→	5	2	4	5	E	12566.990	0.030
6	2	5	7	→	5	2	4	6	E	12567.114	-0.026
6	2	5	5	→	5	2	4	4	E	12567.114	-0.027
6	2	4	7	→	5	2	3	6	A	13192.343	-0.028
6	2	4	5	→	5	2	3	4	A	13192.343	0.002
6	2	4	7	→	5	2	3	6	E	13191.426	-0.030
6	2	4	5	→	5	2	3	4	E	13191.426	0.002
6	1	5	5	→	5	1	4	4	A	13298.750	-0.002
6	1	5	7	→	5	1	4	6	A	13298.750	-0.019
6	1	5	5	→	5	1	4	4	E	13298.371	0.023
6	1	5	7	→	5	1	4	6	E	13298.371	-0.019
7	1	7	7	→	6	1	6	6	A	13580.285	0.014
7	1	7	6	→	6	1	6	5	A	13580.285	-0.016
7	1	7	8	→	6	1	6	7	A	13580.285	-0.043
7	0	7	7	→	6	0	6	6	A	13860.740	-0.014
7	0	7	6	→	6	0	6	5	A	13860.740	-0.020
7	2	6	6	→	6	2	5	5	A	14609.681	-0.002
7	2	6	8	→	6	2	5	7	A	14609.681	-0.006
7	4	4	7	→	6	4	3	6	A	14871.110	0.030
7	4	4	8	→	6	4	3	7	A	14871.416	-0.023
7	4	4	7	→	6	4	3	6	E	14873.000	-0.066
7	4	4	8	→	6	4	3	7	E	14873.296	-0.033
7	3	5	7	→	6	3	4	6	A	14887.009	0.014
7	3	5	8	→	6	3	4	7	A	14887.201	0.003
7	3	5	6	→	6	3	4	5	A	14887.201	-0.015
7	3	5	7	→	6	3	4	6	E	14896.451	0.005

7	3	5	8	→	6	3	4	7	E	14896.650	0.009
7	3	5	6	→	6	3	4	5	E	14896.650	-0.009
7	1	6	8	→	6	1	5	7	A	15407.058	-0.008
7	1	6	8	→	6	1	5	7	E	15406.616	-0.007
8	1	8	8	→	7	1	7	7	A	15459.470	0.011
8	1	8	7	→	7	1	7	6	A	15459.470	-0.013
8	1	8	9	→	7	1	7	8	A	15459.470	-0.034
7	2	5	7	→	6	2	4	6	A	15514.425	-0.001
7	2	5	7	→	6	2	4	6	E	15513.742	-0.004
8	0	8	8	→	7	0	7	7	A	15668.709	-0.027
8	0	8	7	→	7	0	7	6	A	15668.709	-0.019
8	2	7	9	→	7	2	6	8	A	16630.181	0.029
8	2	7	7	→	7	2	6	6	A	16630.181	0.008
8	2	7	9	→	7	2	6	8	E	16630.017	0.057
8	2	7	7	→	7	2	6	6	E	16630.017	0.008
8	3	6	8	→	7	3	5	7	A	17022.315	0.003
8	3	6	9	→	7	3	5	8	A	17022.467	0.023
8	3	6	7	→	7	3	5	6	A	17022.467	0.018
8	3	6	8	→	7	3	5	7	E	17026.542	-0.016
8	3	6	9	→	7	3	5	8	E	17026.711	0.040
8	3	6	7	→	7	3	5	6	E	17026.711	0.034
9	1	9	10	→	8	1	8	9	A	17325.729	0.003
9	1	9	9	→	8	1	8	8	A	17325.729	0.028
9	1	9	8	→	8	1	8	7	A	17325.729	0.017
8	1	7	9	→	7	1	6	8	A	17455.493	-0.019
8	1	7	8	→	7	1	6	7	A	17455.493	0.048
8	1	7	7	→	7	1	6	6	A	17455.493	0.012
8	1	7	9	→	7	1	6	8	E	17455.039	-0.003
8	1	7	8	→	7	1	6	7	E	17455.039	0.048
8	1	7	7	→	7	1	6	6	E	17455.039	0.012
9	0	9	8	→	8	0	8	7	A	17472.577	-0.012
9	0	9	10	→	8	0	8	9	A	17472.577	-0.016

Table S13: Observed transition frequencies of *A*- and *E*- species transitions of 4-MT···DOH. ν_{obs} - ν_{calc} values obtained after fitting using the XIAM program.

J'	*K*-1'	*K*1'	*F'*		*J"*	*K*-1"	*K*1"	*F"*	Symmetry	ν_{obs} (MHz)	ν_{obs} - ν_{calc} (MHz)
4	1	4	4	→	3	1	3	3	A	8129.025	-0.008
4	1	4	5	→	3	1	3	4	A	8129.175	0.013
4	0	4	5	→	3	0	3	4	A	8537.544	-0.032
4	0	4	4	→	3	0	3	3	A	8537.544	0.060
4	0	4	5	→	3	0	3	4	E	8537.465	0.045
4	0	4	4	→	3	0	3	3	E	8537.465	0.060
4	2	3	4	→	3	2	2	3	A	8752.772	-0.008
4	2	3	5	→	3	2	2	4	A	8753.235	0.033
4	2	3	4	→	3	2	2	3	E	8757.045	-0.020
4	2	3	5	→	3	2	2	4	E	8757.472	-0.005
4	2	2	4	→	3	2	1	3	A	8987.400	0.018
4	2	2	4	→	3	2	1	3	E	8982.750	0.038
4	1	3	4	→	3	1	2	3	A	9317.211	0.040
4	1	3	3	→	3	1	2	2	A	9317.351	0.030
4	1	3	5	→	3	1	2	4	A	9317.351	0.010
4	1	3	4	→	3	1	2	3	E	9316.867	0.015
4	1	3	3	→	3	1	2	2	E	9317.013	0.036
4	1	3	5	→	3	1	2	4	E	9317.013	0.016
5	1	5	5	→	4	1	4	4	A	10123.470	0.014
5	1	5	4	→	4	1	4	3	A	10123.470	-0.037
5	0	5	5	→	4	0	4	4	A	10519.811	-0.069
5	0	5	4	→	4	0	4	3	A	10519.811	-0.007
5	0	5	5	→	4	0	4	4	E	10519.711	-0.002
5	0	5	4	→	4	0	4	3	E	10519.711	-0.007
5	2	4	5	→	4	2	3	4	A	10910.787	0.016
5	2	4	6	→	4	2	3	5	A	10911.007	-0.010
5	2	4	5	→	4	2	3	4	E	10911.914	-0.003
5	2	4	6	→	4	2	3	5	E	10912.167	0.023
5	2	3	5	→	4	2	2	4	A	11355.689	0.035
5	2	3	6	→	4	2	2	5	A	11355.902	0.024
5	2	3	4	→	4	2	2	3	A	11355.902	0.004
5	2	3	5	→	4	2	2	4	E	11354.031	0.016
5	2	3	6	→	4	2	2	5	E	11354.245	0.025
5	2	3	4	→	4	2	2	3	E	11354.245	0.005
5	1	4	6	→	4	1	3	5	A	11592.181	-0.002
5	1	4	4	→	4	1	3	3	A	11592.181	0.021
5	1	4	6	→	4	1	3	5	E	11591.799	-0.021
5	1	4	4	→	4	1	3	3	E	11591.799	0.021
6	2	5	6	→	5	2	4	5	A	13048.766	0.015
6	2	5	5	→	5	2	4	4	A	13048.921	0.013
6	2	5	7	→	5	2	4	6	A	13048.921	0.014
6	2	5	5	→	5	2	4	4	E	13049.175	0.025

6	2	5	7	→	5	2	4	6	E	13049.175	0.001
6	2	4	7	→	5	2	3	6	A	13764.943	0.011
6	2	4	5	→	5	2	3	4	A	13764.943	0.002
6	2	4	7	→	5	2	3	6	E	13764.049	-0.018
6	2	4	5	→	5	2	3	4	E	13764.049	0.002
6	1	5	5	→	5	1	4	4	A	13824.062	0.013
6	1	5	7	→	5	1	4	6	A	13824.062	-0.017
6	1	5	5	→	5	1	4	4	E	13823.625	-0.012
6	1	5	7	→	5	1	4	6	E	13823.625	-0.017
7	1	7	6	→	6	1	6	5	A	14054.654	-0.021
7	1	7	8	→	6	1	6	7	A	14054.654	-0.025
7	1	7	7	→	6	1	6	6	A	14054.654	0.016
7	0	7	7	→	6	0	6	6	A	14321.268	-0.042
7	0	7	8	→	6	0	6	7	A	14321.268	-0.046
7	0	7	6	→	6	0	6	5	A	14321.268	-0.021
7	3	4	6	→	6	3	3	5	A	15631.533	-0.028
7	3	4	8	→	6	3	3	7	A	15631.533	0.015
7	3	4	6	→	6	3	3	5	E	15622.190	-0.033
7	3	4	8	→	6	3	3	7	E	15622.190	0.015
8	1	8	8	→	7	1	7	7	A	15994.489	0.027
8	1	8	7	→	7	1	7	6	A	15994.489	-0.013
8	1	8	9	→	7	1	7	8	A	15994.489	-0.033
7	1	6	7	→	6	1	5	6	A	15998.801	-0.002
7	1	6	6	→	6	1	5	5	A	15998.801	-0.038
7	1	6	8	→	6	1	5	7	A	15998.801	-0.051
7	1	6	7	→	6	1	5	6	E	15998.350	0.000
7	1	6	6	→	6	1	5	5	E	15998.350	-0.038
7	1	6	8	→	6	1	5	7	E	15998.350	-0.051
8	0	8	8	→	7	0	7	7	A	16186.462	0.001
8	0	8	7	→	7	0	7	6	A	16186.462	-0.019
8	0	8	9	→	7	0	7	8	A	16186.462	-0.038
7	2	5	8	→	6	2	4	7	A	16187.039	-0.057
7	2	5	6	→	6	2	4	5	A	16187.039	0.007
8	2	7	8	→	7	2	6	7	A	17252.268	0.044
8	2	7	8	→	7	2	6	7	E	17252.043	0.030

Table S14: Observed transition frequencies of *A*- and *E*- species transitions of 4-MT···HOD. ν_{obs} - ν_{calc} values obtained after fitting using the XIAM program.

J'	K_{-1}'	K_1'	*F'*		*J''*	K_{-1}''	K_1''	*F''*	Symmetry	ν_{obs} (MHz)	ν_{obs} - ν_{calc} (MHz)
4	1	4	5	→	3	1	3	4	A	7974.484	0.024
4	0	4	5	→	3	0	3	4	A	8374.424	-0.001
4	0	4	5	→	3	0	3	4	E	8374.250	-0.029
4	2	3	4	→	3	2	2	3	A	8576.297	-0.002
4	2	3	5	→	3	2	2	4	A	8576.742	-0.006
4	2	3	3	→	3	2	2	2	A	8576.872	0.008
4	2	3	4	→	3	2	2	3	E	8580.150	0.008
4	2	3	5	→	3	2	2	4	E	8580.602	-0.001
4	2	3	3	→	3	2	2	2	E	8580.742	0.023
4	2	2	5	→	3	2	1	4	A	8796.781	0.002
4	2	2	5	→	3	2	1	4	E	8792.575	0.007
4	1	3	5	→	3	1	2	4	A	9122.911	-0.020
4	1	3	5	→	3	1	2	4	E	9122.610	-0.024
5	1	5	4	→	4	1	4	3	A	9932.318	-0.001
5	1	5	6	→	4	1	4	5	A	9932.318	-0.046
5	1	5	5	→	4	1	4	4	A	9932.318	0.038
5	2	4	4	→	4	2	3	3	A	10692.368	-0.009
5	3	3	5	→	4	3	2	4	A	10809.715	-0.002
5	3	3	6	→	4	3	2	5	A	10810.223	0.010
5	3	2	5	→	4	3	1	4	A	10833.155	-0.015
5	3	2	6	→	4	3	1	5	A	10833.661	0.012
5	3	2	5	→	4	3	1	4	E	10823.836	0.020
5	3	2	6	→	4	3	1	5	E	10824.338	0.007
5	2	3	5	→	4	2	2	4	A	11110.626	0.009
5	2	3	6	→	4	2	2	5	A	11110.825	-0.002
5	2	3	4	→	4	2	2	3	A	11110.825	-0.023
5	2	3	5	→	4	2	2	4	E	11109.110	-0.020
5	2	3	6	→	4	2	2	5	E	11109.329	0.018
5	2	3	4	→	4	2	2	3	E	11109.329	-0.003
5	1	4	5	→	4	1	3	4	A	11352.878	0.026
5	1	4	6	→	4	1	3	5	A	11352.878	-0.082
5	1	4	4	→	4	1	3	3	A	11352.878	-0.059
5	1	4	5	→	4	1	3	4	E	11352.552	0.039
5	1	4	6	→	4	1	3	5	E	11352.552	-0.082
5	1	4	4	→	4	1	3	3	E	11352.552	-0.059
6	0	6	6	→	5	0	5	5	A	12213.626	-0.019
6	0	6	5	→	5	0	5	4	A	12213.626	-0.017
6	0	6	6	→	5	0	5	5	E	12213.500	0.010
6	0	6	5	→	5	0	5	4	E	12213.500	-0.017
6	2	4	7	→	5	2	3	6	A	13465.541	-0.006
6	2	4	5	→	5	2	3	4	A	13465.541	0.002
6	2	4	7	→	5	2	3	6	E	13464.750	0.001

6	2	4	5	→	5	2	3	4	E	13464.750	0.002
7	1	7	7	→	6	1	6	6	A	13793.332	0.020
7	1	7	8	→	6	1	6	7	A	13793.332	-0.043
7	1	7	6	→	6	1	6	5	A	13793.332	-0.016
7	0	7	6	→	6	0	6	5	A	14063.582	-0.019
7	0	7	8	→	6	0	6	7	A	14063.582	-0.026
7	0	7	7	→	6	0	6	6	A	14063.582	0.020
7	1	6	8	→	6	1	5	7	A	15680.109	0.009
7	1	6	7	→	6	1	5	6	A	15680.109	0.052
7	1	6	6	→	6	1	5	5	A	15680.109	0.014
8	1	8	9	→	7	1	7	8	A	15699.020	0.001
8	1	8	7	→	7	1	7	6	A	15699.020	0.021
7	2	5	8	→	6	2	4	7	A	15835.414	-0.001
7	2	5	6	→	6	2	4	5	A	15835.414	0.007
7	2	5	8	→	6	2	4	7	E	15834.785	-0.011
7	2	5	6	→	6	2	4	5	E	15834.785	0.007
8	0	8	9	→	7	0	7	8	A	15896.285	-0.001
8	0	8	8	→	7	0	7	7	A	15896.285	0.039
8	0	8	7	→	7	0	7	6	A	15896.285	0.020

Table S15: Observed transition frequencies of *A*- and *E*- species transitions of 4-MT···D_2O. ν_{obs} - ν_{calc} values obtained after fitting using the XIAM program.

J'	K_{-1}'	K_1'	*F*'		*J*"	K_{-1}"	K_1"	*F*"	Symmetry	ν_{obs} (MHz)	ν_{obs} - ν_{calc} (MHz)
4	2	3	4	→	3	2	2	4	A	8472.998	0.008
4	2	3	4	→	3	2	2	3	A	8473.000	0.002
4	2	3	4	→	3	2	2	4	E	8477.001	-0.004
4	2	3	4	→	3	2	2	3	E	8477.001	0.002
4	2	2	4	→	3	2	1	3	A	8686.250	0.013
4	2	2	4	→	3	2	1	4	A	8686.291	-0.003
4	2	2	5	→	3	2	1	4	A	8686.678	0.002
4	2	2	3	→	3	2	1	3	A	8686.709	-0.021
4	2	2	3	→	3	2	1	2	A	8686.799	0.010
4	1	3	4	→	3	1	2	3	A	9010.215	-0.009
4	1	3	3	→	3	1	2	2	A	9010.350	0.020
4	1	3	5	→	3	1	2	4	A	9010.350	-0.003
4	1	3	4	→	3	1	2	3	E	9009.904	-0.017
4	1	3	3	→	3	1	2	2	E	9010.058	0.039
4	1	3	5	→	3	1	2	4	E	9010.058	0.016
5	1	5	6	→	4	1	4	5	A	9818.090	-0.014
5	1	5	4	→	4	1	4	3	A	9818.090	0.047
5	0	5	6	→	4	0	4	5	A	10206.839	-0.021
5	2	4	5	→	4	2	3	4	A	10563.900	0.005
5	2	4	6	→	4	2	3	5	A	10564.164	0.019
5	2	4	4	→	4	2	3	3	A	10564.164	-0.007
5	2	3	5	→	4	2	2	4	A	10969.951	0.006
5	2	3	6	→	4	2	2	5	A	10970.174	0.017
5	2	3	4	→	4	2	2	3	A	10970.174	-0.005
5	2	3	5	→	4	2	2	4	E	10968.380	-0.012
5	2	3	6	→	4	2	2	5	E	10968.609	0.023
5	2	3	4	→	4	2	2	3	E	10968.609	0.002
5	1	4	4	→	4	1	3	3	A	11213.931	-0.003
5	1	4	4	→	4	1	3	3	E	11213.579	-0.009
6	1	6	6	→	5	1	5	5	A	11735.934	0.009
6	1	6	5	→	5	1	5	4	A	11735.934	-0.023
6	0	6	5	→	5	0	5	4	A	12076.925	-0.008
6	0	6	7	→	5	0	5	6	A	12076.925	-0.036
6	0	6	6	→	5	0	5	5	A	12076.925	0.017
6	0	6	5	→	5	0	5	4	E	12076.797	0.021
6	0	6	7	→	5	0	5	6	E	12076.797	-0.036
6	0	6	6	→	5	0	5	5	E	12076.797	0.017
6	1	5	5	→	5	1	4	4	A	13379.075	0.033
6	1	5	7	→	5	1	4	6	A	13379.075	-0.019
6	1	5	5	→	5	1	4	4	E	13378.678	0.028
6	1	5	7	→	5	1	4	6	E	13378.678	-0.019
7	1	7	7	→	6	1	6	6	A	13636.395	0.006

7	1	7	6	→	6	1	6	5	A	13636.395	-0.016
7	0	7	7	→	6	0	6	6	A	13907.500	-0.006
7	0	7	6	→	6	0	6	5	A	13907.500	-0.020
7	0	7	8	→	6	0	6	7	A	13907.500	-0.046
8	1	8	8	→	7	1	7	7	A	15521.265	0.037
8	1	8	7	→	7	1	7	6	A	15521.265	-0.013
8	1	8	9	→	7	1	7	8	A	15521.265	-0.034
7	2	5	8	→	6	2	4	7	A	15633.856	-0.027
7	2	5	6	→	6	2	4	5	A	15633.856	0.008
7	2	5	8	→	6	2	4	7	E	15633.247	0.003
7	2	5	6	→	6	2	4	5	E	15633.247	0.008
8	0	8	9	→	7	0	7	8	A	15720.309	-0.042
8	0	8	8	→	7	0	7	7	A	15720.309	0.039
8	0	8	7	→	7	0	7	6	A	15720.309	0.020
8	3	5	9	→	7	3	4	8	A	17375.336	0.001
8	3	5	7	→	7	3	4	6	A	17375.336	-0.004

Table S16: Observed transition frequencies of *A*- and *E*- species transitions of 5-MT···$H_2{}^{16}O$ main isotopologue. ν_{obs} - ν_{calc} values obtained after fitting with the XIAM and BELGI-Cs hyperfine programs.

J'	K_{-1}'	K_1'	*F*'		*J*"	K_{-1}"	K_1"	*F*"	Symmetry	ν_{obs} (MHz)	ν_{obs} - ν_{calc} (MHz) XIAM	ν_{obs} - ν_{calc} (MHz) BELGI
4	1	4	3	→	3	1	3	2	A	7512.466	0.024	0.043
4	1	4	5	→	3	1	3	4	A	7512.466	-0.055	-0.012
4	1	4	3	→	3	1	3	2	E	7512.790	0.012	0.030
4	1	4	5	→	3	1	3	4	E	7512.790	-0.054	-0.024
4	0	4	4	→	3	0	3	3	A	7841.164	-0.023	-0.015
4	2	3	4	→	3	2	2	3	A	7906.587	-0.011	-0.006
4	2	3	5	→	3	2	2	4	A	7907.009	0.018	0.010
4	2	3	4	→	3	2	2	3	E	7925.413	-0.018	-0.022
4	2	3	5	→	3	2	2	4	E	7925.921	0.103	0.080
4	2	2	4	→	3	2	1	3	A	7978.318	0.004	0.005
4	2	2	5	→	3	2	1	4	A	7978.688	-0.016	-0.012
4	2	2	4	→	3	2	1	3	E	7959.429	-0.001	0.008
4	2	2	5	→	3	2	1	4	E	7959.811	-0.003	0.004
4	1	3	3	→	3	1	2	2	A	8284.250	0.007	-0.002
4	1	3	5	→	3	1	2	4	A	8284.250	-0.020	-0.023
4	1	3	3	→	3	1	2	2	E	8283.834	0.009	0.001
4	1	3	5	→	3	1	2	4	E	8283.834	-0.020	-0.019
5	2	4	5	→	4	2	3	4	A	9874.398	-0.002	0.003
5	2	4	6	→	4	2	3	5	A	9874.618	0.006	0.009
5	2	4	4	→	4	2	3	3	A	9874.618	-0.016	-0.013
5	2	4	5	→	4	2	3	4	E	9888.862	-0.005	-0.012
5	2	4	6	→	4	2	3	5	E	9889.079	-0.001	-0.013
5	2	4	4	→	4	2	3	3	E	9889.079	-0.024	-0.037
5	2	3	5	→	4	2	2	4	A	10016.054	0.009	0.008
5	2	3	4	→	4	2	2	3	A	10016.238	-0.027	-0.020
5	2	3	6	→	4	2	2	5	A	10016.238	-0.007	-0.000
5	2	3	5	→	4	2	2	4	E	10001.525	0.000	0.009
5	2	3	4	→	4	2	2	3	E	10001.710	-0.021	-0.012
5	2	3	6	→	4	2	2	5	E	10001.710	-0.003	0.006
5	1	4	6	→	4	1	3	5	A	10340.526	-0.021	-0.030
5	1	4	4	→	4	1	3	3	A	10340.526	0.022	-0.008
5	1	4	6	→	4	1	3	5	E	10340.258	-0.029	-0.036
5	1	4	4	→	4	1	3	3	E	10340.258	0.022	-0.015
6	2	5	6	→	5	2	4	5	A	11836.100	0.005	0.010
6	2	5	7	→	5	2	4	6	A	11836.243	0.014	0.023
6	2	5	5	→	5	2	4	4	A	11836.243	0.014	0.023
6	2	5	6	→	5	2	4	5	E	11843.415	0.001	-0.001
6	2	5	7	→	5	2	4	6	E	11843.561	0.015	0.013
6	2	5	5	→	5	2	4	4	E	11843.561	0.014	0.012
6	3	4	6	→	5	3	3	5	A	11902.974	0.010	0.013

6	3	4	7	→	5	3	3	6	A	11903.216	-0.021	-0.009
6	3	4	6	→	5	3	3	5	E	11907.515	0.002	0.003
6	3	4	7	→	5	3	3	6	E	11907.757	-0.020	-0.018
6	3	3	6	→	5	3	2	5	A	11912.391	0.004	0.006
6	3	3	7	→	5	3	2	6	A	11912.620	-0.032	-0.027
6	3	3	6	→	5	3	2	5	E	11907.757	0.000	0.002
6	3	3	7	→	5	3	2	6	E	11907.990	-0.029	-0.029
6	2	4	7	→	5	2	3	6	A	12078.710	-0.012	-0.016
6	2	4	7	→	5	2	3	6	E	12071.330	-0.019	-0.018
7	1	7	6	→	6	1	6	5	A	13086.282	-0.034	-0.020
7	1	7	7	→	6	1	6	6	A	13086.282	0.011	-0.009
7	2	6	8	→	6	2	5	7	A	13790.685	0.027	0.031
7	2	6	6	→	6	2	5	5	A	13790.685	0.006	0.037
7	2	6	8	→	6	2	5	7	E	13794.115	-0.001	0.000
7	2	6	6	→	6	2	5	5	E	13794.115	0.006	0.006
7	5	3	7	→	6	5	2	6	A	13868.524	-0.026	-0.025
7	5	3	8	→	6	5	2	7	A	13868.996	0.017	-0.010
7	5	2	7	→	6	5	1	6	A	13868.524	-0.029	-0.028
7	5	2	8	→	6	5	1	7	A	13868.996	0.017	-0.013
7	3	5	7	→	6	3	4	6	A	13895.705	0.019	0.021
7	3	5	6	→	6	3	4	5	A	13895.875	-0.013	0.007
7	3	5	7	→	6	3	4	6	E	13905.025	0.012	0.011
7	3	5	6	→	6	3	4	5	E	13905.195	-0.012	-0.001
7	3	4	7	→	6	3	3	6	A	13916.775	0.006	0.007
7	3	4	8	→	6	3	3	7	A	13916.936	-0.003	0.003
7	3	4	8	→	6	3	3	7	A	13916.936	-0.003	0.003
7	3	4	7	→	6	3	3	6	E	13907.362	0.005	0.005
7	3	4	8	→	6	3	3	7	E	13907.537	0.010	0.015
7	3	4	8	→	6	3	3	7	E	13907.537	0.010	0.015
7	2	5	8	→	6	2	4	7	A	14165.768	0.014	0.010
7	2	5	6	→	6	2	4	5	A	14165.768	0.008	0.018
7	2	5	8	→	6	2	4	7	E	14162.260	0.017	0.011
7	2	5	6	→	6	2	4	5	E	14162.260	0.008	0.019
7	1	6	6	→	6	1	5	5	A	14417.630	0.006	0.002
7	1	6	8	→	6	1	5	7	A	14417.630	-0.014	-0.012
7	1	6	7	→	6	1	5	6	A	14417.630	0.021	0.023
7	1	6	6	→	6	1	5	5	E	14417.425	0.005	0.003
7	1	6	8	→	6	1	5	7	E	14417.425	-0.014	-0.011
7	1	6	7	→	6	1	5	6	E	14417.425	0.021	0.024
8	1	8	8	→	7	1	7	7	A	14927.935	-0.008	-0.001
8	1	8	7	→	7	1	7	6	A	14927.935	-0.009	-0.010
8	1	8	9	→	7	1	7	8	A	14927.935	-0.028	-0.029
8	4	5	8	→	7	4	4	7	A	15872.671	0.012	0.012
8	4	5	9	→	7	4	4	8	A	15872.851	-0.018	-0.006
8	4	5	7	→	7	4	4	6	A	15872.851	-0.040	-0.029
8	4	5	8	→	7	4	4	7	E	15873.256	0.011	0.010

8	4	5	9	→	7	4	4	8	E	15873.475	0.022	0.030
8	4	5	7	→	7	4	4	6	E	15873.475	-0.001	0.008
8	4	4	8	→	7	4	3	7	A	15873.734	-0.005	-0.004
8	4	4	7	→	7	4	3	6	A	15873.959	0.005	0.000
8	4	4	8	→	7	4	3	7	E	15873.061	0.016	0.016
8	4	4	7	→	7	4	3	6	E	15873.256	-0.025	-0.010
8	3	6	7	→	7	3	5	6	A	15890.545	0.020	0.020
8	3	6	9	→	7	3	5	8	A	15890.545	0.005	0.025
8	3	6	7	→	7	3	5	6	E	15905.796	0.015	0.012
8	3	6	9	→	7	3	5	8	E	15905.796	0.005	0.016
8	3	5	7	→	7	3	4	6	A	15932.314	0.001	0.001
8	3	5	9	→	7	3	4	8	A	15932.314	0.004	0.005
8	3	5	9	→	7	3	4	8	E	15916.978	0.008	0.008
8	3	5	7	→	7	3	4	6	E	15916.978	-0.004	0.004
8	2	6	9	→	7	2	5	8	A	16273.254	-0.002	-0.004
8	2	6	7	→	7	2	5	6	A	16273.254	0.009	0.005
8	2	6	9	→	7	2	5	8	E	16271.469	-0.022	-0.029
8	2	6	7	→	7	2	5	6	E	16271.469	0.009	-0.020
8	1	7	8	→	7	1	6	7	A	16431.740	0.022	0.022
8	1	7	9	→	7	1	6	8	A	16431.740	-0.030	-0.008
8	1	7	7	→	7	1	6	6	A	16431.740	-0.019	0.003
8	1	7	8	→	7	1	6	7	E	16431.504	0.008	0.012
8	1	7	9	→	7	1	6	8	E	16431.504	-0.030	-0.018
8	1	7	7	→	7	1	6	6	E	16431.504	-0.019	-0.007
9	1	9	9	→	8	1	8	8	A	16761.214	0.018	0.010
9	1	9	10	→	8	1	8	9	A	16761.214	-0.023	-0.013
9	1	9	8	→	8	1	8	7	A	16761.214	-0.008	0.002
9	0	9	9	→	8	0	8	8	A	17080.649	-0.012	-0.019
9	0	9	9	→	8	0	8	8	E	17080.400	-0.013	-0.009
9	2	7	8	→	8	2	6	7	A	18393.898	-0.020	-0.015
9	2	7	9	→	8	2	6	8	A	18393.898	0.012	-0.003
9	2	7	8	→	8	2	6	7	E	18392.953	-0.014	-0.017
9	2	7	9	→	8	2	6	8	E	18392.953	0.012	-0.005
9	1	8	8	→	8	1	7	7	A	18424.326	-0.011	-0.008
9	1	8	9	→	8	1	7	8	A	18424.326	0.019	0.011

Table S17: Observed transition frequencies of *A*- and *E*- species transitions of 5-MT···$H_2{}^{18}O$. ν_{obs} - ν_{calc} values obtained after fitting using the XIAM program.

J'	K_{-1}'	K_1'	*F*'		*J*"	K_{-1}"	K_1"	*F*"	Symmetry	ν_{obs} (MHz)	ν_{obs} - ν_{calc} (MHz)
4	2	3	4	→	3	2	2	3	A	7475.815	-0.018
4	2	3	5	→	3	2	2	4	A	7476.225	0.011
4	2	3	4	→	3	2	2	3	E	7492.976	-0.012
4	2	3	5	→	3	2	2	4	E	7493.382	0.007
4	2	2	4	→	3	2	1	3	A	7532.662	-0.008
4	2	2	5	→	3	2	1	4	A	7533.056	0.011
4	2	2	4	→	3	2	1	3	E	7515.454	-0.007
4	2	2	5	→	3	2	1	4	E	7515.844	0.007
4	1	3	4	→	3	1	2	3	A	7814.885	-0.016
4	1	3	3	→	3	1	2	2	A	7815.011	0.030
4	1	3	5	→	3	1	2	4	A	7815.011	0.010
4	1	3	4	→	3	1	2	3	E	7814.450	0.004
4	1	3	3	→	3	1	2	2	E	7814.550	0.005
4	1	3	5	→	3	1	2	4	E	7814.550	-0.015
5	2	4	5	→	4	2	3	4	A	9337.826	-0.002
5	2	4	6	→	4	2	3	5	A	9338.044	0.007
5	2	4	4	→	4	2	3	3	A	9338.044	-0.015
5	2	4	5	→	4	2	3	4	E	9353.282	-0.010
5	2	4	6	→	4	2	3	5	E	9353.517	0.021
5	2	4	4	→	4	2	3	3	E	9353.517	-0.002
5	2	3	5	→	4	2	2	4	A	9450.379	-0.005
5	2	3	4	→	4	2	2	3	A	9450.574	-0.015
5	2	3	6	→	4	2	2	5	A	9450.574	0.004
5	2	3	5	→	4	2	2	4	E	9434.860	-0.002
5	2	3	4	→	4	2	2	3	E	9435.045	-0.021
5	2	3	6	→	4	2	2	5	E	9435.045	-0.002
5	1	4	4	→	4	1	3	3	A	9757.148	-0.004
5	1	4	6	→	4	1	3	5	A	9757.148	-0.021
5	1	4	4	→	4	1	3	3	E	9756.876	-0.001
5	1	4	6	→	4	1	3	5	E	9756.876	-0.021
6	2	5	6	→	5	2	4	5	A	11194.966	0.000
6	2	5	7	→	5	2	4	6	A	11195.111	0.018
6	2	5	5	→	5	2	4	4	A	11195.111	0.018
6	2	5	6	→	5	2	4	5	E	11203.692	-0.004
6	2	5	7	→	5	2	4	6	E	11203.839	0.017
6	2	5	5	→	5	2	4	4	E	11203.839	0.016
6	2	4	7	→	5	2	3	6	A	11388.657	-0.007
6	2	4	5	→	5	2	3	4	A	11388.657	0.002
6	2	4	5	→	5	2	3	4	E	11379.842	-0.026
6	2	4	7	→	5	2	3	6	E	11379.842	-0.002
6	1	5	5	→	5	1	4	4	A	11690.956	0.020
6	1	5	7	→	5	1	4	6	A	11690.956	-0.018

6	1	5	5	→	5	1	4	4	E	11690.756	0.031
6	1	5	7	→	5	1	4	6	E	11690.756	-0.018
7	2	6	6	→	6	2	5	5	A	13046.470	0.023
7	2	6	8	→	6	2	5	7	A	13046.470	-0.006
7	2	6	6	→	6	2	5	5	E	13050.756	0.005
7	2	6	8	→	6	2	5	7	E	13050.756	-0.006
7	3	5	7	→	6	3	4	6	A	13130.297	-0.003
7	3	5	8	→	6	3	4	7	A	13130.469	0.007
7	3	5	6	→	6	3	4	5	A	13130.469	-0.009
7	3	5	7	→	6	3	4	6	E	13137.136	0.023
7	3	5	8	→	6	3	4	7	E	13137.263	-0.038
7	3	5	6	→	6	3	4	5	E	13137.263	-0.053
7	3	4	7	→	6	3	3	6	A	13145.080	0.007
7	3	4	8	→	6	3	3	7	A	13145.218	-0.025
7	3	4	6	→	6	3	3	5	A	13145.218	-0.040
7	3	4	7	→	6	3	3	6	E	13138.161	-0.010
7	3	4	6	→	6	3	3	5	E	13138.339	-0.001
7	3	4	8	→	6	3	3	7	E	13138.339	0.014
7	2	5	8	→	6	2	4	7	A	13347.904	0.015
7	2	5	6	→	6	2	4	5	A	13347.904	0.008
7	2	5	8	→	6	2	4	7	E	13343.538	0.015
7	2	5	6	→	6	2	4	5	E	13343.538	0.008
7	1	6	6	→	6	1	5	5	A	13613.993	-0.003
7	1	6	7	→	6	1	5	6	A	13613.993	0.020
7	1	6	8	→	6	1	5	7	A	13613.993	-0.014
7	1	6	6	→	6	1	5	5	E	13613.789	-0.011
7	1	6	7	→	6	1	5	6	E	13613.789	0.020
7	1	6	8	→	6	1	5	7	E	13613.789	-0.014
8	0	8	7	→	7	0	7	6	A	14525.956	-0.007
8	0	8	9	→	7	0	7	8	A	14525.956	-0.017
8	0	8	8	→	7	0	7	7	A	14525.956	0.017
8	0	8	7	→	7	0	7	6	E	14525.750	-0.026
8	0	8	9	→	7	0	7	8	E	14525.750	-0.017
8	0	8	8	→	7	0	7	7	E	14525.750	0.017
8	2	7	9	→	7	2	6	8	A	14891.255	0.041
8	2	7	7	→	7	2	6	6	A	14891.255	0.008
8	2	7	9	→	7	2	6	8	E	14893.426	0.046
8	2	7	7	→	7	2	6	6	E	14893.426	0.007
8	3	5	9	→	7	3	4	8	A	15043.810	-0.010
8	3	5	7	→	7	3	4	6	A	15043.810	-0.004
8	2	6	7	→	7	2	5	6	A	15326.142	-0.015
8	2	6	8	→	7	2	5	7	A	15326.142	0.025
8	2	6	9	→	7	2	5	8	A	15326.142	-0.009
8	2	6	7	→	7	2	5	6	E	15323.924	0.001
8	2	6	8	→	7	2	5	7	E	15323.924	0.025
8	2	6	9	→	7	2	5	8	E	15323.924	-0.009

8	1	7	8	→	7	1	6	7	A	15523.634	0.012
8	1	7	7	→	7	1	6	6	A	15523.634	-0.017
8	1	7	9	→	7	1	6	8	A	15523.634	-0.028
8	1	7	8	→	7	1	6	7	E	15523.422	0.006
8	1	7	7	→	7	1	6	6	E	15523.422	-0.017
8	1	7	9	→	7	1	6	8	E	15523.422	-0.028
9	0	9	8	→	8	0	8	7	A	16239.839	-0.008
9	0	9	9	→	8	0	8	8	A	16239.839	0.018
9	0	9	10	→	8	0	8	9	A	16239.839	-0.013
9	0	9	8	→	8	0	8	7	E	16239.618	-0.010
9	0	9	9	→	8	0	8	8	E	16239.618	0.018
9	0	9	10	→	8	0	8	9	E	16239.618	-0.013
9	3	7	8	→	8	3	6	7	A	16899.789	-0.019
9	3	7	10	→	8	3	6	9	A	16899.789	0.000
9	3	7	8	→	8	3	6	7	E	16916.340	-0.022
9	3	7	10	→	8	3	6	9	E	16916.340	0.000
9	3	6	8	→	8	3	5	7	A	16953.058	0.004
9	3	6	10	→	8	3	5	9	A	16953.058	-0.001
9	3	6	8	→	8	3	5	7	E	16936.420	0.012
9	3	6	10	→	8	3	5	9	E	16936.420	-0.001
9	2	7	8	→	8	2	6	7	A	17318.736	0.001
9	2	7	9	→	8	2	6	8	A	17318.736	0.011
9	2	7	10	→	8	2	6	9	A	17318.736	-0.008
9	2	7	8	→	8	2	6	7	E	17317.510	-0.010
9	2	7	9	→	8	2	6	8	E	17317.510	0.011
9	2	7	10	→	8	2	6	9	E	17317.510	-0.008
9	1	8	10	→	8	1	7	9	A	17416.821	-0.013
9	1	8	9	→	8	1	7	8	A	17416.821	0.026
9	1	8	8	→	8	1	7	7	A	17416.821	0.009
9	1	8	10	→	8	1	7	9	E	17416.587	-0.016
9	1	8	8	→	8	1	7	7	E	17416.587	0.009
10	0	10	10	→	9	0	9	9	A	17936.862	-0.001
10	0	10	9	→	9	0	9	8	A	17936.862	-0.018
10	0	10	11	→	9	0	9	10	A	17936.862	-0.029
10	0	10	10	→	9	0	9	9	E	17936.636	0.016
10	0	10	9	→	9	0	9	8	E	17936.636	-0.018
10	0	10	11	→	9	0	9	10	E	17936.636	-0.029

Table S18: Observed transition frequencies of *A*- and *E*- species transitions of 5-MT···DOH. ν_{obs} - ν_{calc} values obtained after fitting using the XIAM program.

J'	K_{-1}'	K_1'	*F*'		*J*"	K_{-1}"	K_1"	*F*"	Symmetry	ν_{obs} (MHz)	ν_{obs} - ν_{calc} (MHz)
4	2	3	4	→	3	2	2	3	A	7774.440	-0.015
4	2	3	5	→	3	2	2	4	A	7774.822	-0.016
4	2	3	4	→	3	2	2	3	E	7792.838	-0.037
4	2	3	5	→	3	2	2	4	E	7793.259	0.022
4	1	3	4	→	3	1	2	3	A	8141.770	-0.002
4	1	3	3	→	3	1	2	2	A	8141.882	0.016
4	1	3	5	→	3	1	2	4	A	8141.882	-0.004
4	1	3	4	→	3	1	2	3	E	8141.350	0.008
4	1	3	3	→	3	1	2	2	E	8141.450	0.004
4	1	3	5	→	3	1	2	4	E	8141.450	-0.016
5	0	5	5	→	4	0	4	4	A	9591.200	0.033
5	0	5	6	→	4	0	4	5	A	9591.200	-0.037
5	0	5	5	→	4	0	4	4	E	9591.027	-0.044
5	0	5	6	→	4	0	4	5	E	9591.027	-0.037
5	2	4	5	→	4	2	3	4	A	9709.693	-0.001
5	2	4	6	→	4	2	3	5	A	9709.924	0.020
5	2	4	4	→	4	2	3	3	A	9709.924	-0.001
5	2	4	5	→	4	2	3	4	E	9724.336	0.005
5	2	4	6	→	4	2	3	5	E	9724.558	0.008
5	2	4	4	→	4	2	3	3	E	9724.558	-0.015
5	2	3	5	→	4	2	2	4	A	9844.043	0.006
5	2	3	6	→	4	2	2	5	A	9844.220	-0.012
5	2	3	4	→	4	2	2	3	A	9844.220	-0.031
5	2	3	5	→	4	2	2	4	E	9829.351	0.017
5	2	3	6	→	4	2	2	5	E	9829.536	0.000
5	2	3	4	→	4	2	2	3	E	9829.536	-0.018
5	1	4	4	→	4	1	3	3	A	10163.366	0.007
5	1	4	6	→	4	1	3	5	A	10163.366	-0.021
5	1	4	4	→	4	1	3	3	E	10163.090	0.000
5	1	4	6	→	4	1	3	5	E	10163.090	-0.021
6	2	5	6	→	5	2	4	5	A	11639.139	-0.004
6	2	5	7	→	5	2	4	6	A	11639.291	0.025
6	2	5	5	→	5	2	4	4	A	11639.291	0.024
6	2	5	6	→	5	2	4	5	E	11646.725	0.022
6	2	5	7	→	5	2	4	6	E	11646.860	0.006
6	2	5	5	→	5	2	4	4	E	11646.860	0.005

6	2	4	5	→	5	2	3	4	A	11869.494	-0.007
6	2	4	7	→	5	2	3	6	A	11869.494	-0.002
6	2	4	5	→	5	2	3	4	E	11861.840	-0.029
6	2	4	7	→	5	2	3	6	E	11861.840	-0.002
6	1	5	7	→	5	1	4	6	A	12174.698	0.003
6	1	5	5	→	5	1	4	4	A	12174.698	0.017
6	1	5	7	→	5	1	4	6	E	12174.508	0.030
6	1	5	5	→	5	1	4	4	E	12174.508	0.017
7	1	7	8	→	6	1	6	7	A	12877.519	-0.025
7	1	7	6	→	6	1	6	5	A	12877.519	0.024
7	1	7	7	→	6	1	6	6	A	12877.519	0.035
7	2	6	8	→	6	2	5	7	A	13561.833	0.001
7	2	6	6	→	6	2	5	5	A	13561.833	0.006
7	2	6	8	→	6	2	5	7	E	13565.439	0.011
7	2	6	6	→	6	2	5	5	E	13565.439	0.006
7	3	5	7	→	6	3	4	6	A	13661.554	-0.004
7	3	5	8	→	6	3	4	7	A	13661.736	0.017
7	3	5	6	→	6	3	4	5	A	13661.736	0.002
7	3	5	7	→	6	3	4	6	E	13670.253	-0.001
7	3	5	8	→	6	3	4	7	E	13670.415	-0.002
7	3	5	6	→	6	3	4	5	E	13670.415	-0.017
7	3	4	7	→	6	3	3	6	A	13681.025	-0.010
7	3	4	8	→	6	3	3	7	A	13681.198	0.011
7	3	4	6	→	6	3	3	5	A	13681.198	-0.004
7	3	4	7	→	6	3	3	6	E	13672.225	-0.011
7	3	4	8	→	6	3	3	7	E	13672.408	0.020
7	3	4	6	→	6	3	3	5	E	13672.408	0.005
7	2	5	8	→	6	2	4	7	A	13918.510	0.014
7	2	5	6	→	6	2	4	5	A	13918.510	0.008
7	2	5	8	→	6	2	4	7	E	13914.844	0.018
7	2	5	6	→	6	2	4	5	E	13914.844	0.008
7	1	6	6	→	6	1	5	5	A	14172.901	0.003
7	1	6	7	→	6	1	5	6	A	14172.901	0.021
7	1	6	8	→	6	1	5	7	A	14172.901	-0.014
7	1	6	6	→	6	1	5	5	E	14172.695	0.008
7	1	6	7	→	6	1	5	6	E	14172.695	0.021
7	1	6	8	→	6	1	5	7	E	14172.695	-0.014
8	1	8	8	→	7	1	7	7	A	14690.608	0.010
8	1	8	7	→	7	1	7	6	A	14690.608	-0.009

8	1	8	9	→	7	1	7	8	A	14690.608	-0.027
8	0	8	8	→	7	0	7	7	A	15048.529	-0.002
8	0	8	7	→	7	0	7	6	A	15048.529	-0.018
8	0	8	9	→	7	0	7	8	A	15048.529	-0.034
8	0	8	8	→	7	0	7	7	E	15048.311	-0.010
8	0	8	7	→	7	0	7	6	E	15048.311	-0.018
8	0	8	9	→	7	0	7	8	E	15048.311	-0.034
8	3	6	9	→	7	3	5	8	A	15622.643	0.020
8	3	6	7	→	7	3	5	6	A	15622.643	-0.005
8	2	6	8	→	7	2	5	7	A	15987.622	0.026
8	2	6	7	→	7	2	5	6	A	15987.622	-0.025
8	2	6	9	→	7	2	5	8	A	15987.622	-0.033
8	2	6	8	→	7	2	5	7	E	15985.748	0.010
8	2	6	7	→	7	2	5	6	E	15985.748	-0.024
8	2	6	9	→	7	2	5	8	E	15985.748	-0.033
8	1	7	7	→	7	1	6	6	A	16154.635	-0.025
8	1	7	9	→	7	1	6	8	A	16154.635	-0.011
8	1	7	8	→	7	1	6	7	A	16154.635	0.018
8	1	7	7	→	7	1	6	6	E	16154.427	-0.005
8	1	7	9	→	7	1	6	8	E	16154.427	-0.011
8	1	7	8	→	7	1	6	7	E	16154.427	0.018
9	2	7	8	→	8	2	6	7	A	18070.201	-0.016
9	2	7	10	→	8	2	6	9	A	18070.201	-0.008
9	2	7	9	→	8	2	6	8	A	18070.201	0.012
9	2	7	8	→	8	2	6	7	E	18069.171	-0.034
9	2	7	10	→	8	2	6	9	E	18069.171	-0.008
9	2	7	9	→	8	2	6	8	E	18069.171	0.011
9	1	8	9	→	8	1	7	8	A	18116.148	-0.017
9	1	8	8	→	8	1	7	7	A	18116.148	-0.018
9	1	8	10	→	8	1	7	9	A	18116.148	-0.027
9	1	8	9	→	8	1	7	8	E	18115.922	0.019
9	1	8	8	→	8	1	7	7	E	18115.922	-0.018
10	1	10	9	→	9	1	9	8	A	18292.921	0.017
10	1	10	11	→	9	1	9	10	A	18292.921	-0.012
10	1	10	10	→	9	1	9	9	A	18292.921	0.007

Table S19: Observed transition frequencies of *A*- and *E*- species transitions of 5-MT···HOD. ν_{obs} - ν_{calc} values obtained after fitting using the XIAM program.

J'	K_{-1}'	K_1'	F'		J''	K_{-1}''	K_1''	F''	Symmetry	ν_{obs} (MHz)	ν_{obs} - ν_{calc} (MHz)
5	1	5	6	→	4	1	4	5	A	9054.737	0.045
5	1	5	6	→	4	1	4	5	E	9054.838	-0.012
5	0	5	4	→	4	0	4	3	A	9407.483	0.006
5	0	5	6	→	4	0	4	5	A	9407.483	-0.045
5	0	5	5	→	4	0	4	4	A	9407.483	0.036
5	2	4	6	→	4	2	3	5	A	9516.271	0.034
5	2	4	4	→	4	2	3	3	A	9516.271	-0.020
5	2	3	5	→	4	2	2	4	A	9638.941	0.004
5	2	3	6	→	4	2	2	5	A	9639.125	0.007
5	2	3	4	→	4	2	2	3	A	9639.125	-0.011
5	2	3	5	→	4	2	2	4	E	9623.874	-0.003
5	2	3	6	→	4	2	2	5	E	9624.074	0.027
5	2	3	4	→	4	2	2	3	E	9624.074	0.010
6	0	6	6	→	5	0	5	5	A	11225.372	0.028
6	0	6	5	→	5	0	5	4	A	11225.372	-0.006
6	0	6	7	→	5	0	5	6	A	11225.372	-0.029
6	0	6	6	→	5	0	5	5	E	11225.250	0.028
6	0	6	5	→	5	0	5	4	E	11225.250	-0.006
6	0	6	7	→	5	0	5	6	E	11225.250	-0.029
6	2	5	6	→	5	2	4	5	A	11407.837	-0.008
6	2	5	7	→	5	2	4	6	A	11407.975	0.018
6	2	5	5	→	5	2	4	4	A	11407.975	0.018
6	2	5	6	→	5	2	4	5	E	11415.900	-0.017
6	2	5	7	→	5	2	4	6	E	11416.027	0.005
6	2	5	5	→	5	2	4	4	E	11416.027	0.004
6	2	4	7	→	5	2	3	6	A	11618.931	-0.023
6	2	4	5	→	5	2	3	4	A	11618.931	0.002
6	2	4	7	→	5	2	3	6	E	11610.790	-0.027
6	2	4	5	→	5	2	3	4	E	11610.790	0.002
6	1	5	7	→	5	1	4	6	A	11921.993	-0.047
6	1	5	6	→	5	1	4	5	A	11921.993	0.042
6	1	5	5	→	5	1	4	4	A	11921.993	0.016
7	1	7	8	→	6	1	6	7	A	12638.883	-0.032
7	1	7	7	→	6	1	6	6	A	12638.883	0.033
7	1	7	6	→	6	1	6	5	A	12638.883	0.023
7	2	6	6	→	6	2	5	5	A	13293.475	0.003
7	2	6	8	→	6	2	5	7	A	13293.475	-0.006
7	2	6	6	→	6	2	5	5	E	13297.367	-0.007
7	2	6	8	→	6	2	5	7	E	13297.367	-0.005
8	1	8	9	→	7	1	7	8	A	14419.913	-0.010
8	1	8	8	→	7	1	7	7	A	14419.913	0.026
8	1	8	7	→	7	1	7	6	A	14419.913	0.018
8	2	7	9	→	7	2	6	8	A	15171.807	0.012
8	2	7	7	→	7	2	6	6	A	15171.807	0.007

8	2	6	9	→	7	2	5	8	A	15643.028	0.003
8	2	6	8	→	7	2	5	7	A	15643.028	0.030
8	2	6	7	→	7	2	5	6	A	15643.028	0.008
8	2	6	9	→	7	2	5	8	E	15641.019	0.012
8	2	6	8	→	7	2	5	7	E	15641.019	0.030
8	2	6	7	→	7	2	5	6	E	15641.019	0.020
9	1	9	10	→	8	1	8	9	A	16193.400	0.025
9	1	9	8	→	8	1	8	7	A	16193.400	0.014
9	1	9	9	→	8	1	8	8	A	16193.400	0.022
9	1	9	10	→	8	1	8	9	E	16193.288	-0.037
9	1	9	8	→	8	1	8	7	E	16193.288	0.014
9	1	9	9	→	8	1	8	8	E	16193.288	0.022
9	2	7	10	→	8	2	6	9	A	17678.809	0.009
9	2	7	8	→	8	2	6	7	A	17678.809	0.008
9	2	7	9	→	8	2	6	8	A	17678.809	0.017

Table S20: Observed transition frequencies of *A*- and *E*- species transitions of 5-MT···D_2O. ν_{obs} - ν_{calc} values obtained after fitting using the XIAM program.

J'	K_{-1}'	K_1'	*F'*		*J"*	K_{-1}"	K_1"	*F"*	Symmetry	ν_{obs} (MHz)	ν_{obs} - ν_{calc} (MHz)
4	0	4	3	→	3	0	3	2	A	7444.868	0.002
4	0	4	4	→	3	0	3	3	A	7444.868	-0.052
4	1	3	4	→	3	1	2	3	A	7841.849	-0.034
4	1	3	5	→	3	1	2	4	A	7841.985	0.022
4	1	3	3	→	3	1	2	2	A	7841.985	0.028
4	1	3	4	→	3	1	2	3	E	7841.400	-0.039
4	1	3	5	→	3	1	2	4	E	7841.536	0.023
4	1	3	3	→	3	1	2	2	E	7841.536	0.028
5	2	4	5	→	4	2	3	4	A	9366.167	0.007
5	2	4	4	→	4	2	3	3	A	9366.376	-0.018
5	2	4	6	→	4	2	3	5	A	9366.376	0.003
5	2	4	5	→	4	2	3	4	E	9381.225	-0.012
5	2	4	4	→	4	2	3	3	E	9381.475	0.021
5	2	4	6	→	4	2	3	5	E	9381.475	0.043
5	2	3	6	→	4	2	2	5	A	9483.276	-0.006
5	2	3	4	→	4	2	2	3	A	9483.276	-0.020
5	1	4	5	→	4	1	3	4	A	9790.386	0.031
5	1	4	4	→	4	1	3	3	A	9790.386	-0.037
5	1	4	6	→	4	1	3	5	A	9790.386	-0.064
5	1	4	5	→	4	1	3	4	E	9790.116	0.033
5	1	4	4	→	4	1	3	3	E	9790.116	-0.037
5	1	4	6	→	4	1	3	5	E	9790.116	-0.063
6	2	5	6	→	5	2	4	5	A	11228.552	0.002
6	2	5	5	→	5	2	4	4	A	11228.695	0.020
6	2	5	7	→	5	2	4	6	A	11228.695	0.020
6	2	5	6	→	5	2	4	5	E	11236.841	0.001
6	2	5	5	→	5	2	4	4	E	11236.989	0.024
6	2	5	7	→	5	2	4	6	E	11236.989	0.024
6	2	4	7	→	5	2	3	6	A	11429.555	-0.044
6	2	4	5	→	5	2	3	4	A	11429.555	0.001
6	2	4	7	→	5	2	3	6	E	11421.210	-0.028
6	2	4	5	→	5	2	3	4	E	11421.210	0.001
6	1	5	7	→	5	1	4	6	A	11730.093	-0.006
6	1	5	5	→	5	1	4	4	A	11730.093	0.021
6	1	5	6	→	5	1	4	5	A	11730.093	0.041
7	1	7	7	→	6	1	6	6	A	12448.144	0.006
7	1	7	6	→	6	1	6	5	A	12448.144	-0.008
7	1	7	8	→	6	1	6	7	A	12448.144	-0.030
7	0	7	8	→	6	0	6	7	A	12819.088	-0.009
7	0	7	6	→	6	0	6	5	A	12819.088	0.023
7	0	7	8	→	6	0	6	7	A	12819.088	0.000
7	0	7	8	→	6	0	6	7	E	12818.975	0.033
7	0	7	6	→	6	0	6	5	E	12818.975	0.023
7	0	7	8	→	6	0	6	7	E	12818.975	0.000
7	2	6	8	→	6	2	5	7	A	13085.078	0.012
7	2	6	6	→	6	2	5	5	A	13085.078	0.006

7	2	6	8	→	6	2	5	7	E	13089.108	0.002
7	2	6	6	→	6	2	5	5	E	13089.108	0.006
7	3	4	7	→	6	3	3	6	A	13187.907	0.022
7	3	4	8	→	6	3	3	7	A	13188.050	-0.020
7	3	4	6	→	6	3	3	5	A	13188.050	-0.035
7	3	4	7	→	6	3	3	6	E	13180.563	-0.015
7	3	4	8	→	6	3	3	7	E	13180.770	0.044
7	3	4	6	→	6	3	3	5	E	13180.770	0.029
7	2	5	6	→	6	2	4	5	A	13397.548	0.016
7	2	5	8	→	6	2	4	7	A	13397.548	-0.007
7	2	5	6	→	6	2	4	5	E	13393.440	0.022
7	2	5	8	→	6	2	4	7	E	13393.440	-0.007
7	1	6	7	→	6	1	5	6	A	13658.560	0.016
7	1	6	8	→	6	1	5	7	A	13658.560	-0.029
7	1	6	6	→	6	1	5	5	A	13658.560	-0.013
7	1	6	7	→	6	1	5	6	E	13658.356	0.014
7	1	6	8	→	6	1	5	7	E	13658.356	-0.029
7	1	6	6	→	6	1	5	5	E	13658.356	-0.013
8	1	8	7	→	7	1	7	6	A	14202.967	0.005
8	1	8	8	→	7	1	7	7	A	14202.967	0.006
8	1	8	9	→	7	1	7	8	A	14202.967	-0.017
8	0	8	9	→	7	0	7	8	A	14556.897	-0.020
8	0	8	8	→	7	0	7	7	A	14556.897	0.020
8	0	8	7	→	7	0	7	6	A	14556.897	0.017
8	0	8	9	→	7	0	7	8	E	14556.725	-0.002
8	0	8	8	→	7	0	7	7	E	14556.725	0.020
8	0	8	7	→	7	0	7	6	E	14556.725	0.017
8	2	6	9	→	7	2	5	8	A	15384.669	-0.016
8	2	6	8	→	7	2	5	7	A	15384.669	0.044
8	2	6	7	→	7	2	5	6	A	15384.669	0.008
8	2	6	9	→	7	2	5	8	E	15382.596	0.014
8	2	6	8	→	7	2	5	7	E	15382.596	0.043
8	2	6	7	→	7	2	5	6	E	15382.596	0.008
9	0	9	10	→	8	0	8	9	A	16272.388	0.000
9	0	9	9	→	8	0	8	8	A	16272.388	0.018
9	0	9	8	→	8	0	8	7	A	16272.388	0.013
9	0	9	10	→	8	0	8	9	E	16272.159	-0.008
9	0	9	9	→	8	0	8	8	E	16272.159	0.018
9	0	9	8	→	8	0	8	7	E	16272.159	0.013
9	1	8	9	→	8	1	7	8	A	17470.204	0.005
9	1	8	8	→	8	1	7	7	A	17470.204	-0.009
9	1	8	10	→	8	1	7	9	A	17470.204	-0.019
9	1	8	9	→	8	1	7	8	E	17469.937	-0.021
9	1	8	8	→	8	1	7	7	E	17469.937	-0.009
9	1	8	10	→	8	1	7	9	E	17469.937	-0.019

Table S21- Inertial defects and planar moments of 4-MT···H_2O and 5-MT···H_2O calculated using rotational constants determined from global (XIAM) fits.

4-MT···H_2O					
Parameter	$H_2{}^{16}O$	$H_2{}^{18}O$	DOH	HOD	D_2O
Δ_0	−3.6540(23)	−3.6493(27)	−3.6453(32)	−3.859(4)	−3.861(4)
P_{aa}	398.3404(12)	421.4885(13)	403.7909(16)	412.7571(22)	418.1207(20)
P_{bb}	128.9856(12)	130.1384(13)	129.8368(16)	130.7168(22)	131.5055(20)
P_{cc}	1.8270(12)	1.8247(13)	1.8227(16)	1.9293(22)	1.9305(20)
5-MT···H_2O					
Parameter	$H_2{}^{16}O$	$H_2{}^{18}O$	DOH	HOD	D_2O
Δ_0	−3.2681(22)	−3.2541(26)	−3.2719(22)	−3.668(6)	−3.677(4)
P_{aa}	463.8443(11)	493.0632(13)	472.2396(11)	482.7168(31)	490.9310(21)
P_{bb}	102.3986(11)	102.4574(13)	102.9455(11)	102.8196(31)	103.3429(21)
P_{cc}	1.6340(11)	1.6270(13)	1.6360(11)	1.8339(31)	1.8387(21)

Table S22- Results of fits of structural parameters (4-MT···H_2O, fit 1) using Kisiel's STRFIT (ref 48).

Input data

```
========================
14
 1  0  0  0    0.0          0.0          0.0        31.97145876
 2  1  0  0    1.7107       0.0          0.0        12.0
 3  2  1  0    1.2941    114.7509     0.0        14.003074
 4  3  2  1    1.3742    111.4992     0.0298   12.0
 5  4  3  2    1.3581    114.1145     0.0134   12.0
 6  4  3  2    1.4918    119.251   -179.7923   12.0
 7  2  3  4    1.0791    123.6355  -179.7456    1.0078250
 8  5  4  3    1.076     127.8739   179.8613    1.0078250
 9  6  4  3    1.0899    110.3175    63.4495    1.0078250
10  6  4  3    1.0875    111.0347  -175.9852    1.0078250
11  6  4  3    1.0892    110.3868   -54.7483    1.0078250
12  3  2  1    1.9484    115.2041  -179.7485    1.0078250
13 12  3  2    0.9588    169.88         0.0       15.9949146
14 13  12  3   0.9588    104.5        180.0        1.0078250
NO OF PARAMETERS TO BE FITTED: 3
atom no., parameter no.       12 1
atom no., parameter no.       12 2
atom no., parameter no.       13 2
NO OF CONSTANTS TO BE FITTED:  15
constant,species,value        1 1 3863.381                H216O
constant,species,value        2 1 1262.9188
constant,species,value        3 1 958.3805
constant,species,value        1 2 3829.702                H218O
constant,species,value        2 2 1193.8656
constant,species,value        3 2 916.1610
constant,species,value        1 3 3838.532                DOH
constant,species,value        2 3 1245.9618
constant,species,value        3 3 947.0629
constant,species,value        1 4 3809.98                 HOD
constant,species,value        2 4 1218.7017
constant,species,value        3 4 929.9048
constant,species,value        1 5 3787.428               D2O
constant,species,value        2 5 1203.1367
constant,species,value        3 5 919.4958
========================
```

Final cycle of fit (includes results for atomic coordinates) of structural parameters of 4-MT···H_2O

```
after:   5 iterations,  ALAMDA=  0.10E-07

 FINAL RESULTS OF LEAST SQUARES FIT:

        R(12, 3) =    2.026452 +-  0.008728
      A(12, 3, 2) =   139.161138 +-  2.168593
      A(13,12, 3) =   169.289368 +-  7.094171

         Chi-squared =       8.7494357405
     Deviation of fit =       0.853885

Ni Axis    Iobs      Icalc     Io-c           Bobs      Bcalc     Bo-c
```

1	a	130.81263	131.39902	-0.58640	3863.3810	3846.1398	17.2412
1	b	400.16746	399.31897	0.84849	1262.9188	1265.6023	-2.6835
1	c	527.32606	527.62095	-0.29489	958.3805	957.8449	0.5356
2	a	131.96301	132.95597	-0.99296	3829.7020	3801.1006	28.6014
2	b	423.31315	422.61815	0.69500	1193.8656	1195.8289	-1.9633
2	c	551.62685	552.47707	-0.85021	916.1610	914.7511	1.4099
3	a	131.65945	131.83591	-0.17646	3838.5320	3833.3941	5.1379
3	b	405.61357	405.58962	0.02395	1245.9618	1246.0354	-0.0736
3	c	533.62771	534.32848	-0.70077	947.0629	945.8208	1.2421
4	a	132.64611	131.40790	1.23821	3809.9800	3845.8801	-35.9001
4	b	414.68639	414.56429	0.12210	1218.7017	1219.0606	-0.3589
4	c	543.47392	542.87514	0.59878	929.9048	930.9305	-1.0257
5	a	133.43594	131.84022	1.59572	3787.4280	3833.2689	-45.8409
5	b	420.05120	420.67341	-0.62222	1203.1367	1201.3571	1.7796
5	c	549.62623	549.41658	0.20965	919.4958	919.8467	-0.3509

Correlation coefficients:

		1	2	3
1:	R(12, 3)	1.000		
2:	A(12, 3, 2)	0.969	1.000	
3:	A(13,12, 3)	-0.971	-0.996	1.000

Final principal coordinates of parent:

ATOM NO.	A	B	C	MASS
1	-2.053723	-0.421936	0.001649	31.9714588
2	-0.561384	-1.258243	-0.001019	12.0000000
3	0.485791	-0.497904	-0.002611	14.0030740
4	0.142099	0.832623	-0.002425	12.0000000
5	-1.196860	1.059832	-0.000935	12.0000000
6	1.219994	1.863932	0.000602	12.0000000
7	-0.517197	-2.336431	-0.004838	1.0078250
8	-1.706039	2.007730	-0.002558	1.0078250
9	1.807308	1.794815	0.916116	1.0078250
10	0.802197	2.865442	-0.070667	1.0078250
11	1.903315	1.701036	-0.831801	1.0078250
12	2.504969	-0.669438	-0.000204	1.0078250
13	3.428599	-0.926735	0.001495	15.9949146
14	3.908964	-0.096951	-0.000843	1.0078250

Principal coordinates and estimated uncertainties:

ATOM NO.	A	dA	B	dB	C	dC
1	-2.05372	0.00128	-0.42194	0.00309	0.00165	0.00000
2	-0.56138	0.00314	-1.25824	0.00031	-0.00102	0.00001
3	0.48579	0.00144	-0.49790	0.00267	-0.00261	0.00001
4	0.14210	0.00169	0.83262	0.00189	-0.00242	0.00000
5	-1.19686	0.00220	1.05983	0.00115	-0.00094	0.00001
6	1.21999	0.00400	1.86393	0.00434	0.00060	0.00000

7	-0.51720	0.00557	-2.33643	0.00041	-0.00484	0.00002
8	-1.70604	0.00433	2.00773	0.00230	-0.00256	0.00001
9	1.80731	0.00384	1.79481	0.00567	0.91612	0.00000
10	0.80220	0.00627	2.86544	0.00339	-0.07067	0.00001
11	1.90331	0.00364	1.70104	0.00590	-0.83180	0.00001
12	2.50497	0.01568	-0.66944	0.07688	-0.00020	0.00024
13	3.42860	0.00681	-0.92674	0.00247	0.00150	0.00001
14	3.90896	0.06462	-0.09695	0.04341	-0.00084	0.00021

Table S23- Results of fits of structural parameters (4-MT···H_2O, fit 2) using Kisiel's STRFIT STRFIT (ref 48).

Input data

```
========================
14
1  0  0  0 0.0         0.0        0.0       31.97145876
 2  1  0  0 1.7107      0.0        0.0       12.0
 3  2  1  0 1.2941  114.7509     0.0       14.003074
 4  3  2  1 1.3742  111.4992     0.0298  12.0
 5  4  3  2 1.3581  114.1145     0.0134  12.0
 6  4  3  2 1.4918  119.251  -179.7923  12.0
 7  2  3  4 1.0791  123.6355 -179.7456   1.0078250
 8  5  4  3 1.076   127.8739  179.8613   1.0078250
 9  6  4  3 1.0899  110.3175    63.4495   1.0078250
10  6  4  3 1.0875  111.0347 -175.9852   1.0078250
11  6  4  3 1.0892  110.3868  -54.7483   1.0078250
12  3  2  1 1.9484  115.2041 -179.7485  1.0078250
13 12  3  2 0.9588  169.88      180.0      15.9949146
14 13 12  3 0.9588  104.5       180.0       1.0078250
NO OF PARAMETERS TO BE FITTED: 3
atom no., parameter no.      12 1
atom no., parameter no.      12 2
atom no., parameter no.      13 2
NO OF CONSTANTS TO BE FITTED:  15
constant,species,value       1 1 3863.381            H216O
constant,species,value       2 1 1262.9188
constant,species,value       3 1 958.3805
constant,species,value       1 2 3829.702            H218O
constant,species,value       2 2 1193.8656
constant,species,value       3 2 916.1610
constant,species,value       1 3 3838.532            DOH
constant,species,value       2 3 1245.9618
constant,species,value       3 3 947.0629
constant,species,value       1 4 3809.98             HOD
constant,species,value       2 4 1218.7017
constant,species,value       3 4 929.9048
constant,species,value       1 5 3787.428            D2O
constant,species,value       2 5 1203.1367
constant,species,value       3 5 919.4958
========================
after:   6 iterations,  ALAMDA=  0.10E-06

 FINAL RESULTS OF LEAST SQUARES FIT:

        R(12, 3) =    2.029556 +-  0.006827
      A(12, 3, 2) =  134.692707 +-  1.365994
      A(13,12, 3) =  167.419553 +-  4.347490

          Chi-squared =       3.2922056723
      Deviation of fit =      0.523785

Ni Axis    Iobs      Icalc    Io-c           Bobs      Bcalc    Bo-c
```

1 a	130.81263	130.27406	0.53857	3863.3810	3879.3527	-15.9717
1 b	400.16746	399.82279	0.34468	1262.9188	1264.0075	-1.0887
1 c	527.32606	526.99980	0.32626	958.3805	958.9738	-0.5933
2 a	131.96301	131.44225	0.52076	3829.7020	3844.8750	-15.1730
2 b	423.31315	423.23492	0.07823	1193.8656	1194.0863	-0.2207
2 c	551.62685	551.58012	0.04673	916.1610	916.2386	-0.0776
3 a	131.65945	130.97943	0.68002	3838.5320	3858.4609	-19.9289
3 b	405.61357	405.98426	-0.37069	1245.9618	1244.8242	1.1376
3 c	533.62771	533.86664	-0.23893	947.0629	946.6390	0.4239
4 a	132.64611	133.07306	-0.42696	3809.9800	3797.7559	12.2241
4 b	414.68639	413.81464	0.87175	1218.7017	1221.2690	-2.5673
4 c	543.47392	543.79062	-0.31670	929.9048	929.3632	0.5416
5 a	133.43594	133.66689	-0.23096	3787.4280	3780.8839	6.5441
5 b	420.05120	419.90722	0.14398	1203.1367	1203.5492	-0.4125
5 c	549.62623	550.47704	-0.85081	919.4958	918.0746	1.4212

Correlation coefficients:

		1	2	3
1:	R(12, 3)	1.000		
2:	A(12, 3, 2)	-0.977	1.000	
3:	A(13,12, 3)	-0.982	0.996	1.000

Final principal coordinates of parent:

ATOM NO.	A	B	C	MASS
1	-2.057436	-0.413468	0.001643	31.9714588
2	-0.571051	-1.260312	-0.000987	12.0000000
3	0.481477	-0.507401	-0.002575	14.0030740
4	0.147207	0.825524	-0.002417	12.0000000
5	-1.190111	1.062201	-0.000952	12.0000000
6	1.232372	1.849182	0.000610	12.0000000
7	-0.534493	-2.338786	-0.004788	1.0078250
8	-1.692571	2.013677	-0.002599	1.0078250
9	1.819168	1.775926	0.916135	1.0078250
10	0.821672	2.853621	-0.070682	1.0078250
11	1.914536	1.681441	-0.831779	1.0078250
12	2.481840	-0.850390	0.000381	1.0078250
13	3.439453	-0.802703	0.001083	15.9949146
14	3.725385	-1.717869	0.004422	1.0078250

Principal coordinates and estimated uncertainties:

ATOM NO.	A	dA	B	dB	C	dC
1	-2.05744	0.00073	-0.41347	0.00184	0.00164	0.00000
2	-0.57105	0.00184	-1.26031	0.00019	-0.00099	0.00001
3	0.48148	0.00085	-0.50740	0.00160	-0.00257	0.00001
4	0.14721	0.00104	0.82552	0.00115	-0.00242	0.00000
5	-1.19011	0.00135	1.06220	0.00067	-0.00095	0.00000
6	1.23237	0.00240	1.84918	0.00261	0.00061	0.00000
7	-0.53449	0.00329	-2.33879	0.00024	-0.00479	0.00001

8	-1.69257	0.00262	2.01368	0.00135	-0.00260	0.00001
9	1.81917	0.00230	1.77593	0.00340	0.91614	0.00000
10	0.82167	0.00375	2.85362	0.00206	-0.07068	0.00000
11	1.91454	0.00217	1.68144	0.00353	-0.83178	0.00000
12	2.48184	0.00181	-0.85039	0.04841	0.00038	0.00016
13	3.43945	0.00400	-0.80270	0.00169	0.00108	0.00001
14	3.72539	0.04398	-1.71787	0.01650	0.00442	0.00001

Table S24- Results of fits of structural parameters (5-MT···H_2O) using Kisiel's STRFIT (ref 48).

Input data

```
========================
14
1  0  0  0 0.0          0.0          0.0        31.97145876
 2  1  0  0 1.7152       0.0          0.0        12.0
 3  2  1  0 1.2932     114.5153      0.0        14.003074
 4  3  2  1 1.3684     110.8122     -0.0077    12.0
 5  4  3  2 1.3567     116.3445      0.0159    12.0
 6  5  4  3 1.4919     128.4322   179.492      12.0
 7  2  3  4 1.0793     123.5557 -179.7425     1.0078250
 8  4  3  2 1.0797     119.2984  179.8692     1.0078250
 9  6  5  4 1.0896     111.7245   123.1951    1.0078250
10  6  5  4 1.0898     111.5425  -115.9325    1.0078250
11  6  5  4 1.0884     109.4425      3.5926    1.0078250
12  3  2  1 1.9484     115.2041  -179.7485    1.0078250
13 12  3  2 0.9588     169.88        0.0     15.9949146
14 13 12  3 0.9588     104.5       180.0       1.0078250
NO OF PARAMETERS TO BE FITTED: 3
atom no., parameter no.        12 1
                          12 2
                          13 2
NO OF CONSTANTS TO BE FITTED:  15
constant,species,value         1 1 4857.887          H216O
constant,species,value         2 1 1085.7196
constant,species,value         3 1 892.5126
constant,species,value         1 2 4855.47           H218O
constant,species,value         2 2 1021.60693
constant,species,value         3 2 848.6339
constant,species,value         1 3 4832.393          DOH
constant,species,value         2 3 1066.48040
constant,species,value         3 3 878.6371
constant,species,value         1 4 4829.07           HOD
constant,species,value         2 4 1042.9847
constant,species,value         3 4 863.1044
constant,species,value         1 5 4804.82           D2O
constant,species,value         2 5 1025.5885
constant,species,value         3 5 850.4142
========================
after:   5 iterations,  ALAMDA=  0.10E-07

 FINAL RESULTS OF LEAST SQUARES FIT:

        R(12, 3) =     2.003654 +-  0.004168
      A(12, 3, 2) =    99.399330 +-  0.779430
      A(13,12, 3) =   166.046538 +-  2.302674

          Chi-squared =       1.0646333679
       Deviation of fit =       0.297858

Ni Axis    Iobs       Icalc     Io-c           Bobs       Bcalc     Bo-c

 1  a   104.03268   103.88975   0.14293       4857.8870   4864.5705   -6.6835
 1  b   465.47839   465.26494   0.21345       1085.7196   1086.2177   -0.4981
```

1 c	566.24300	566.02381	0.21919	892.5126	892.8582	-0.3456
2 a	104.08447	103.97553	0.10894	4855.4700	4860.5574	-5.0874
2 b	494.69027	494.85507	-0.16480	1021.6069	1021.2667	0.3402
2 c	595.52065	595.69971	-0.17906	848.6339	848.3788	0.2551
3 a	104.58152	104.09867	0.48286	4832.3930	4854.8078	-22.4148
3 b	473.87557	473.94048	-0.06491	1066.4804	1066.3343	0.1461
3 c	575.18515	574.90827	0.27688	878.6371	879.0603	-0.4232
4 a	104.65349	104.94021	-0.28673	4829.0700	4815.8756	13.1944
4 b	484.55074	484.15455	0.39620	1042.9847	1043.8382	-0.8535
4 c	585.53636	585.96387	-0.42751	863.1044	862.4747	0.6297
5 a	105.18167	105.11157	0.07011	4804.8200	4808.0247	-3.2047
5 b	492.76977	492.64354	0.12623	1025.5885	1025.8513	-0.2628
5 c	594.27395	594.62422	-0.35027	850.4142	849.9133	0.5009

Correlation coefficients:

		1	2	3
1:	R(12, 3)	1.000		
2:	A(12, 3, 2)	0.840	1.000	
3:	A(13,12, 3)	-0.948	-0.964	1.000

Final principal coordinates of parent:

ATOM NO.	A	B	C	MASS
1	-0.949788	-1.197935	0.001162	31.9714588
2	0.713001	-0.777192	-0.003278	12.0000000
3	0.944576	0.495105	-0.002821	14.0030740
4	-0.226790	1.202496	0.001367	12.0000000
5	-1.370664	0.472989	0.003828	12.0000000
6	-2.780940	0.959663	-0.001910	12.0000000
7	1.491040	-1.525185	-0.010355	1.0078250
8	-0.192327	2.281645	0.000095	1.0078250
9	-3.344569	0.572737	0.846523	1.0078250
10	-3.302108	0.665400	-0.912656	1.0078250
11	-2.789365	2.046515	0.055509	1.0078250
12	2.947972	0.463060	0.000030	1.0078250
13	3.874662	0.217010	0.001325	15.9949146
14	4.344900	1.052577	0.002078	1.0078250

Principal coordinates and estimated uncertainties:

ATOM NO.	A	dA	B	dB	C	dC
1	-0.94979	0.00239	-1.19794	0.00030	0.00116	0.00000
2	0.71300	0.00153	-0.77719	0.00311	-0.00328	0.00000
3	0.94458	0.00109	0.49510	0.00358	-0.00282	0.00000
4	-0.22679	0.00254	1.20250	0.00118	0.00137	0.00000
5	-1.37066	0.00105	0.47299	0.00116	0.00383	0.00000
6	-2.78094	0.00204	0.95966	0.00405	-0.00191	0.00000
7	1.49104	0.00306	-1.52518	0.00470	-0.01035	0.00000
8	-0.19233	0.00475	2.28165	0.00126	0.00009	0.00001
9	-3.34457	0.00125	0.57274	0.00520	0.84652	0.00000

10	-3.30211	0.00144	0.66540	0.00511	-0.91266	0.00000
11	-2.78936	0.00426	2.04651	0.00406	0.05551	0.00001
12	2.94797	0.00465	0.46306	0.02502	0.00003	0.00001
13	3.87466	0.00222	0.21701	0.00124	0.00133	0.00000
14	4.34490	0.02079	1.05258	0.01338	0.00208	0.00003

Table S25- Atomic coordinates of 4-MT···H_2O and 5-MT···H_2O determined by the r_0 method. Fit 1 assumes ∠(O–H_b···N3–C2) = 0° and ∠(H_{nb}–O–H_b···N3) = 180°. Fit 2 assumes ∠(O–H_b···N3–C2) = 180 ° and ∠(H_{nb}–O–H_b···N3) = 180°

4-MT···H_2O (Fit 1)			
	a /Å	*b* /Å	*c* /Å
S(1)	–2.05372(128)	0.42194(309)	0.00165(0)
C(2)	–0.56138(314)	1.25824(31)	–0.00102(1)
H(2)	–0.51720(557)	2.33643(41)	–0.00484(2)
N(3)	0.48579(144)	0.49790(267)	–0.00261(1)
C(4)	0.14210(169)	–0.83262(189)	–0.00242(0)
C(5)	–1.19686(220)	–1.05983(115)	–0.00094(1)
H(5)	–1.70604(433)	–2.00773(230)	–0.00256(1)
C(6)	1.21999(400)	–1.86393(434)	0.00060(0)
H(6)	1.80731(384)	–1.79481(567)	0.91612(0)
H(6)	0.80220(627)	–2.86544(339)	0.07067(1)
H(6)	1.90331(364)	–1.70104(590)	–0.83180(1)
H_b	2.50497(1568)	0.66944(7688)	–0.00020(24)
O	3.42860(681)	0.92674(247)	– 0.00150(1)
H_{nb}	3.90896(6464)	0.09695(4341)	–0.00084(21)
4-MT···H_2O (Fit 2)			
	a /Å	*b* /Å	*c* /Å
S(1)	–2.05744(73)	0.41347(184)	0.00164(0)
C(2)	–0.57105(184)	1.26031(19)	–0.00099(1)
H(2)	–0.53449(329)	2.33879(24)	–0.00479(1)
N(3)	0.48148(85)	0.50740(160)	–0.00257(1)
C(4)	0.14721(104)	–0.82552(115)	–0.00242(0)
C(5)	–1.19011(135)	–1.06220(67)	–0.00095(0)
H(5)	–1.69257(262)	–2.01368(135)	–0.00260(1)
C(6)	1.23237(240)	–1.84918(261)	0.00061(0)
H(6)	1.81917(230)	–1.77593(340)	0.91614(0)
H(6)	0.82167(375)	–2.85362(206)	–0.07068(0)
H(6)	1.91454(217)	–1.68144(353)	–0.83178(0)
H_b	2.48184(181)	0.85039(4841)	0.00038(16)
O	3.43945(400)	0.80270(169)	0.00108(1)
H_{nb}	3.72539(4398)	1.71787(1650)	0.00442(1)
5-MT···H_2O			
	a /Å	*b* /Å	*c* /Å
S(1)	–0.94979(239)	–1.19794(30)	0.00116(0)
C(2)	0.71300(153)	–0.77719(311)	–0.00328(0)
H(2)	1.49104(306)	–1.52518(470)	–0.01035(0)
N(3)	0.94458(109)	0.49510(358)	–0.00282(0)
C(4)	–0.22679(254)	1.20250(118)	0.00137(0)
H(4)	–0.19233(475)	2.28165(126)	0.00009(1)
C(5)	–1.37066(105)	0.47299(116)	0.00383(0)
C(6)	–2.78094(204)	0.95966(405)	–0.00191(0)
H(6)	–3.34457(125)	0.57274(520)	0.84652(0)
H(6)	–3.30211(144)	0.66540(511)	–0.91266(0)
H(6)	–2.78936(426)	2.04651(406)	0.05551(1)
H_b	2.94797(465)	0.46306(2502)	0.00003(1)
O	3.87466(222)	0.21701(124)	0.00133(0)
H_{nb}	4.34490(2079)	1.05258(1338)	0.00208(3)

Table S26- Comparison of DFT calculated and experimentally determined (r_s and r_0) atomic coordinates of H_2O in 4-MT···H_2O and 5-MT···H_2O

	Method	a / Å	b / Å	c / Å
		4-MT···H_2O		
H_b	r_e (calc.)	2.426683[a]	0.825440	–0.006987
	r_s (exp.)	2.33263(77)[b]	0.9330(19)	[0][c]
	r_0: fit 1 (exp.)[d]	2.50497(1568)	0.66944(7688)	–0.00020(24)
	r_0: fit 2 (exp.)[d]	2.48184(181)	0.85039(4841)	0.00038(16)
O	r_e (calc.)	3.381957	0.878949	–0.162564
	r_s (exp.)	3.42010(46)	0.7971(20)	[0]
	r_0: fit 1 (exp.)	3.42860(681)	0.92674(247)	– 0.00150(1)
	r_0: fit 2 (exp.)	3.43945(400)	0.80270(169)	0.00108(1)
H_{nb}	r_e (calc.)	3.754232	1.191681	0.659280
	r_s (exp.)	3.78858(51)	1.3515(15)	0.3282(61)
	r_0: fit 1 (exp.)	3.90896(6464)	0.09695(4341)	–0.00084(21)
	r_0: fit 2 (exp.)	3.72539(4398)	1.71787(1650)	0.00442(1)
		5-MT···H_2O		
	Method	a / Å	b / Å	c / Å
H_b	r_e (calc.)	2.944962	0.431497	0.028953
	r_s (exp.)	2.89860(58)	0.7489(23)	0.046(38)
	r_0 (exp.)	2.94797(465)	0.46306(2502)	0.00003(1)
O	r_e (calc.)	3.903074	0.315986	0.115239
	r_s (exp.)	3.85045(41)	0.1796(87)	[0]
	r_0 (exp.)	3.87466(222)	0.21701(124)	0.00133(0)
H_{nb}	r_e (calc.)	4.269998	0.647495	–0.701634
	r_s (exp.)	4.34578(51)	0.6656(34)	–0.4576(50)
	r_0 (exp.)	4.34490(2079)	1.05258(1338)	0.00208(3)

[a] r_e geometries calculated at the ωB97X-D/aug-cc-pVQZ level of theory

[b] Numbers in parentheses are Costain errors for r_s results and one standard deviation in units of the last significant figure for r_0 results

[c] Imaginary values obtained for r_s coordinates are indicated in square brackets and assumed to be zero

[d] Fit 1 assumes ∠(O–H_b···N3–C2) = 0 ° and ∠(H_{nb}–O–H_b···N3) = 180 °. Fit 2 assumes ∠(O–H_b···N3–C2) = 180 ° and ∠(H_{nb}–O–H_b···N3) = 180 °

Table S27. Results of a new fit of structural parameters of thiazole···H_2O (Ref 25) using Kisiel's STRFIT (ref 48).

The original fit of geometrical parameters of Ref 25 was performed to 3 experimentally-determined moments of inertia for each of 7 isotopologues of thiazole···H_2O (where isotopic substitutions were available at each of S, C2, N, C4, C5 of thiazole; and O of H_2O).

A new fit of the data from Ref. 25 to determine revised geometrical parameters for thiazole···H_2O is presented. The initial assumptions about the molecular geometry (the values of geometrical parameters used within the z-matrix) and the number of parameters determined by the fit are unchanged from Ref 25. The new fit is performed to the 3 experimentally-determined moments of inertia for each of the 7 isotopologues listed above and also the 3 experimentally-determined moments of inertia for each of D_2O, HOD and DOH (amounting to a total of 30 experimentally-determined moments of inertia). The experimentally-determined results used are those which were originally presented in Ref 25. The parameters and coordinates determined by the new r_0 fit are consistent with the r_s and r_e geometries presented in Ref 25.

The inclusion of the results for the deuterium-containing isotopologues in the new fit yields more reliable and accurate r_0 values for the $r(H_b···N3)$, $\angle(H_b···N3–C2)$ and $\angle(O–H_b···N3)$ parameters than was achieved previously. The results of the new fit are included in Table 4 and referred to within the discussion of the present work.

```
Input data
=========================
11
 1  0  0  0   0.000000    0.000000    0.000000    31.9720700
 2  1  0  0   1.723108    0.000000    0.000000    12.0000000
 3  2  1  0   1.301222  114.515541    0.000000    14.0030744
 4  3  2  1   1.370566  111.142830    0.000000    12.0000000
 5  4  3  2   1.361370  115.214000    0.000000    12.0000000
 6  5  4  3   1.076695  128.557000  180.000000     1.0078252
 7  4  3  2   1.079538  119.486717  180.000000     1.0078252
 8  2  1  5   1.079922  122.046783  180.000000     1.0078252
 9  3  2  5   2.897063   96.586000  180.000000    15.9949150
10  9  3  1   0.974476   13.614087  180.000000     1.0078250
11  9 10  3   0.961762  105.303000  180.000000     1.0078250
NO OF PARAMETERS TO BE FITTED: 11
atom no., parameter no.   FIX 2 1 0          C2-S1
atom no., parameter no.   FIX 3 1 0          N3-C2
atom no., parameter no.   FIX 4 1 0          C4-N3
atom no., parameter no.   FIX 5 1 0          C5-S1
atom no., parameter no.       9 1 0          O9-N3
atom no., parameter no.   FIX 3 2 0          N3-C2-S1
atom no., parameter no.   FIX 4 2 0          C4-N3-C2
atom no., parameter no.   FIX 5 2 0          C5-S1-C2
atom no., parameter no.       9 2 0          O9-N3-C2
atom no., parameter no.      10 2 0          H10-O9-N3
atom no., parameter no.   FIX11 3 0          H11-O9-H10-N3
!
! parameters
!
NO OF CONSTANTS TO BE FITTED:  30
constant,species,value      1 1 6435.2234          parent
constant,species,value      2 1 1513.00819
```

constant,species,value 3 1 1225.07882
constant,species,value 1 2 6327.748 34S1 atom numbering from paper
constant,species,value 2 2 1493.7502
constant,species,value 3 2 1208.5412
constant,species,value 1 3 6399.573 13C2
constant,species,value 2 3 1512.96825
constant,species,value 3 3 1223.76061
constant,species,value 1 4 6408.43 15N3
constant,species,value 2 4 1511.17055
constant,species,value 3 4 1222.92237
constant,species,value 1 5 6268.854 13C4
constant,species,value 2 5 1512.36278
constant,species,value 3 5 1218.46091
constant,species,value 1 6 6374.863 13C5
constant,species,value 2 6 1500.96105
constant,species,value 3 6 1214.98332
constant,species,value 1 7 6422.4245 18O10
constant,species,value 2 7 1414.51745
constant,species,value 3 7 1159.25831
constant,species,value 1 8 6412.9180 D2O
constant,species,value 2 8 1414.74304
constant,species,value 3 8 1160.01027
constant,species,value 1 9 6441.4602 DOH
constant,species,value 2 9 1481.23741
constant,species,value 3 9 1204.41989
constant,species,value 110 6407.3711 HOD
constant,species,value 210 1443.01445
constant,species,value 310 1178.65137

========================

Final cycle of fit (includes results for atomic coordinates) of structural parameters of thi···(H_2O).

after: 6 iterations, ALAMDA= 0.10E-08

FINAL RESULTS OF LEAST SQUARES FIT:

```
       R( 2, 1) = [   1.723108 ]   FIXED    C2-S1
       R( 3, 2) = [   1.301222 ]   FIXED    N3-C2
       R( 4, 3) = [   1.370566 ]   FIXED    C4-N3
       R( 5, 4) = [   1.361370 ]   FIXED    C5-S1
       R( 9, 3) =     2.939587 +-  0.002099   O9-N3
     A( 3, 2, 1) = [ 114.515541 ]   FIXED    N3-C2-S1
     A( 4, 3, 2) = [ 111.142830 ]   FIXED    C4-N3-C2
     A( 5, 4, 3) = [ 115.214000 ]   FIXED    C5-S1-C2
     A( 9, 3, 2) =    91.980010 +-  0.137139   O9-N3-C2
     A(10, 9, 3) =     7.408358 +-  1.074698   H10-O9-N3
   D(11, 9,10, 3) = [ 180.000000 ]   FIXED    H11-O9-H10-N3

        Chi-squared =      1.2055359648
    Deviation of fit =      0.211304
```

Ni	Axis	Iobs	Icalc	Io-c	Bobs	Bcalc	Bo-c
1	a	78.53325	78.51389	0.01936	6435.2234	6436.8099	-1.5865
1	b	334.02265	333.99382	0.02883	1513.0082	1513.1388	-0.1306
1	c	412.52775	412.50771	0.02004	1225.0788	1225.1383	-0.0595
2	a	79.86712	79.84674	0.02038	6327.7480	6329.3634	-1.6154
2	b	338.32900	338.33477	-0.00577	1493.7502	1493.7247	0.0255
2	c	418.17276	418.18151	-0.00875	1208.5412	1208.5159	0.0253
3	a	78.97074	78.94635	0.02439	6399.5730	6401.5500	-1.9770
3	b	334.03147	334.04617	-0.01470	1512.9682	1512.9017	0.0666
3	c	412.97212	412.99252	-0.02040	1223.7606	1223.7001	0.0605
4	a	78.86159	78.83775	0.02384	6408.4300	6410.3681	-1.9381
4	b	334.42884	334.41061	0.01823	1511.1706	1511.2529	-0.0824
4	c	413.25518	413.24836	0.00682	1222.9224	1222.9426	-0.0202
5	a	80.61745	80.60669	0.01075	6268.8540	6269.6903	-0.8363
5	b	334.16520	334.15650	0.00870	1512.3628	1512.4021	-0.0394
5	c	414.76834	414.76320	0.00514	1218.4609	1218.4760	-0.0151
6	a	79.27684	79.27413	0.00271	6374.8630	6375.0810	-0.2180
6	b	336.70361	336.66826	0.03536	1500.9610	1501.1187	-0.1576
6	c	415.95551	415.94239	0.01312	1214.9833	1215.0217	-0.0383
7	a	78.68975	78.65228	0.03747	6422.4245	6425.4844	-3.0599
7	b	357.28015	357.57467	-0.29452	1414.5175	1413.3524	1.1651
7	c	435.95030	436.22695	-0.27665	1159.2583	1158.5231	0.7352
8	a	78.80640	78.72580	0.08060	6412.9180	6419.4837	-6.5657
8	b	357.22318	356.95302	0.27017	1414.7430	1415.8138	-1.0708
8	c	435.66770	435.67882	-0.01112	1160.0103	1159.9807	0.0296
9	a	78.45721	78.52841	-0.07120	6441.4602	6435.6198	5.8404
9	b	341.18704	340.59444	0.59260	1481.2374	1483.8146	-2.5772
9	c	419.60367	419.12286	0.48082	1204.4199	1205.8016	-1.3817
10	a	78.87463	78.71603	0.15859	6407.3711	6420.2804	-12.9093
10	b	350.22450	350.54874	-0.32424	1443.0144	1441.6797	1.3347
10	c	428.77735	429.26477	-0.48742	1178.6514	1177.3130	1.3383

Correlation coefficients:

		1	2	3
1:	R(9, 3)	1.000		
2:	A(9, 3, 2)	-0.706	1.000	
3:	A(10, 9, 3)	-0.565	-0.157	1.000

Final principal coordinates of parent:

ATOM NO.	A	B	C	MASS
1	-1.485134	-0.832064	0.000000	31.9720700
2	0.229338	-0.659772	0.000000	12.0000000
3	0.648182	0.572198	0.000000	14.0030744
4	-0.402964	1.451710	0.000000	12.0000000
5	-1.638125	0.879253	0.000000	12.0000000

6	-2.601051	1.360963	0.000000	1.0078252
7	-0.207474	2.513400	0.000000	1.0078252
8	0.891012	-1.513247	0.000000	1.0078252
9	3.462351	-0.277287	0.000000	15.9949150
10	2.573549	0.122255	0.000000	1.0078250
11	4.074215	0.464743	0.000000	1.0078250

Principal coordinates and estimated uncertainties:

ATOM NO.	A	dA	B	dB	C	dC
1	-1.48513	0.00149	-0.83206	0.00133	0.00000	0.00000
2	0.22934	0.00119	-0.65977	0.00164	0.00000	0.00000
3	0.64818	0.00095	0.57220	0.00237	0.00000	0.00000
4	-0.40296	0.00247	1.45171	0.00055	0.00000	0.00000
5	-1.63812	0.00148	0.87925	0.00160	0.00000	0.00000
6	-2.60105	0.00232	1.36096	0.00326	0.00000	0.00000
7	-0.20747	0.00431	2.51340	0.00088	0.00000	0.00000
8	0.89101	0.00267	-1.51325	0.00279	0.00000	0.00000
9	3.46235	0.00142	-0.27729	0.00019	0.00000	0.00000
10	2.57355	0.00623	0.12226	0.01673	0.00000	0.00000
11	4.07421	0.01274	0.46474	0.01179	0.00000	0.00000
		|		|		|

NOTES: 1/ only the uncertainties for those coordinates which are completely
defined by the fitted internals should be trusted
2/ the uncertainties are somewhat limited by the linear approximation
coord=(d coord/d parameter)*parameter used for evaluation
3/ only the effect of the internals R, A, and D is propagated

---

Selected internal coordinates were determined from the *a*, *b*, *c* coordinates (as given above) using the EVAL program available from the ProSpe website as shown below.

```
=====================================================================
 ____________________________________________________________________
|                                                                    |
|     E V A L  -  Internals and their errors from Cartesians         |
|____________________________________________________________________|
 version 22.X.2012                        Zbigniew KISIEL


 WARNING:

        The EVAL uncertainties are evaluated by assuming that
        the correlation matrix is a unit matrix.

        The EVAL uncertainties may thus differ significantly
        (but typically by not more than 30% either way)
```

from uncertainties in explicitly fitted internals
corresponding to the input Cartesians.

!
! Thiazole_H2O test
!

INPUT CARTESIANS:

1S	-1.48513	0.00149	-0.83206	0.00133	0.00000	0.00000
2C	0.22934	0.00119	-0.65977	0.00164	0.00000	0.00000
3N	0.64818	0.00095	0.57220	0.00237	0.00000	0.00000
4C	-0.40296	0.00247	1.45171	0.00055	0.00000	0.00000
5C	-1.63812	0.00148	0.87925	0.00160	0.00000	0.00000
6H	-2.60105	0.00232	1.36096	0.00326	0.00000	0.00000
7H	-0.20747	0.00431	2.51340	0.00088	0.00000	0.00000
8H	0.89101	0.00267	-1.51325	0.00279	0.00000	0.00000
9O	3.46235	0.00142	-0.27729	0.00019	0.00000	0.00000
10H	2.57355	0.00623	0.12226	0.01673	0.00000	0.00000
11H	4.07421	0.01274	0.46474	0.01179	0.00000	0.00000

CALCULATED INTERNALS:

!
! Bond lengths
!

3N 9O = 2.93959 +- 0.00177
3N 10H = 1.97724 +- 0.00724
8H 9O = 2.85296 +- 0.00298

!
! Bond angles
!

9O 10H 3N = 168.94765 +- 1.38434
10H 3N 2C = 95.62338 +- 0.48867
11H 9O 3N = 112.71112 +- 0.73971
9O 3N 2C = 91.97974 +- 0.10150
3N 9O 10H = 7.40871 +- 0.91081
9O 3N 1S = 129.84787 +- 0.09450

Table S28- NBO stabilization energy contributions (≥ 0.21 kJ mol^{-1}) of 4-MT···H_2O and 5-MT···H_2O calculated at the B3LYP(D3BJ)/aug-cc-pVTZ level of theory.

4-MT···H_2O			
Donor NBO	Acceptor NBO	$E^{(2)}$ (kcal mol^{-1})	$E^{(2)}$ (kJ mol^{-1})
σ(S1-C2)	σ*(H_b-O)	0.13	0.54
σ(C4-C5)	σ*(H_b-O)	0.09	0.38
**LP(1) N3**	**σ*(H_b-O)**	**9.02**	**37.74**
σ(H_b-O)	σ*(C2-N3)	0.10	0.42
LP(2) O	σ*(C6-H6)	0.10	0.42
5-MT···H_2O			
Donor NBO	Acceptor NBO	$E^{(2)}$ (kcal mol^{-1})	$E^{(2)}$ (kJ mol^{-1})
σ(S1-C2)	σ*(H_b-O)	0.09	0.38
σ(C4-C5)	σ*(H_b-O)	0.09	0.38
**LP(1) N3**	**σ*(H_b-O)**	**8.58**	**35.90**
σ(H_b-O)	σ*(N3-C4)	0.17	0.71
LP(2) O	σ*(S1-C2)	0.06	0.25
